# Japanese Clinical Practice Guidelines for Vascular Tumors, Vascular Malformations, Lymphatic Malformations, and Lymphangiomatosis 2022

**DOI:** 10.53045/jprs.2025-0205

**Published:** 2026-03-26

**Authors:** Yoshiaki Kinoshita, Kosuke Ishikawa, Sadanori Akita, Katsuyoshi Koh, Satoru Sasaki, Masatoshi Jinnin, Hidefumi Mimura, Keigo Osuga, Michio Ozeki, Michiko Nagahama, Akihiro Fujino, Yoko Aoki, Akiko Asai, Noriko Aramaki-Hattori, Ryohei Ishiura, Masanori Inoue, Yuki Iwashina, Takafumi Ohshiro, Keiko Ogawa, Mine Ozaki, Junko Ochi, Shiro Onozawa, Motoi Kato, Takahide Kaneko, Tamihiro Kawakami, Akira Kitagawa, Masakazu Kurita, Yoshihiro Kuwano, Taro Kono, Shien Seike, Shinsuke Takagi, Nobuyuki Takakura, Takao Tachibana, Shuichi Tanoue, Kumiko Chuman, Hiroki Nakaoka, Yasuhiro Nakamura, Fumio Nagai, Yasunari Niimi, Shunsuke Nosaka, Taiki Nozaki, Tadashi Nomura, Kazuki Hashimoto, Ayato Hayashi, Satoshi Hirakawa, Takeshi Hirabayashi, Taizo Furukawa, Hiroshi Furukawa, Yumiko Hori, Takanobu Maekawa, Kentaro Matsuoka, Hideki Mori, Eiichi Morii, Akira Morimoto, Yuta Moriwaki, Shunsuke Yuzuriha, Naoaki Rikihisa, Munezumi Fujita, Yasuyuki Yamahana, Kyoichi Deie, Asami Tozawa, Daisuke Hasegawa, Akira Higashiyama, Daisuke Maeda, Sachiko Asayama, Yuhki Arai, Yohei Iwata, Mayu Uka, Hidehito Usui, Mizuki Uchiyama, Saori Endo, Hideki Endo, Rintaro Ono, Naoya Oshima, Toshihiro Otsuka, Kuniaki Ohara, Shinji Kagami, Tomo Kakihara, Mototoshi Kato, Hiroki Kanamori, Masafumi Kamata, Ami Kawaguchi, Akiko Kishi, Hiroshi Kitagawa, Kiyokazu Kim, Tamotsu Kobayashi, Takeshi Saito, Yusuke Shikano, Shuichi Shimada, Keisuke Suzuki, Masataka Takahashi, Shohei Takami, Reiko Takeda, Aya Tanaka, Kaishu Tanaka, Satoru Tamura, Masashi Tamura, Kanako Danno, Kenji Tsuboi, Yuta Nakajima, Ryo Nakatani, Miho Noguchi, Akifumi Nozawa, Naoki Hashizume, Masashi Hayakawa, Daichi Hayashi, Takaya Fukumoto, Mamoru Honda, Norifumi Matsuda, Hayato Maruguchi, Naoki Murakami, Kiichiro Yaguchi, Shiho Yasue, Hiroki Yoshihara, Rika Yoshimatsu, Kiyohito Yamamoto, Shinji Wada

**Affiliations:** 1 Department of Pediatric Surgery, Niigata University, Niigata City, Japan; 2 Department of Plastic and Reconstructive Surgery, Faculty of Medicine and Graduate School of Medicine, Hokkaido University, Sapporo City, Japan; 3 Department of Plastic Surgery, Tamaki Aozora Hospital, Tokushima City, Japan; 4 Department of Pharmacology, Fukushima Medical University, Fukushima City, Japan; 5 Department of Hematology/Oncology, Saitama Prefectural Children’s Medical Center, Saitama City, Japan; 6 Center for Vascular Anomalies, Department of Plastic and Reconstructive Surgery, Tonan Hospital, Sapporo City, Japan; 7 Department of Dermatology, Wakayama Medical University, Wakayama City, Japan; 8 Department of Radiology, St. Marianna University School of Medicine, Kawasaki City, Japan; 9 Department of Diagnostic and Interventional Radiology, St. Marianna University School of Medicine, Kawasaki City, Japan; 10 Department of Diagnostic Radiology, Osaka Medical and Pharmaceutical University, Takatsuki City, Japan; 11 Department of Pediatrics, Gifu University Hospital, Gifu City, Japan; 12 Department of Dermatology, Kobe Hokuto Hospital, Kobe City, Japan; 13 Department of Dermatology, Kobe University Hospital, Kobe City, Japan; 14 Department of Pediatric Surgery, National Center for Child Health and Development, Tokyo, Japan; 15 Department of Pediatric Surgery, Keio University School of Medicine, Tokyo, Japan; 16 Department of Medical Genetics, Tohoku University School of Medicine, Sendai City, Japan; 17 Department of Plastic and Reconstructive Surgery, Aichi Medical University, Nagakute City, Japan; 18 Department of Plastic and Reconstructive Surgery, Kainan Hospital, JA, Yatomi City, Japan; 19 Department of Plastic and Reconstructive Surgery, Keio University School of Medicine, Tokyo, Japan; 20 Department of Plastic and Reconstructive Surgery, Graduate School of Medicine, Mie University, Tsu City, Japan; 21 Department of Radiology, Keio University School of Medicine, Tokyo, Japan; 22 Department of Radiology, Fujita Health University, Toyoake City, Japan; 23 Department of Plastic Surgery, Kyorin University School of Medicine, Mitaka City, Japan; 24 Department of Plastic Surgery, Kyorin University School of Medicine Suginami Hospital, Tokyo, Japan; 25 Ohshiro Clinic, Tokyo, Japan; 26 Kampo Medicine Center, Hiroshima University Hospital, Hiroshima City, Japan; 27 Department of Diagnostic Radiology, Suita Tokushukai Hospital, Suita City, Japan; 28 Department of Radiology, Kyorin University School of Medicine, Mitaka City, Japan; 29 Department of Plastic and Reconstructive Surgery, Graduate School of Medicine, The University of Tokyo, Tokyo, Japan; 30 Department of Plastic and Reconstructive Surgery, Kagoshima University Hospital, Kagoshima City, Japan; 31 Department of Dermatology, Juntendo University Urayasu Hospital, Urayasu City, Japan; 32 Department of Dermatology, Tohoku Medical and Pharmaceutical University, Sendai City, Japan; 33 Kawakami Dermatology Clinic, Matsudo City, Japan; 34 Department of Radiology, Aichi Medical University, Nagakute City, Japan; 35 Department of Dermatology, Mizonokuchi Hospital, Teikyo University School of Medicine, Kawasaki City, Japan; 36 Department of Plastic Surgery, Tokai University School of Medicine, Isehara City, Japan; 37 Department of Plastic Surgery, Graduate School of Medicine, The University of Osaka, Suita City, Japan; 38 Department of Plastic and Reconstructive Surgery, Showa University Fujigaoka Hospital, Tokyo, Japan; 39 Department of Signal Transduction, Research Institute for Microbial Diseases, Osaka University, Suita City, Japan; 40 Department of Dermatology, Hoshigaoka Medical Center, Hirakata City, Japan; 41 Department of Dermatology, Hirakata Kohsai Hospital, Hirakata City, Japan; 42 Department of Radiology, Kurume University School of Medicine, Kurume City, Japan; 43 Department of Dermatology, Kanto Central Hospital, Tokyo, Japan; 44 Department of Plastic and Reconstructive Surgery, Ehime University Hospital, Toon City, Japan; 45 Department of Plastic & Reconstructive Surgery, Minami Matsuyama Hospital, Matsuyama City, Japan; 46 Department of Skin Oncology/Dermatology, Saitama Medical University International Medical Center, Hidaka City, Japan; 47 Department of Plastic and Reconstructive Surgery, Shinshu University School of Medicine, Matsumoto City, Japan; 48 Department of Neuroendovascular Therapy, St. Luke’s International Hospital, Tokyo, Japan; 49 Department of Radiology, National Center for Child Health and Development, Tokyo, Japan; 50 Department of Radiology, St. Luke’s International Hospital, Tokyo, Japan; 51 Department of Radiology, National Defense Medical College, Tokorozawa City, Japan; 52 Department of Plastic Surgery, Kobe University Graduate School of Medicine, Kobe City, Japan; 53 Department of Plastic Surgery, National Hospital Organization Kobe Medical Center, Kobe City, Japan; 54 Department of Plastic and Reconstructive Surgery, School of Medicine, Yokohama City University, Yokohama City, Japan; 55 Department of Nano Suit, Hamamatsu University School of Medicine, Hamamatsu City, Japan; 56 Department of Pediatric Surgery, Hirosaki University Hospital, Hirosaki City, Japan; 57 Department of Pediatric Surgery, Kyoto Prefectural University of Medicine, Kyoto City, Japan; 58 Department of Central Laboratory and Surgical Pathology, NHO Osaka National Hospital, Osaka City, Japan; 59 Department of General Pediatrics and Interdisciplinary Medicine National Center for Child Health and Development, Tokyo, Japan; 60 Department of Diagnostic Pathology, Tokyo Metropolitan Children’s Medical Center, Fuchu City, Japan; 61 Department of Pathology, Osaka University Graduate School of Medicine, Suita City, Japan; 62 Department of Pediatrics, Jichi Medical University, Shimotsuke City, Japan; 63 Department of Plastic Surgery, Oyumino Central Hospital, Chiba City, Japan; 64 Department of Plastic and Reconstructive Surgery, Fukushima Medical University, Fukushima City, Japan; 65 Shinwa Clinic, Tokyo, Japan; 66 Department of Radiology, Kyoto Prefectural University of Medicine, Kyoto City, Japan; 67 Department of Diagnostic Radiology, Japanese Red Cross Kyoto Daini Hospital, Kyoto City, Japan; 68 Department of Pediatric Surgery, Saitama Prefectural Children’s Medical Center, Saitama City, Japan; 69 Department of Pediatrics, St. Luke’s International Hospital, Tokyo, Japan; 70 Department of Plastic and Reconstructive Surgery, Osaka Rosai Hospital, Sakai City, Japan; 71 Department of Plastic Surgery, Toyonaka Municipal Hospital, Toyonaka City, Japan; 72 Department of Dermatology, Nippon Medical School Hospital, Tokyo, Japan; 73 Department of Dermatology, Fujita Health University, Toyoake City, Japan; 74 Department of Radiology, Okayama University Hospital, Okayama City, Japan; 75 Department of Radiology, NHO Okayama Medical Center, Okayama City, Japan; 76 Department of Surgery, Kanagawa Children’s Medical Center, Yokohama City, Japan; 77 Department of Plastic and Reconstructive Surgery, Juntendo University Urayasu Hospital, Urayasu City, Japan; 78 Department of Advanced Medicine for Refractory Vascular Anomalies, Graduate School of Medicine, Gifu University, Gifu City, Japan; 79 Department of Dermatology, Osaka General Hospital of West Japan Railway Company, Osaka City, Japan; 80 Department of Dermatology, Osaka Medical and Pharmaceutical University Hospital, Takatsuki City, Japan; 81 Otsuka Dermatology Clinic, Kobe City, Japan; 82 Department of Dermatology, Akasaka Toranomon Clinic, Tokyo, Japan; 83 Department of Pediatric Surgery, The University of Tokyo Hospital, Tokyo, Japan; 84 Department of Pediatric Surgery, Keio University, Tokyo, Japan; 85 Department of Pediatric Surgery, School of Medicine, Fujita Health University, Toyoake City, Japan; 86 Department of Dermatology, Toranomon Hospital, Tokyo, Japan; 87 Department of Dermatology, Mie University Hospital, Tsu City, Japan; 88 Department of Dermatology, Kumamoto University Hospital, Kumamoto City, Japan; 89 Department of Pediatric Surgery, Faculty of Medicine, Toho University, Tokyo, Japan; 90 Department of Pediatric Surgery, Japanese Red Cross Medical Center, Tokyo, Japan; 91 Esaka-ekimae Hanafusa Dermatology & Beauty Clinic, Suita City, Japan; 92 Department of Pediatric Surgery, Kagawa University, Kagawa, Japan; 93 Department of Diagnostic and Interventional Radiology, Osaka University Graduate School of Medicine, Suita City, Japan; 94 Department of Plastic Surgery, National Defense Medical College Hospital, Tokorozawa City, Japan; 95 Shiromoto Clinic, Yokkaichi City, Japan; 96 Department of Pediatric Nephrology, Tokyo Women’s Medical University, Tokyo, Japan; 97 Department of Medical Genetics, Tohoku University, Sendai City, Japan; 98 Department of Pediatric Surgery, Kurume University, Kurume City, Japan; 99 Department of Plastic and Reconstructive Surgery, Nagoya Ekisaikai Hospital, Nagoya City, Japan; 100 Fukumoto Dermatopathology Clinic, Nara City, Japan; 101 Maruguchi Skin Clinic, Kobe City, Japan; 102 Department of Plastic Surgery, Nagano Children’s Hospital, Azumino City, Japan; 103 Department of Diagnostic and Interventional Radiology, Kochi University Medical School, Nankoku City, Japan; 104 Department of Radiology, Kochi Health Sciences Center, Kochi City, Japan

**Keywords:** clinical practice, guidelines, hemangioma, vascular anomalies, vascular malformation

## Abstract

The objective was to prepare guidelines to perform the current optimum treatment by organizing effective and efficient treatments of hemangiomas and vascular malformations, confirming the safety, and systematizing treatment, employing evidence-based medicine techniques and aimed at improvement of the outcomes. Clinical questions (CQs) were decided based on the important clinical issues. For document retrieval, key words for literature searches were set for each CQ and literature published from 1980 to the end of December 2020 was searched in PubMed, and Japana Centra Revuo Medicina (JCRM). The strengths of evidence and recommendations acquired by systematic reviews were determined following the Medical Information Network Distribution Service (Minds) technique. A total of 38 CQs were used to compile recommendations and the subjects included efficacy of resection, sclerotherapy/embolization, drug therapy, laser therapy, radiotherapy, and other conservative treatment, differences in appropriate treatment due to the location of lesions and among symptoms, appropriate timing of treatment and tests, pathological diagnosis deciding the diagnosis, and causal genes of vascular anomalies. Thus, the Japanese Clinical Practice Guidelines for Vascular Tumors, Vascular Malformations, Lymphatic Malformations, and Lymphangiomatosis 2022 have been prepared as the evidence-based guidelines for the management of vascular anomalies.

## Introduction

The etiology of vascular anomalies on the body surface and in soft tissue are mostly unclear and no fundamental treatment methods have been established. Many patients visit many medical institutions seeking an expert, being a disadvantage in treatment. Hemangiomas and vascular malformations are frequently termed ‘hemangioma’, but these are different diseases in the ISSVA classification proposed by the International Society for Study of Vascular Anomalies (ISSVA)^1)^, and this classification has been internationally standardized.

“Clinical Practice Guidelines for Vascular Anomalies 2013” (1st edition)^2)^ target general practitioners and the general public, and were prepared aiming at organizing effective and efficient treatments for hemangiomas/vascular malformations, confirming the safety, and systematizing treatment using evidence-based medicine (EBM) techniques. The organization responsible for preparation was the Health, Labour and Welfare Sciences Research Grants (Research on Measures for Intractable Diseases), Research Committee for “Intractable Vascular Anomalies,” and the main committee members were selected from academic societies of plastic surgery and radiology mainly treating hemangiomas and vascular malformations: the Japanese Society of Plastic and Reconstructive Surgery and Japanese Society of Interventional Radiology, and the guidelines were prepared by them.

“Japanese Clinical Practice Guidelines for Vascular Anomalies 2017” (2nd edition)^3)^ were prepared as a revised edition of the “Clinical Practice Guidelines for Vascular Anomalies 2013.” The organization responsible for preparation was the Health, Labour and Welfare Sciences Research Grants (Research on Policy Planning and Evaluation for Rare and Intractable Diseases), Research Committee for Intractable Vascular Anomalies, and the differences from the previous guidelines are setting the objective at summarizing opinions from related academic societies by inviting many committee members from dermatologists, pediatric surgeons, pediatricians, radiologists (diagnostic radiology), and basic researchers including the pathology, molecular biology, and epidemiology fields, in addition to plastic surgeons and radiologists (interventional radiology). Since the guidelines were prepared following the reformed “Minds Handbook for Clinical Practice Guideline Development 2014”^4)^ and “Minds Manual for Clinical Practice Guideline Development Ver.1.0–2.0,”^5,6)^ it was fully revised.

“Japanese Clinical Practice Guidelines for Vascular Tumors, Vascular Malformations, Lymphatic Malformations, and Lymphangiomatosis 2022” (3rd edition) was revised from the 2nd edition. The project was started from 2020 supported by the Health, Labour and Welfare Sciences Research Grants (Research on Policy Planning and Evaluation for Rare and Intractable Diseases), Research Committee for Intractable Vascular Anomalies. New findings from the 2nd edition onwards were added, and consideration was given to what should be continued, what should be revised, and what should be newly added, resulting in a total of 38 clinical questions (CQs). The guidelines were prepared following the reformed “Minds Manual for Guideline Development 2017”.

The original text of the guidelines (Japanese version) is comprised of Reviews and Clinical Questions, but only CQs are presented in this report.

## Purpose of the Guidelines

The objective was to prepare guidelines to perform the current optimum treatment by organizing effective and efficient treatments of hemangiomas and vascular malformations, confirming the safety, and systematizing treatment, employing the EBM techniques and aimed at improvement of the following outcomes: pain, swelling, esthetic impairment, and functional disorder.

## Materials and Methods

### Organization

For the Guidelines Executive Committee members, representatives of the plastic surgery, dermatology, radiology, pediatric surgery, and basic science fields were selected. The guidelines preparation group and systematic review team for preparation of CQs and recommendations were comprised of 4 groups: groups in charge of arteriovenous malformation (AVM), venous malformation (VM), combined type, and syndrome, in charge of capillary malformation (CM) and infantile hemangioma, in charge of the lymphatic malformation (lymphangioma)(LM), and in charge of the basic field. To the group in charge of AVM, VM, combined type, and syndrome, plastic surgeons and radiologists were mainly assigned. To the group in charge of CM and infantile hemangioma, plastic surgeons and dermatologists were mainly assigned. To the group in charge of the lymphatic system, pediatric surgeons, plastic surgeons, and pediatricians were mainly assigned. The reviews of the guidelines were also prepared by those selected from each group. Pathologists and molecular biologists were in charge of the reviews of the basic fields.

### Preparation process

The guidelines were revised following the Minds Manual for Guideline Development 2017^6)^.

CQs were decided based on the following important clinical issues: (i) Efficacy of resection, (ii) efficacy of sclerotherapy/embolization, (iii) efficacy of drug therapy, laser therapy, radiotherapy, and other conservative treatment, (iv) differences in appropriate treatment due to the location of lesions, (v) differences in appropriate treatment among symptoms, (vi) appropriate timing of treatment and tests, (vii) pathological diagnosis deciding the diagnosis, and (viii) causal genes of vascular anomalies.

For document retrieval, key words for literature searches were set for each CQ and literature published from 1980 to the end of December 2020 was searched in PubMed, and Japana Centra Revuo Medicina (JCRM). Literature search was requested to the Japan Medical Library Association. For decisions on CQs and recommendations lacking evidence or having weak evidence, discussion and agreement in the preparation group were reflected.

The strengths of evidence and recommendations acquired by systematic reviews were determined following the Minds technique as described below and this follows the GRADE guidelines preparation method^7,8)^.

### Determination of the strength of evidence of the body of evidence

The Strength of Evidence of the Body of Evidence was determined according to the Minds Manual for Guideline Development 2017 (Table 1)^9)^.

In the case of randomized controlled trials (RCTs), the score “A (strong)” is given at the start of evaluation, and the final score might be downgraded to B, C, or D, according to the results of evaluation of five items, including risk of bias, inconsistency in results, indirectness of evidence, data imprecision, and high possibility of publication bias. In the case of observational studies, the score “C (weak)” is given at the start of evaluation, and five items lowering the strength are evaluated similarly as for RCTs. In addition, three items, including large effect with no confounding factors, dose-response gradient, and possible confounding factors, are weaker than actual effects increasing the strength are evaluated as well.

**Table 1.** Results of clinical studies with 50 or more subjects.

Reference No.	Author(Year)	Country	Sclerosing agent	Study design	Site of lesion	No.(Age.)	Outcome rate (%)				Observation period
							Size reduction	Symptoms	Recurrence rate	Severe complications	
137	Bianchini(2018)	Italy	Ethanol Liquid polidocanol Or both	Retrospective	Limbs (in the muscle)	81(3-45 yr.)	16	89	14	1	3-52 mo.(Ave. 26 mo.)
139	Yun(2009)	Korea	Ethanol	Retrospective	Head/neck, trunk, limbs	158(1-58 yr.)	27	48	Unclear	3	Unclear
142	Orlando (2010)	Brazil	Ethanol	Prospective	Head/neck, trunk, limbs	81(8-68 yr.)	93	95	Unclear	0	3-72 mo.(Median: 18 mo.)
143	Stuart(2015)	U.K.	STS foam	Retrospective	Unclear	204(6 mo.-18 yr.)	Unclear	85	Unclear	2	6-8 w
150	Park(2016)	Korea	STS foam	Retrospective	Head/neck, trunk, limbs	86(6-56 yr.)	53	50	13	0	6-71 mo.(Median: 27 mo., Ave 28 mo.)
152	Kuramoto(2018)	Japan	Liquid EO EO foam Polidocanol foam	Retrospective	Head/neck, trunk, limbs	187(unclear)	Unclear	71	Unclear	0	>3 mo.
154	Jin(2008)	China	Ethanol Bleomycin	Retrospective	Head/neck, trunk, limbs	201(Ave 14 yr.)	Unclear	94	Unclear	0	12-65 mo.(Ave 29 mo.)
157	Zhao(2004)	China	Bleomycin Morrhuate sodium Or both	Retrospective	Head/neck	260(unclear)	88	Unclear	Unclear	0	6 mo.-5 yr.
159	Lee(2009)	Korea	Ethanol	Retrospective	Head/neck	87(2-68 yr.)	85	Unclear	Unclear	0	10 days-120 mo. (Ave 35 mo., Median: 28 mo.)
161	Bai(2014)	China	Bleomycin	Retrospective	Head/neck, trunk, limbs, perineal region	184(13-65 yr.)	97	Unclear	Unclear	Unclear	12-16 mo.
162	Zhi(2008)	China	Bleomycin	Retrospective	Head/neck	82(5-14 yr.)	96	Unclear	Unclear	0	>2 yr.
167	Lee(2003)	Korea	Ethanol	Retrospective	Limbs	87(Ave. 22 yr.)	Unclear	Unclear	0	13	Ave 24 mo.
155	Khaitovich(2019)	Israel	Ethanol Ethanol and bleomycin or polidocanol	Retrospective	Head/neck, trunk, limbs, gluteal region, perineal region	153(6 mo.-67 yr.)	Unclear	70	Unclear	8	4-122 mo.(Ave 44 mo.)

### Presentation of the strength of recommendations

The strength of recommendation was also determined according to the Minds Manual for Guideline Development 2017 (Table 1)^9)^.

The strength of recommendations is usually presented in two ways:“1”: strongly recommended, and “2”: weakly recommended (suggested). If the strength of recommendations cannot be determined by any means, it is occasionally presented as “no definite recommendation can be made.” Recommendations will be entered as follows by indicating the strength of evidence (A, B, C, D) with the strength of recommendations “1”: strong or “2”: weak.

### Finalization

The draft guidelines were completed in November 2022 and review was requested to the Japan Society of Plastic and Reconstructive Surgery, Japanese Dermatological Association, Japan Radiological Society, Japanese Society of Interventional Radiology, Japan Pediatric Society, Japanese Society of Pediatric Surgeons, the Molecular Biology Society of Japan, and some members of Japanese Society of Pathology. In addition, from December 2022 to January 2023, the guidelines were disclosed on the home page of the Research Committee for ‘Intractable Vascular Anomalies’ and public comments were collected. The draft guidelines were presented to four related patient organizations, ‘Patient Advocate of Vascular Anomalies Japan’, ‘Patients Association of Combined Vascular Malformations’, ‘Vascular Malformations Network’ and ‘NPO International Lymphatic Malformations Network’ and comments were received. Based on these, the draft guidelines were revised and CQs, recommendations, and explanations were completed. It was finalized in January 2023.

## Results

### CQs and recommendations

### CQ1: What are the guidelines for the time to begin treatment for AVM?

Recommendation: It is necessary to determine the time to start surgical resection or interventional radiology treatment for AVM individually in consideration of the stage of symptoms, location of the lesion, and extent of involvement and with comprehensive evaluation of the effects expected from the treatment, and the risk of complications associated with the treatment. However, AVM often follows a progressive course accompanied by progression of functional impairment depending on its site. Since the treatment becomes more difficult with the progression of the disease stage, early therapeutic intervention should be evaluated for early cases in consideration of the risk of decline of quality of life (QOL) due to treatment-related complications.

Strength of recommendation: 2 (weak): Recommended to be performed

Evidence: D (very weak)

#### Process of preparation of recommendation

##### 1．Circumstances that make CQ an important clinical issue

AVM often shows a progressive course. Generally, while treatment of small lesions with a simple vascular architecture is relatively easy, treatment becomes more difficult as the lesion progresses and begins to show a complicated vascular architecture. Treatments for AVM include surgery, transcatheter embolization, and percutaneous sclerotherapy, and satisfactory therapeutic effects can be expected from both radical and palliative treatments. On the other hand, the risk of treatment-related complications is not negligible, and severe sequelae or functional impairment may remain depending on the site or severity of the lesion. Therefore, the judgment about the time of therapeutic intervention is extremely important, and it is an important clinical issue as the evaluation and clarification of appropriate time to start treatment may contribute to improvements in the therapeutic effect and prevention of complications.

##### 2．Evaluation of evidence

###### <Literature search>

As a result of a systematic literature search, 637 papers (589 from PubMed, and 48 from JCRM) were selected for primary screening, from which 91 papers were extracted. As a result of secondary screening, 45 papers that evaluated or mentioned the time of the beginning of treatment were extracted. Since all these papers were reports of case series studies or observational studies, their evidence level is judged to be D (very weak).

###### <Evaluation>

Of the extracted references, none evaluated the time of the beginning of treatment as a primary endpoint, and some mentioned the Schobinger stage and age at the beginning of treatment, course of examinations, and outcomes of treatment. Therefore, objective assessment of the validity of the time of the beginning of treatment are difficult. We, thus, evaluated whether a reasonable view about the judgment about the time of the beginning of treatment and its validity can be obtained based on the symptoms, age, affected areas, degree of progression, therapeutic outcomes, and descriptions about complications of the subjects in each reference.

Concerning the therapeutic methods, among the references that mentioned matters related to the time of treatment, there are reports on surgical resection (and aesthetic reconstruction)^10-13)^, embolization^14-35)^, and their combination^36-52)^, and, although small in number, reports on laser treatment^53)^ and drug therapy^54)^. Embolization is performed by either the transcatheter technique (transarterial or transvenous approach) or direct percutaneous puncture, and the materials reported to have been used vary: n-butyl-2-cyanoacrylate, ethanol, Onyx, metal coils, microspheres, gelatin sponge, and polyvinyl alcohol particles and their combination^14-52)^.

AVM often exhibits a progressive course, and the judgment about the time of therapeutic intervention is extremely important. According to a reference that also evaluated the natural course from infancy, more than 80% of the children with Schobinger stage I disease show progression before adolescence, and the progression is by 2 or more stages in many patients^40)^. In addition, the recurrence rate after treatment is lower as the treatment is started at lower stages, and early resection should be considered positively in low-stage cases^40,49)^. Also, there are reports that, if the lesion is localized, complete cure can be expected by early treatment^14,42)^. However, concerning ethanol embolization, while higher cure rates have been reported, the complication rates have also been high, and, because of this countervailing of benefits and risks, careful judgment is needed in early therapeutic intervention^22,24)^.

Although some reports recommended early therapeutic intervention, exacerbation may not be observed in children with Schobinger stage I lesions until adolescence, and it is important to remember that there are many cases in which therapeutic intervention can be postponed until adolescence^40)^. Furthermore, complications due to radical treatment or damage due to treatment, such as tissue defect, are difficult to accept for young patients, and evaluation of palliative treatment is also important^46)^. Also, concerning the evaluation of the relationship between the Schobinger stage and therapeutic effect, the therapeutic effect (resection rate and embolization rate) is higher with significantly greater improvements in symptoms as the stage is lower regardless of age^10,38)^.

Judgements according to the site of the lesion are also important. In orbital lesions, since a delay of therapeutic intervention may lead to a decline in functional outcome (visual acuity), and since satisfactory aesthetic reconstruction may not be obtained in older patients, early intervention is recommended^39)^. Regarding the literature concerning AVM of the trunk and limbs, the presence or absence of symptoms is generally important, but as AVM progresses if left untreated and becomes difficult to treat, and as the cure rate by treatment is high with a high likelihood of a favorable clinical outcome in type I or II lesions according to the Cho-Do classification by initial angiography, aggressive therapeutic intervention is recommended^24)^. In head and neck lesions, treatments exert large effects on not only aesthetic aspects but also important functions including breathing and eating, and there have been reports of cases of lingual AVM that developed severe complications, which required a tracheostomy or airway reconstruction due to post-treatment airway obstruction^17)^. Lower limb AVM is frequently accompanied by ulcer due to circulation disorder under the influence of gravity but rarely progresses to Schobinger stage IV. Therefore, judgments about therapeutic intervention should be made in consideration of the extent of involvement and responses to, and invasiveness of, treatment (the therapeutic effectiveness is high in lesions involving ≤ 25% of the affected limb)^22)^.

###### <Conclusion>

From the description in these references, it is impossible to make clear recommendation about the time of therapeutic intervention and inevitable to conclude that judgments should be made comprehensively by sufficiently evaluating the site of the lesion, symptoms, age, Schobinger stage, effects and complications expected from treatment, and the risks of exacerbation of symptoms and deterioration of functional outcome associated with postponement of treatment.

##### 3．Evaluation of balance of benefits and risks

The benefits regarding this CQ are that symptoms are alleviated or resolved, and normal functions and aesthetic appearance are preserved, by treating AVM by surgery, transcatheter or percutaneous embolization, or their combination with appropriate judgment about the time of therapeutic intervention. However, possible risks are that misjudgment of the time of therapeutic intervention leads to exacerbation of symptoms, functional or aesthetic abnormalities, and greater difficulties of treatment, or increases in the risk of complications by making therapeutic intervention in a more difficult situation. Moreover, exacerbation of symptoms, functional impairment, and complications caused by inappropriate early intervention for asymptomatic or mildly symptomatic lesions that do not or only slowly progress are also possible risks. Balance of these benefits and risks must be taken into consideration in the assessment of the strength of recommendation about this CQ.

##### 4．Patients’ values/wishes

The judgment about the time of therapeutic intervention is considered to conform to the values and wishes of many patients from the viewpoint of expectations for the therapeutic effects and prevention of progression of the disease and complications.

##### 5．Cost assessment, assessment of external validity of intervention

Since the judgment about the time of therapeutic intervention related to this CQ is made simultaneously with collection of clinical information, such as the symptoms and course and general examinations covered by National Health Insurance in Japan, it does not generate any additional cost.

#### Lay summary

Although AVM is a benign vascular lesion, it often shows a progressive course, which makes the judgement about the time of treatment extremely important. However, as its symptoms and degree of progression vary widely, it is difficult to set fixed judgment criteria, and careful individual judgments according to the location of the lesion, symptoms, degree of progression, and vascular architecture are necessary.

### CQ2: Is proximal ligation/coil embolization of the feeding artery of AVM effective?

Recommendation: Although proximal ligation/coil embolization of the feeding artery of AVM may temporarily lead to a reduction in the lesion size, many lesions recur by complicated collateral channels with symptomatic exacerbation and make treatment more difficult. Moreover, ligation/embolization is not recommendable except before surgery or for AVM with a special shape such as arteriovenous fistula.

Strength of recommendation: 1 (strong): Recommended not to be performed

Evidence: D (very weak)

#### Process of preparation of recommendation

##### 1．Circumstances that make CQ an important clinical issue

Proximal ligation/coil embolization of the feeding artery may be performed as a treatment for AVM. However, symptoms have been reported to deteriorate rather than improve in many patients. In addition, even if a temporary reduction of the lesion size may be achieved, lesions have been reported to thereafter recur or be exacerbated by complicated collateral channels and become more difficult to treat. For this reason, it is important to understand that ligation/embolization of the feeding artery should be avoided except before surgery or for lesions with a special shape such as arteriovenous fistula. This CQ is important to prevent such treatment from being performed without careful consideration.

##### 2．Evaluation of evidence

###### <Literature search>

As a result of systematic literature search, 503 papers (251 from PubMed, 252 from JCRM) were selected for primary screening. Also, as a result of hand search, 3 papers (3 from PubMed, 0 from JCRM) were selected for primary screening. As a result of primary screening, 64 papers (27 from PubMed, 37 from JCRM) were selected for secondary screening. As a result of secondary screening, 16 papers (8 from PubMed, 8 from JCRM) were adopted. All were reports of case series studies or case reports, and the evidence level of these references was rated low because of the limitations of the study design.

###### <Evaluation>

The objective of treatment for AVM is the elimination of the nidus by either resection or embolization. It has been reported that the elimination of the nidus cannot be achieved by proximal ligation/embolization of the feeding artery and that the lesion or the symptoms remained unchanged or were exacerbated^46,55-59)^, presumably because proximal ligation/embolization of the feeding artery leads to the development of complex collateral channels^46,58-63)^. In many cases, collateral channels are reported to be complicated and markedly tortuous, making transcatheter treatment difficult^61,62,64,65)^.

Wu *et al*.^46)^ performed proximal ligation in 9 of the 29 patients with AVM of the auricle and reported that the condition was exacerbated in all patients, requiring auricular resection in 8 and additional treatment in 1, and that proximal ligation should be excluded as a treatment for AVM. Slaba *et al*.^60)^ evaluated 25 cases of AVM affecting the tongue and reported that marked development of collaterals was observed in 3 of the 12 symptomatic cases because of ligation of the ipsilateral external carotid artery performed at other hospitals.

Although there are reports that ligation/embolization of the feeding artery before resection to reduce the blood flow of AVM is effective, its indication should be evaluated carefully^56,66,67)^.

Ligation/embolization of the feeding artery is effective for the treatment of arteriovenous fistula, which is a specific type of AVM, but it should be diagnosed carefully^68,69)^.

###### <Conclusion>

While ligation/embolization of the feeding artery of AVM may achieve a temporary reduction in the lesion size and symptomatic alleviation except before surgery or in arteriovenous fistula, which is a special type of AVM, it is expected to cause an increase in the lesion size and symptomatic exacerbation in the long run and make treatment more difficult.

##### 3．Evaluation of balance of benefits and risks

The recommendation about this CQ was prepared by focusing on the outcomes (O) set by evaluating PICO: O1 lesion size reduction, O2 alleviation of symptoms, O3 recurrence/recanalization, O4 exacerbation of symptoms, and O5 difficulty of re-treatment. A review of the literature revealed that O1 and O2 may be achieved immediately after treatment but that the treatment is likely to eventually lead to O3 and O4. Furthermore, O5 emerged as a problem after O3 and O4 due to the development of collateral channels.

##### 4．Patients’ values/wishes

Since this treatment may cause temporary alleviation of symptoms but is likely to eventually end in exacerbation of symptoms and make treatment more difficult, it is not considered to conform to the patients’ values/wishes.

##### 5．Cost assessment, assessment of external validity of intervention

Ligation/embolization is a treatment covered by National Health Insurance in Japan and has no problem regarding intervention or the cost.

#### Lay summary

Ligation/embolization of the feeding artery of AVM may cause a temporary reduction of the lesion size but often lead to recurrence by complicated collateral channels, resulting in exacerbation of symptoms. It may further make subsequent treatment more difficult. Therefore, this treatment is not recommendable except before surgery or for AVM with a specific type such as arteriovenous fistula.

### CQ3: Is angiographic classification useful in embolization of AVM?

Recommendation: Classification by angiography may be useful for the estimation of the complete cure rate or the necessary number of treatments. However, multiple classifications have been proposed, and, although their integration or interchange is not easy, treatment is more difficult as the anatomy of AVM is more complicated.

Strength of recommendation: 2 (weak): Recommended to be performed

Evidence: D (very weak)

#### Process of preparation of recommendation

##### 1．Circumstances that make CQ an important clinical issue

Since surgical resection of AVM involves the risk of massive arterial hemorrhage, embolization, which is an endovascular treatment, plays a large role. Recently, the development of angiographic catheters and improvement in the performance of imaging techniques have made it possible to delineate the morphology of AVM in greater detail, and reports of angiographic classifications and embolization based on them have increased. Embolization focused on the arteriovenous shunt portion is important to obtain satisfactory therapeutic effects, and embolization of inappropriate vessels may induce not only increased complexity of the re-treatment route and exacerbation of AVM but also complications associated with marked obstruction of normal vessels. Clarification of the target vessel of treatment and approach route to it by angiographic classification may lead to improvements in the therapeutic outcome and prevention of complications.

##### 2．Evaluation of evidence

###### <Literature search>

As a result of systematic literature search, 269 papers (107 from PubMed, 162 from JCRM) were selected for primary screening. (As a result of hand search, 2 papers from PubMed were selected for primary screening.) As a result of primary screening 28 papers (17 from PubMed, 11 from JCRM) were selected for secondary screening, through which 11 papers (10 from PubMed, 1 from JCRM) were adopted. By hand search, the classification by Houdart *et al*.^70)^ (angiographic classification of intracranial AVM), which is considered to have influenced the Cho-Do classification^29)^ and Yakes classification^71)^, and Yakes classification (review by Yakes himself)^71)^ were added. All these, except 1 paper on a retrospective cohort study, were case series studies, and the evidence level of these references was rated low because of the limitations of the study design.

###### <Evaluation>

**Figure 1:**
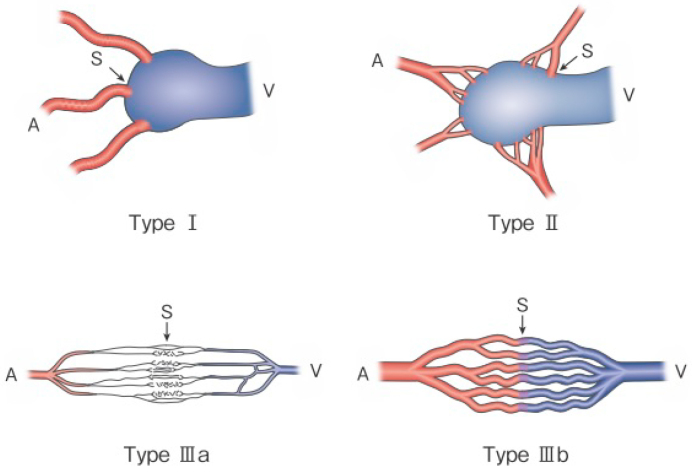
Revised morphological classification of AVM. Type I: 3 or less feeding arteries + 1 draining vein Type II: Multiple feeding arteries + 1 draining vein Type IIIa: Multiple non-dilated AV shunts Type IIIb: Multiple dilated AV shunts A: artery, S: shunt, V: vein (Kitagawa A, *et al.*: reprinted with permission from Jpn J Diag Imaging 2021;41:1253-60)

**Figure 2:**
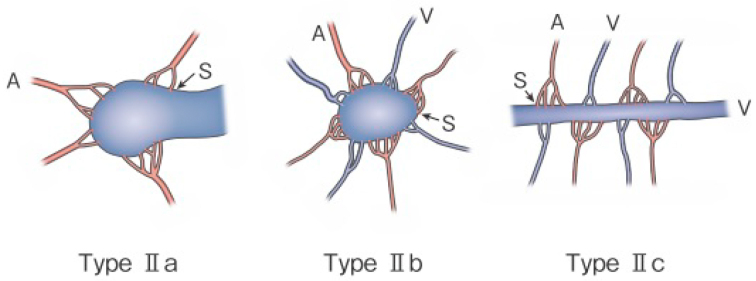
Subtyping of Type II AVM. Type IIa: Multiple feeding arteries + 1 draining vein Type IIb: Multiple feeding arteries + venous sac + multiple draining veins Type IIc: Multiple feeding arteries + long segment of draining vein A: artery, S: shunt, V: vein (Kitagawa A, *et al.*: reprinted with permission from Jpn J Diag Imaging 2021;41:1253-60)

The Cho-Do classification^29)^ and Yakes classification^71)^ are classifications based on angiography that are used relatively widely. There were other reports using the authors’ original classification methods and those in which the Houdart classification for intracranial AVM^70)^ was provisionally applied. Among those using the Cho-Do classification, according to the report by Cho *et al*.^29)^ targeting AVM of the trunk and limbs, ethanol embolization was effective for type II (100%), type IIIb (83%), and mixed type (≤50%) in descending order (Figure 1). They also performed direct puncture and transvenous embolization for type II, transarterial embolization only for type IIIa, and direct puncture and transarterial embolization for other types. According to the report by Park *et al*.^24)^ of the same group, the predictive factors of the clinical outcome were the extent of AVM (better in the localized type) and angiographic classification (better in type I and type II than in other types). Also, according to the report of subclassification of type II lesions by Ko *et al*.^72)^ of the same group, the complete cure rate was high in IIa, IIb, and IIc (95%, 76%, 65%) in descending order, and the number of treatments was higher in type IIc (median, 2.5) compared with type IIa or IIb (median, 1)(Figure 2). However, according to the report by Hyun *et al*.^22)^ of the same group, the outcome of ethanol embolization for foot AVM was not correlated with the Cho-Do classification.

According to the report about head and neck AVM using the Yakes classification by Griauzde *et al*.^73)^ and the report on peripheral AVM by Bouwman *et al*.^14)^, the number of treatments was significantly higher in type IV (innumerable microfistulae infiltrating the entire affected tissue) than in other types (mean, 3.6 vs. 1.9; median, 4). Also, according to Bouwman *et al.*, the blood flow reduction rate was significantly higher in types I and IIIa compared with types II, IIIb, and IV^14)^.

As for reports using the authors’ original classifications, according to the angiographic classification by direct puncture of maxillofacial AVM by Xun *et al*.^74)^, the therapeutic effect was greater by ethanol sclerotherapy with additional Gelfoam embolization on type III AVM (draining vein dilated to ≥6 mm) than by ethanol sclerotherapy alone. According to the report concerning AVM of the jaw by Liu *et al*.^45)^, type II, III, and IV lesions could be cured, but the cure rate was 60% in type I and 33.3% in type II. According to the report concerning scalp AVM by Gopinath *et al*.^75)^, the concentration of n-butyl-2-cyanoacrylate used for the treatment was higher, and the complete occlusion rate was also higher, in the fistulous type. According to the report on facial/head and neck AVM by Sugiu *et al*.^76)^, the therapeutic effect was higher in single AVF than in multiple AVF and AVM.

As a report provisionally using the Houdart classification, which is a classification of intracranial AVM, Yamada *et al.* treated 15 patients with head and neck AVM by repeated injection of a small amount of n-butyl-2-cyanoacrylate and obtained a relatively high response rate in type III^77)^.

As serious complications, conditions, such as skin necrosis, pseudoaneurysm, finger contracture, embolism, cerebral infarction, bladder necrosis, acute renal failure, and infection, have been reported. There have been reports that did not mention serious complications or denied their occurrence, but, in one report, their incidence was as high as 15.2%^29)^. The risk of complications may increase with the number of treatments, but it is not considered to differ according to age or the site of AVM. Griauzde *et al*.^73)^ reported that, of the 5 patients, complications occurred in 4 patients with Yakes type IV lesions (involving capillaries) and that surgical repair was necessary in 3 of them. Complications may be more likely to occur in some types, and further accumulation of reports is awaited.

##### <Conclusion>

The above references suggest that angiographic classification may be useful for the estimation of the cure rate and number of treatments needed. Although it is not easy to integrate or interchange classifications, treatment is more difficult as the anatomy of the arteries and veins is more complicated.

##### 3．Evaluation of balance of benefits and risks

The recommendation of this CQ was prepared by focusing on outcomes (O) 1 nidus occlusion, O2 symptomatic improvement, O3 reduction of lesion size, O4 presence or absence of recurrence (re-enlargement), and O5 serious treatment-related complications, which were set by evaluating PICO. Of these outcomes, O1 and O2 could not be distinguished in most cases. O3 was not mentioned in a half of reports and did not differ significantly in most of the reports that mentioned it. O4 was mentioned in few reports, and no significant difference was observed in the incidence among the types in the reports that mentioned it. Concerning O5, some types may be more likely to develop complications, and further accumulation of reports is awaited.

##### 4．Patients’ values/wishes

Since angiographic classification may be useful for the estimation of the cure rate and necessary number of treatments, it may make prediction of the future therapeutic effects and number of treatments by patients possible, and it is considered to comply with the patients’ values and wishes.

##### 5．Cost assessment, assessment external validity of intervention

Angiography is essential for classifying AVM, but it is often performed simultaneously with embolization, at no additional cost. An additional cost is incurred if angiography is performed separately.

#### Lay summary

In embolization of AVM, classification of lesions by angiography may be useful for the estimation of the cure rate and necessary number of treatments. However, multiple classifications have been proposed, and their integration or interchange is not easy, but treatment is more difficult as the anatomy of the arteries and veins is more complicated.

### CQ4: What treatments are appropriate for maxillomandibular AVM?

Recommendation: Radiotherapy or surgery alone is not recommended. Endovascular embolization (including sclerotherapy) alone or its combination with relatively less invasive surgery is recommended as necessary.

Strength of recommendation: 1 (strong): Recommended to be performed

Evidence: D (very weak)

#### Process of preparation of recommendation

##### 1．Circumstances that make CQ an important clinical issue

Maxillomandibular AVM is a rare disorder that involves the maxilla, mandible, or both and often presents with gingival hemorrhage around the age of 10 years when milk teeth are lost. Hemorrhage is often arterial and massive, and if temporary hemostasis may be achieved by compression, it often recurs if left untreated. Radical treatment for AVM of the maxillomandibular region is important for the prevention of hemorrhage. Conventionally, the disorder has been treated surgically, but the management of massive intraoperative bleeding has been a problem, and, if the mandible or maxilla has been resected, there has also been the risk of inhibition of the jaw development and impairment of mastication and speech functions. Recently, with the development of endovascular treatments, endovascular embolization has begun to be performed for this lesion, and the invasiveness of treatment has been reduced. Endovascular embolization may be performed alone or in combination with surgery, and its procedure and the embolic materials used for embolization also vary widely. Review of the current state of treatment and evaluation of recommendable treatments by literature search are important.

##### 2．Evaluation of evidence

###### <Literature search>

As a result of systematic literature search, 172 papers (95 from PubMed, 77 from JCRM) were selected for primary screening. As a result of primary screening, 52 papers were selected for secondary screening, which resulted in the adoption of 22 papers (20 from PubMed, 2 from JCRM). All these references are case/case-series reports, and the strength of evidence of the set of references concerning this CQ was rated as D (very weak).

###### <Evaluation>

The literature extracted by search was mostly case reports or case series reports primarily concerning endovascular treatments, most of the case series were relatively small, and none was about radiotherapy or surgery alone. Concerning endovascular embolization, there were reports concerning endovascular embolization performed alone^78-86)^, performed with additional surgical procedures as necessary^33,45,87-89)^, or performed preoperatively to reduce bleeding^12,90-94)^. Surgery performed after embolization was often a combination of relatively mildly invasive procedures including filling of bone wax and curettage^45,81,83,90-92)^. Regarding the methods for endovascular embolization, transarterial, transvenous, or direct puncture alone or their combinations were adopted, and various materials including polyvinyl alcohol particles, metal coils, cyanoacrylate agents, Onyx, and ethanol were used alone or in combination as embolic materials. A long-term stable effect to prevent hemorrhage was obtained in many reports, but the recurrence rate was high when polyvinyl alcohol particles were used, and they were not recommended except in preoperative treatment^89)^. Some papers suggested that the embolization method and materials should be selected according to the vascular architecture of AVM, possibly with the addition of curettage depending on the case^45,83)^. Conditions including mucocutaneous necrosis^33,80,84,89,95,96)^, peripheral neuropathy^95)^, bone necrosis^78)^, and infections^33,96)^ have been reported as complications of endovascular embolization, but many of them were regarded as minor complications^78,95)^. Monteiro *et al.* reviewed 50 cases treated mainly by embolization in the literature and reported that complications occurred in 50% but that most of them were minor^95)^.

###### <Conclusion>

The above discussion suggests at present that there is no indication for radiotherapy and that treatment by surgery alone cannot be recommended. As treatment, endovascular embolization including ethanol sclerotherapy alone or in combination with surgery is recommended, and, regarding surgery, the lesion may be controlled less invasively by combining it with endovascular embolization. The procedure of endovascular embolization may be transarterial, transvenous, or direct puncture, and various embolic materials are used. It may be necessary to change the embolization procedure depending on the vascular architecture of AVM, and it is recommended that an experienced endovascular operators capable of handling various techniques perform treatment in an environment where surgical backup is available.

##### 3．Evaluation of balance of benefits and risks

Maxillomandibular AVM tends to repeatedly cause possibly lethal massive hemorrhage, and its treatment by endovascular embolization alone or in combination with surgery has been reported to yield a long-term stable effect to prevent hemorrhage, which is a great benefit to patients. Complications of endovascular embolization as its risks include mucocutaneous necrosis, peripheral neuropathy, bone necrosis, and infections, but many of them are relatively mild, and serious complications that lead to massive hemorrhage or serious complications, such as severe functional impairment of deformation or death, are rare even after surgical treatment. Therefore, generally, benefits of these treatments surpass their risks, but as the incidence of complications is relatively high, balance between benefits and risks must be evaluated in individual patients.

##### 4．Patients’ values/wishes

These treatments are considered to comply with the values/wishes of many patients in that they prevent massive hemorrhage and preserve the normal development and function of the jaw.

##### 5．Cost assessment, assessment of external validity of intervention

Both endovascular embolization and surgery are covered by National Health Insurance in Japan and do not cause a large burden on medical economy. However, some embolic materials, such as Onyx, are not covered by National Health Insurance in Japan, and the validity of their use must be evaluated in planning their use.

#### Lay summary

Maxillomandibular AVM is a serious disorder that repeatedly causes possibly lethal massive hemorrhage. Endovascular embolization, by which the lesion is occluded from inside the blood vessel, is highly likely to prevent massive hemorrhage if performed alone or in combination with surgery. Although the incidence of complications of this treatment is relatively high, many of them are mild, and the treatment is recommendable in consideration of their therapeutic effects. There are a variety of methods for endovascular embolization, and they must be selected according to the disorder.

### CQ 5: What treatments are appropriate for AVM of the fingers?

Recommendation: Complete cure is difficult to achieve by embolization or sclerotherapy alone, and it is recommended to perform them for alleviation of symptoms such as pain. However, they occasionally cause finger necrosis or neuropathy. Concerning surgical resection, total resection is recommended, because partial resection is likely to result in enlargement of the lesion. Although preoperative embolization or sclerotherapy is useful, there is the risk of finger necrosis or neuropathy, and careful evaluation is needed.

Strength of recommendation: 2 (weak)

Evidence: D (very weak)

#### Process of preparation of recommendation

##### 1．Circumstances that make CQ an important clinical issue

AVM may be difficult to treat and may also differ in the therapeutic effects and complications, depending on its site such as the orbit/eyelid, tongue/oral cavity, mandible/maxilla, fingers/toes, soles, and joints. Particularly, AVM of the fingers, which are often encountered clinically, markedly affects the patient’s QOL, but its treatment is often difficult. Therefore, it is important to evaluate its treatments and their effects and complications.

##### 2．Evaluation of evidence

###### <Literature search>

As a result of systematic literature search, 93 papers (42 from PubMed, 51 from JCRM) were selected for primary screening. As a result of primary screening, 15 papers (8 from PubMed, 7 from JCRM) were selected for secondary screening, which resulted in retrieval of 12 papers (7 from PubMed, 5 from JCRM). The extracted literature was only case reports or case series studies, and their evidence level was rated as low.

###### <Evaluation>

Concerning embolization performed alone, procedures using n-butyl-2-cyanoacrylate or isobutyl cyanoacrylate have been reported, but the occurrence of skin necrosis, finger necrosis, and transient neuropathy has been reported as complications, and cases that developed re-enlargement due to the development of collateral channels, requiring resection or amputation, have also been reported^26,97)^. There is also a report that complications including necrosis occurred after ethanol embolization alone due to leakage of the drug in more than half of cases^25)^. Although there is a report that pain could be controlled at symptomatic sites by repeatedly performing isobutyl cyanoacrylate embolization for pain control rather than complete cure, but re-enlargement of the lesion may occur as a complication^98)^. Concerning AVM with a major draining vessel, satisfactory therapeutic outcome was reported to be achieved by ethanol embolization of the nidus after coil embolization of the major draining vessel^18)^.

Regarding surgical treatment, no re-enlargement is observed after total resection, and reconstruction using a flap is often performed^99-102)^. However, by total resection, hand function cannot be preserved, or necrosis may occur on the distal side, and re-enlargement is observed after partial resection or ligation of the feeding artery^103,104)^.

3D-CT angiography is useful as an examination, and, in surgical therapy, preoperative embolization is effective^100,105)^. In treating AVM, it is recommended to make assessments and review the treatment plan at multiple clinical departments, such as plastic surgery, vascular surgery, and radiology, at each time of treatment^25)^.

###### <Conclusion>

To summarize the above discussion, skin and finger necrosis, transient neuropathy, and re-enlargement have been reported as adverse events related to embolization of AVM of the fingers. For AVM with a main draining vein, coil embolization of the main draining vein, followed by ethanol embolization of the nidus, is effective. In treatment by embolization alone, adverse events are likely to occur, and sufficient precaution is necessary. Concerning surgical treatment, re-enlargement has been reported after partial resection or ligation of the feeding artery. By total resection, the risk of re-enlargement is low, but reconstruction is often necessary, and sufficient precaution is needed as there are cases in which the hand function cannot be preserved, or necrosis occurs distally. Also, preoperative embolization is effective. 3D-CT angiography is useful as an examination, and assessment and review of the treatment plan at multiple clinical departments at each time of treatment are recommended.

##### 3．Evaluation of balance of benefits and risks

Complications of embolization include skin necrosis, necrosis of the fingers, transient neuropathy, and re-enlargement. Also, complications of surgical treatment include re-enlargement associated with partial resection or ligation of the feeding artery and decline in finger functions and distal necrosis associated with total resection. These are all adverse effects (risks) to patients and may result in sequelae and death. Among them, re-enlargement due to expansion of the area occupied by AVM, which may lead to progression of Schobinger stage, is a clinically significant complication. Its frequency is high after embolization alone, partial resection, and ligation of the feeding artery but low after total resection. Performing treatments without evaluating common complications is a disadvantage (harm) to patients, and not treating AVM that requires treatment for fear of rare complications is also a disadvantage (harm) to patients. Clarification of the frequency of the occurrence of complications and identification of risk factors of complications are important (benefits) to patients, and clarification of the course expected from not treating the disorder is also important for patients (benefits). Balance of these benefits and risks must be considered in evaluating the strength of recommendation of this CQ.

##### 4．Patients’ values/wishes

Avoiding complications associated with treatment is considered to comply with the patients’ values and wishes. Treating AVM that requires treatment by procedures that frequently accompany complications does not necessarily comply with the patients’ values or wishes.

##### 5．Cost assessment, assessment of external validity of intervention

Embolization, sclerotherapy, surgery, and associated evaluation and examinations before these treatments are all covered by National Health Insurance in Japan and do not cause a large burden on medical economy.

#### Lay summary

AVM of the fingers markedly affects the QOL. Although many patients are suffering, treatment is often difficult, so we addressed the question of which treatment is safer and more effective. Embolization and surgery are performed for AVM of the fingers, but, presently, there is no treatment with clear evidence that it is the safest and most effective. Embolization alone, surgical resection of only part of AVM, and ligation of the vessel that supplies blood to AVM may result in enlargement of the lesion (re-enlargement). Although the possibility of re-enlargement is low after complete surgical resection, it may cause disability to the hand or require surgery to restore the area lost by surgery (reconstruction). Since treatment of AVM involves the risk of the occurrence of necrosis of the skin or fingers and impairment of the senses and movements of the fingers in addition to re-enlargement, it is recommended to carefully study the symptoms and condition of AVM in each patient and determine the course of treatment through sufficient discussion between the patient and the physician in charge after evaluation among physicians of different specialties.

### CQ6: What treatments are effective for painful VM?

Recommendation: Conservative treatments, such as compression, anti-inflammatory analgesic medications, oral aspirin, oral sirolimus, and low-molecular-weight heparin, are reported to be effective depending on the site, size, and symptoms of VM. Conduction anesthesia may be effective depending on the site of the lesion.

Strength of recommendation: 2 (weak)

Evidence: D (very weak)

#### Process of preparation of recommendation

##### 1．Circumstances that make CQ an important clinical issue

In VM, pain may be caused by thrombophlebitis, thrombotic occlusion, or localized intravascular coagulopathy (LIC) that develops in the lesion, and pain is a major symptom of VM. Since pain impairs the patient’s QOL, some intervention is necessary, but the evaluation of which conservative treatments as well as invasive treatments are effective is an important clinical issue.

##### 2．Evaluation of evidence

###### <Literature search>

As a result of systematic literature search, 594 papers (519 from PubMed, 75 from JCRM) were selected for primary screening. As a result of primary screening, 14 papers (12 from PubMed, 2 from JCRM) were selected for secondary screening, through which 6 papers (6 from PubMed, 0 from JCRM) were adopted. At the time of the preparation of the previous guidelines (Japanese clinical practice guidelines for vascular anomalies 2017), the references were mostly reports on invasive treatments effective for pain, and those concerning conservative treatments were few. However, there were more reports concerning conservative treatments at the time of preparation of the present guidelines.

###### <Evaluation>

While CQ7 of the previous guidelines (Japanese clinical practice guidelines for vascular anomalies 2017) was “What treatments are effective for painful venous malformations?”, it was changed to “What conservative treatments are effective for painful venous malformations?” in the present guidelines (CQ6), placing more weight on conservative treatments. In the background is the trend that non-invasive approaches have begun to be preferred for VM difficult to treat by conventional invasive procedures such as surgical resection and sclerotherapy. This view is supported by the increase in reports concerning conservative treatments as mentioned above.

Concerning conservative treatments effective for painful VM, literature search was made for reports concerning compression therapy, anti-inflammatory analgesic drugs, low-dose aspirin, low-molecular-weight heparin, sirolimus, and stellate ganglion block. The first 4 are treatments that have been conventionally adopted, and although a review article^106)^ and a case series study concerning aspirin^107)^ were extracted, their quality as evidence is not high. The last 2 are relatively new reports. Particularly, sirolimus, which is a mammalian target of rapamycin (mTOR) inhibitor, is expected to have an epoch-making effect with a non-conventional action mechanism, and a few case series studies are available^108-110)^. One paper about stellate ganglion block performed for VM^111)^ was extracted.

In the review article^106)^, compression therapy, anti-inflammatory analgesics, and low-molecular-weight heparin were mentioned, but their dosage and administration were not evaluated.

In the case series study concerning aspirin^107)^, alleviation of pain was observed in 15 (68%) of the 22 cases at a low dose (5–10 mg/kg). The evidence level is low, because the administration period was unclear, the study design was retrospective, and there was no control group.

Sirolimus, a mTOR inhibitor, has recently attracted attention, and the literature is accumulating. Regarding its dosage and administration, the drug was reported to be effective for alleviating pain by the administration started at 0.8 mg/m^2^ 2 times a day^109,110)^ or 2 mg once a day^108)^ and maintaining the blood concentration at 10–15 ng/mL^108,110)^ or 10–13 ng/mL^109)^.

The literature concerning nerve block was a case report on stellate ganglion block performed for controlling pain of upper limb VM^111)^.

###### <Conclusion>

The extracted literature was a review article, case series study, and case reports. Various treatments are reported to be effective for mitigating pain, and evidence with better quality is awaited for the future.

##### 3．Evaluation of balance of benefits and risks

Since the possibility of the therapeutic effect and adverse events differs with the treatment and the site and size of VM, it is difficult to evaluate the balance between benefits and risks.

##### 4．Patients’ values/wishes

Alleviation of pain is one of the major objectives of treatment for VM, and it obviously complies with the patients’ wishes. However, the patients’ values/wishes may change according to the severity and frequency of pain, adverse reactions to various treatments, and degree of their burdens to the patients.

##### 5．Cost assessment, assessment of external validity of intervention

Of the above treatments, compression therapy is presently not covered by National Health Insurance in Japan, and, in this respect, it is likely to cause economic burdens on patients, but no reliable cost assessment (evaluation of the economic aspect) has been made. Sirolimus was approved as a treatment covered by National Health Insurance in Japan for VM in January 2024. There have also been few reports on the assessment of external validity of intervention, and it remains insufficient.

#### Lay summary

As conservative treatments for painful VM, compression therapy, anti-inflammatory analgesics, oral aspirin, oral sirolimus, and low-molecular-weight heparin infusion have been reported to be effective according to the site, size, and symptoms of the lesion. Also, there was a case report that conduction anesthesia was effective. Since this summary is based on a review of the literature, not all treatments mentioned here are effective in all patients, and it is necessary to select treatments depending on the condition of individual patients.

### CQ7: Is laser therapy effective for VM?

Recommendation: Laser therapy using Nd:YAG laser or diode laser can be an effective choice for VM of the mucosa, tongue, lip, and glans penis, where scar formation after treatment poses not a serious problem, and small VM. Comparative evaluation of these treatments with other treatments, such as sclerotherapy and surgical resection, is recommended in each case according to the site, size, and symptoms of the lesion.

Strength of recommendation: 2 (weak)

Evidence: C (weak)

#### Process of preparation of recommendation

##### 1．Circumstances that make CQ an important clinical issue

Although VM is a benign disorder, it causes pain or functional impairment or poses problems with the appearance depending on its site. Conventionally, VM has been treated by surgical resection, but complete resection is impossible in many cases. Recently, sclerotherapy is widely performed, and favorable therapeutic outcomes have been obtained, but the lack of coverage by National Health Insurance in Japan remains a problem. Reports of laser treatment for VM are increasing, and as coagulation of oral mucosal hemangioma was newly listed in 2018 as a treatment covered by National Health Insurance in Japan in dentistry, it is important to assess and evaluate laser therapy for VM.

##### 2．Evaluation of evidence

###### <Literature search>

As a result of systematic literature search, 187 papers (150 from PubMed, 37 from JCRM) were selected for primary screening. As a result of hand search, 1 paper from PubMed was selected for primary screening. As a result of primary screening, 39 papers (35 from PubMed, 4 from JCRM) were selected for secondary screening, through which 25 papers (22 from PubMed, 3 from JCRM) were adopted. Of the adopted literature, all but 1 review article were reports of case series or case reports, and none was analytical epidemiology or comparative study.

###### <Evaluation>

Regarding the type of laser, Nd:YAG laser was used most frequently in the adopted literature, followed by sporadic reports on diode laser. Nd:YAG laser (wavelength: 1,064 nm) is advantageous for deep lesions compared with diode laser (wavelength: 808 nm) because of the longer wavelength, but, because of thermal conversion caused as light is absorbed by water contained in the skin and mucosa, heat is also generated in perivascular tissues. Also, holmium:YAG laser (wavelength: 2,100 nm), which has a wavelength even longer than that of Nd:YAG laser, has been shown to be highly effective in case series studies with 30 or more cases^112,113)^.

Concerning small VM of the mucosa, tongue, or lip, where scar formation after treatment infrequently poses problems, there have been a number of reports that lesions could be reduced in size or obliterated by Nd:YAG laser^114-122)^. In addition, there are a case report using dye laser^123)^ and a report that small VM disappeared after treatment with Er,Cr:YSGG (erbium, chromium: yttrium, scandium, gallium garnet) laser, which is used in dentistry^124)^. On the other hand, there is a report that no reduction in lesion size was observed by Nd:YAG laser treatment^125)^, and laser therapy is considered to require experience of the therapies. There are reports of the use of laser for superficial lesions and sclerotherapy for deep lesions, and satisfactory therapeutic outcomes without major complications have been reported^126-128)^.

Concerning complications, both Nd:YAG laser and diode laser may cause temporary development of purpura/swelling and ulcers, but they mostly cure early^129,130)^. However, as there is a report of pigmentation and scar formation in the facial skin^120)^, adjustments are necessary in the setting of irradiation conditions and methods.

Also, irradiation of Nd:YAG laser or diode laser by inserting a fiber into the lesion with ultrasound guidance has been reported as a treatment that can avoid damage to the important organs or nerves^130-136)^, and, considering the reports of favorable therapeutic outcomes concerning the safety and efficacy, standardization of treatment is anticipated.

###### <Conclusion>

Nd:YAG laser and diode laser are frequently used for laser therapy, but further evaluation is necessary to obtain satisfactory therapeutic outcomes by avoiding complications.

##### 3．Evaluation of balance of benefits and risks

Although risks vary with the site of the lesion, there is the possibility of scar formation and damage to important tissues and nerves. In addition, patients with indication are limited, and there are few facilities capable of conducting this therapy in Japan.

##### 4．Patients’ values/wishes

No variation is observed in the values or preferences concerning the therapeutic effects, such as improvements in size and symptoms, and the degree of consistency, is high.

##### 5．Cost assessment, assessment of external validity of intervention

Coagulation of hemangioma of the oral mucosa has been listed as a treatment covered by National Health Insurance in Japan in dentistry since 2018, but the procedure can be performed only in facilities given permission. Presently, there are problems with the cost and limitation of facilities that can perform the treatment, and further research is necessary.

##### 6．Summary

While reports of laser therapy for VM have increased, all references adopted in the preparation of the present guidelines are case series or case reports except for 1 review article, no comparative studies between laser therapy and surgery or sclerotherapy or among laser irradiators with different wavelengths have been conducted, and the treatment has not been standardized. However, there have been reports of satisfactory outcomes and minimum complications by many case series studies.

Although Nd:YAG laser and diode laser are generally recommended, the irradiation conditions and methods (lesional/intralesional irradiation) have not been standardized according to the site and size of the lesion. Further evaluation is needed for achieving satisfactory therapeutic outcomes while avoiding severe complications.

#### Lay summary

Reports on laser therapy for VM are increasing, and satisfactory outcomes have often been reported in small lesions of the mucosa, tongue, or lip, where problems with scar formation after treatment are rare. However, laser therapy for VM has not been standardized and is not widely available. It is necessary to choose the treatment by comparing it with other treatments such as surgery and sclerotherapy. While coagulation of hemangioma of the oral mucosa has been listed as a treatment covered by National Health Insurance in Japan since 2018, it can be performed only in facilities given permission. Also, it is not covered by National Health Insurance in Japan if it is applied to sites other than the oral mucosa.

### CQ8: Is sclerotherapy effective for VM?

Recommendation: Sclerotherapy for VM is effective for alleviating symptoms and reducing the size of the lesion and is recommended.

Strength of recommendation: 1 (strong): Recommended to be performed

Evidence: C (weak)

#### Process of preparation of recommendation

##### 1．Circumstances that make CQ an important clinical issue

Sclerotherapy is a major treatment for VM. Although it is less curative compared with surgical resection, it is advantageous for morphological and functional preservation. Therefore, it is important to evaluate the current evidence concerning symptomatic improvements and reduction of the lesion size that can be achieved by this treatment.

##### 2．Evaluation of evidence

###### <Literature search>

As a result of systematic literature search, 448 papers (376 from PubMed, 72 from JCRM) were selected for primary screening. As a result of hand search, 19 papers (19 from PubMed, 0 from JCRM) were selected for primary screening. As a result of primary screening, 149 papers (145 from PubMed, 4 from JCRM) were selected for secondary screening, through which 42 papers (40 from PubMed, 2 from JCRM) were adopted. The extracted literature included 4 reports of quasi-RCTs, but the randomization and blinding method were inadequate, and their quality as RCT was low. Also, the theme evaluated by all these RCTs was “comparison of sclerosing agents in sclerotherapy”, and no study made comparison with other treatments. Thus, no control group related to this CQ was established, and the contribution of these trials to the overall evidence is weak. All the other references were case reports or reports of case series, but studies with a relatively large number of cases including prospective studies are increasing, and the strength of evidence was rated as C (weak).

###### <Evaluation>

Although the evidence level is low, there are a few case series with a large number of cases and prospective studies, and a systematic review was performed concerning the reports without confusion in the evaluation of the outcome, such as symptomatic improvement and changes in lesion size, among the RCTs, case series with 50 or more cases, and prospective studies reported between 1980 and 2020. As a result, 4 quasi-RCTs, 23 case series studies, and 7 prospective studies (total: 34 reports) were reviewed. (A Table 1 summarizing the results of clinical studies of 50 or more cases is shown in the end of this CQ.)

The evaluation was made by the outcomes (O) constituting CQ set up according to PICO.

Whether there was O1: symptomatic improvement, O2: reduction of lesion size, or O3: recurrence (re-enlargement).

Improvements in symptoms were observed in 48–100%^137-155)^, and moderate or marked reduction of lesion size was observed in 16–100%^137-142,146,147,149,150,153,156-165)^. Despite the wide variation of the results according to the type of sclerosing agent and characteristics of the lesion, sclerotherapy was suggested to be useful (reports with a large number of cases are shown below). There were many reports in which the symptomatic improvement was not necessarily correlated with the reduction of lesion size. Also, many patients required multiple treatments, and the recurrence rate after symptomatic improvement was 0–18%^137,144,145,149,150,153,166,167)^. In a report with a particularly large number of cases, the outcome was complete cure in 185, marked improvement in 44, and some improvement or no change in 31 of the 260 cases^157)^. Also, the degree of reduction of lesion size was very good in 104 and good in 10 of the 120 cases^161)^.

The sclerosing agents used ranged widely and included ethanol, polidocanol, sodium tetradecyl sulfate, and bleomycin. The methods of treatment and assessment of their effectiveness also varied widely. Attention to this fact is necessary in interpreting this systematic review.

O4: Serious treatment-related complications

Regarding complications, those corresponding to grade 3 or severer complications according to the Common Terminology Criteria for Adverse Events (CTCAE) were observed in 0–13% of the patients^137-142,144-147,149-160,162,166-169)^. They ranged widely from mild ones, such as transient neuropathies^143,145,152,153,159)^ and local inflammation^149)^, to serious ones such as muscle disorders^169)^, skin/bone necrosis^145,149,153,158,160,169,170)^, deep vein thrombosis/pulmonary embolism^167,171,172)^, compartment syndrome^140,168)^, paradoxical embolism in a case of VM complicated by patent foramen ovale^168)^, blindness after treatment of head and neck lesions^173)^, orbital compartment syndrome^168)^, and transient spinal cord ischemia after treatment of a paravertebral lesion^139)^. As particularly serious complications, severe cardiopulmonary complications, such as cardiac arrest in sclerotherapy using polidocanol and ethanol^174-176)^ and acute pulmonary disorder in sclerotherapy using bleomycin^177)^, were reported.

###### <Conclusion>

By sclerotherapy for VM, symptomatic improvement was observed in 48–100%, and moderate or greater decrease in lesion size was observed in 16–100%, and the treatment is useful. Symptomatic improvement was not necessarily correlated with reduction in lesion size. Many patients required treatment multiple times, and the recurrence rate after symptomatic improvement was 0–18%.

The incidence of complications corresponding to CTCAE grade 3 or severer was 0–13%, and they included muscle disorders and skin/bone necrosis, deep vein thrombosis/pulmonary embolism, compartment syndrome, paradoxical embolism in cases complicated by patent foramen ovale, blindness after treatment of head and neck lesions and orbital compartment syndrome, and transient spinal cord ischemia after treatment of paravertebral lesions. As particularly serious complications, cardiac arrest due to polidocanol and ethanol, and acute pulmonary disorder due to bleomycin have been reported.

##### 3．Evaluation of balance of benefits and risks

Sclerotherapy is generally effective for VM (benefits), but the possibility of serious complications (risks) is not nil. However, from the incidence of serious complications (0–13%), the balance between benefits and risks is not significantly poor. The treatment should be performed with attention to the site of treatment and the dose of the drug used.

##### 4．Patients’ values/wishes

Some patients’ associations are engaging in signature-collecting campaigns for National Health Insurance coverage over sclerotherapy for VM, and there seems to be a certain level of social demand for this in Japan.

##### 5．Cost assessment, assessment of external validity of intervention

Despite expectation of some symptomatic improvement, since reports considerably vary concerning whether reduction of lesion size can be obtained or not, detailed explanations are necessary for patients undergoing the treatment for aesthetic purposes. Regarding the cost, there has been a study that evaluated the cost-effectiveness of sclerotherapy for painful VM by specialized medical economic assessment^178)^, which supported the cost-effectiveness of the treatment for improving QOL in symptomatic patients.

#### Lay summary

Symptomatic improvement and reduction of lesion size were achieved in many reports that evaluated the effectiveness of sclerotherapy, and the treatment is effective. However, severe complications were observed at a frequency of up to 13%, and precautions are needed about the site of treatment and dose of the drug used. Recently, sclerotherapy has also begun to be performed widely in Japan, but it remains not covered by National Health Insurance in Japan as of 2021.

### CQ9: Are clotting abnormalities due to VM an indication for radiotherapy?

Recommendation: VM and vascular tumors are suspected to be mixed in many reports, and the effectiveness of treatment cannot be evaluated. Also, as development of malignant tumors, growth disorders, and functional impairment have been reported as late complications, radiotherapy should not be performed without careful evaluation.

Strength of recommendation: 1 (strong): Recommended not to be performed

Evidence: D (very weak)

#### Process of preparation of recommendation

##### 1．Circumstances that make CQ an important clinical issue

Radiotherapy used to be performed as a treatment for clotting disorder due to VM. However, while there was the risk of the development of malignant tumors, such as carcinoma and sarcoma, visual impairment, and short limbs caused by radiotherapy, no clear therapeutic effect has been reported. This CQ is of clinical importance in a negative sense for the prevention of unreasonable use of radiotherapy as a treatment for clotting disorder due to VM.

##### 2．Evaluation of evidence

###### <Literature search>

As a result of primary screening, 14 papers from PubMed and 3 papers from JCRM were extracted. However, secondary screening showed that many cases of infantile hemangioma and hepatic hemangioma were included. After their exclusion, only 1 paper from PubMed (case series study) remained to be evaluated. By hand search, 9 papers adopted in the 2017 edition were added. In the newly adopted paper and the papers added by hand search, the distinction between vascular malformation and angioma was unclear, and there was no report on radiotherapy for VM alone. These documents were reviewed as a whole. The literature reviewed was the same as that adopted by screening for the same CQ in the 2017 edition, and the evidence level of the literature concerning CQ9 was rated as D (very weak).

###### <Evaluation>

In the paper adopted by the present systematic literature search^179)^, the subjects were 13 cases of symptomatic hemangioma (11 of them were pathologically diagnosed as cavernous hemangioma, but hemangioma and vascular malformation were not distinguished in this old paper, and the lesions were probably mixed). Radiotherapy was performed in the 13 cases at 6.25-40 Gy. The lesions consisted of 5 in the limbs, 2 in the face, 3 in the vertebral body, 1 in the hypophysial fossa, 1 in the sacrum, and 1 in the bladder, and the organs to be excluded in the present guidelines were included. Of these lesions, 2 (1 in the lower limb and 1 in the face) exhibited the Kasabach–Merritt phenomenon, and clotting disorder (evaluated according to the platelet count and fibrinogen level) was resolved after treatment. However, these patients were 3-year-old and 5-month-old children and, actually, may not have been cases of VM. In the cases with lesions in the limbs or face, complete response (CR) regarding reduction of lesion size was observed in 2, partial response (PR) was observed in 4, and no response was observed in 1; CR regarding symptomatic improvement was observed in 4, PR was observed in 1, and no response was observed in 2. A serious treatment-related complication was observed in 1 (14 Gy/8 split doses), who developed unilateral visual impairment. According to the papers extracted by hand search, the development of malignant tumors, such as breast cancer^180)^, adenocarcinoma^181)^, and angiosarcoma^182)^, visual impairment mentioned above^179)^, shortening of the lower limb, and restricted range of joint motion^183)^ as late complications of radiotherapy performed in infancy for infantile hemangioma were discussed as problems.

According to Caldwell *et al*.^182)^, late complications of radiotherapy for hemangiomas in infancy include bruise and Stewart-Treves syndrome after the patients reach adulthood. Angiosarcoma is also observed. They reported that the median survival period was 24 months, and the 5-year survival rate was approximately 10%, in those who developed angiosarcoma.

While there have been reports that radiotherapy was performed for the treatment of vascular tumors and vascular malformations, it is difficult to judge whether the treatment was performed by distinguishing the disorders. According to many reports^179,183-186)^, radiotherapy has been performed to treat Kasabach-Merritt phenomenon. However, while there is no mention of Kasabach-Merritt phenomenon, there is a report^187)^ of five cases in which giant hemangiomas accompanied by clotting disorders, thrombocytopenia, heart failure and bleeding were controlled by multidisciplinary treatment including radiotherapy. However, vascular tumors that cause Kasabach-Merritt phenomenon are considered to be kaposiform hemangioendothelioma or tufted angioma rather than infantile hemangioma^188)^. Thus, because VM and infantile hemangiomas are considered to be mixed with vascular tumors in these reports, these reports do not support implementation of radiotherapy for VM or infantile hemangiomas.

###### <Conclusion>

As observed above, the diagnoses were not confirmed in the reports that have suggested the effectiveness of radiotherapy, and its indications have not been specified. In addition, there have been a considerable number of reports of late complications due to radiotherapy. Thus, radiotherapy should not be performed without careful evaluation.

##### 3．Evaluation of balance of benefits and risks

In preparing the recommendation concerning this CQ, importance was attached to outcome (O) 1 (benefits): the treatment is effective for clotting abnormality with improvements in clotting abnormality, and O2 (risks): there are malignant tumors, growth impairment, and functional impairment as late complications, set up by evaluating PICO. No paper reported O1 by clearly distinguishing infantile hemangioma and VM. However, concerning O2, late complications were reported in multiple papers. Although the cases are small in number, each late complication should not be disregarded.

##### 4．Patients’ values/wishes

Radiotherapy involves exposure and requires time and cost. Since late complications have been reported despite uncertainty of clinical effects, patients are unlikely to wish this treatment.

##### 5．Cost assessment, assessment of external validity of intervention

Radiotherapy generates the costs of hospital visits, inpatient care, and treatment. The assessment of late complications also involves cost.

#### Lay summary

The effectiveness of radiotherapy for clotting abnormality due to VM remains questionable, but the occurrence of malignant tumors, growth disorder, and functional impairment have been reported as complications that develop long after treatment. Therefore, it is recommended to avoid radiotherapy as a treatment for clotting abnormalities.

### CQ10: Is the assessment of clotting abnormality useful for the follow-up of VM?

Recommendation: The assessment of clotting abnormality, particularly the assessment according to the D-dimer level, is proposed for the follow-up of VM. In addition, blood cell counts, fibrinogen, prothrombin time (PT), and activated partial thromboplastin time (APTT) are also examined for the assessment.

Strength of recommendation: 2 (weak)

Evidence: B (moderate)

#### Process of preparation of recommendation

##### 1．Circumstances that make CQ an important clinical issue

Localized intravascular coagulopathy (LIC) in VM is a disorder that may cause serious symptoms, such as pain, and bleeding tendency, and disseminated intravascular coagulation (DIC) due to stagnation of the blood flow in VM and consequent local activation of the blood coagulation system.

Appropriate assessment of the activity of LIC may lead to better and more prompt treatment. Among the assessment methods, the role played by examination of clotting abnormalities and assessment indices are evaluate.

##### 2．Evaluation of evidence

###### <Literature search>

As a result of systematic literature search, 226 papers (168 from PubMed, 58 from JCRM) were selected for primary screening. As a result of primary screening, 15 papers (15 from PubMed, 0 from JCRM) were selected for secondary screening, through which 8 references (8 from PubMed, 0 from JCRM) were adopted. They consisted of 4 prospective studies, 2 case-control studies, and 1 each of cross-sectional study and guidelines.

###### <Evaluation>

According to the report by Dompmartin *et al*., of the 140 VM patients, the D-dimer level was elevated (>0.5 µg/mL) in 42%, of whom it was ≥1.0 µg/mL in 61%. As a result of multivariate analysis, a surface area of ≥10 cm^2^ (odds ratio [OR]: 2.82) and palpation of phlebolith (OR: 3.16) were suggested as risk factors for LIC^189)^.

Dompmartin *et al.* also analyzed 195 patients diagnosed with VM and 85 non-VM outpatients and reported that the sensitivity and specificity of an elevation of D-dimer (>0.5 µg/mL) were 42.6% [95% confidence interval (CI): 35.6–49.5%] and 96.5% (95% CI: 92.5–100%), respectively, indicating D-dimer as a useful prognostic factor for VM^190)^.

According to the report by Mazoyer *et al*., of the 118 VM patients, D-dimer was elevated (>0.5 µg/mL) in 58%, of whom 96% had VM involving muscles. Moreover, the elevation of D-dimer level was ≥1.0 µg/mL in 39%, of whom VM involved muscles in 93%. Also, the elevation of D-dimer was correlated with the severity score. A decrease in Factor VIII-von Willebrand factor complex (<60%) was observed in 27%, which was 10-fold higher compared with the control population. Prolongation of PT or APTT was observed in patients with severe VM^191)^.

According to the report by van Es *et al.*, D-dimer was elevated (>0.5 µg/mL) in 51% of the 67 VM patients. No significant difference was observed in the values of D-dimer, PT, APTT, P-selectin, or von Willebrand factor according to the presence or absence of intralesional blood clots. The size of VM was positively correlated with the D-dimer level^192)^.

Some papers evaluated changes in blood clotting factors associated with therapeutic intervention. Mason *et al*., studied 20 cases treated by absolute alcohol embolization and 11 cases treated by sodium tetradecyl sulphate sclerotherapy and observed that the treatments were positively correlated with decreases in the platelet count and fibrinogen, PT prolongation, and an increase in D-dimer^193)^.

Also, according to the report of absolute ethanol embolization therapy performed in 18 children by Leung *et al.*, the D-dimer level was elevated (>0.5 µg/mL) preoperatively in 44% of the patients. D-dimer increased to 1.5 times or more compared with the preoperative level within 2 weeks after the treatment in 67% of the patients, of whom 92% showed a peak value on the first postoperative day^156)^.

In addition, Rodríguez-Mañero *et al*. showed that the pulmonary artery systolic pressure was significantly higher (P < 0.001), and D-dimer and von Willebrand factor were significantly increased (P < 0.001, P = 0.01, respectively) in 32 patients with VM involving ≥15% of the body surface area compared with a healthy population. The D-dimer and von Willebrand factor levels showed positive correlations with the pulmonary artery systolic pressure (P < 0.001, P = 0.001, respectively)^194)^.

The 2013 revised edition of the guidelines of the International Union of Phlebology (IUP) recommended screening of patients with extensive VM or high-risk lesions by examination of the blood cell counts, D-dimer, fibrinogen, PT, APTT, and thrombotic diathesis^195)^.

###### <Conclusion>

Since LIC can be a risk that causes serious complications, such as DIC, it is important to appropriately evaluate the disease activity, and the assessment using blood clotting factors has been reported to be useful. Particularly, an elevation of the D-dimer level is a characteristic finding, and it is reportedly observed in a considerable percentage of VM patients. A large surface area (>10 cm^2^) and the presence of palpable phleboliths are risk factors, and D-dimer is more likely to increase in VM that affects muscles. The D-dimer level is correlated with the severity score and is considered to be an index useful for clinical judgments. In addition, as prolongation of PT and APTT and decreases in fibrinogen and platelets are observed with increases in the severity of the condition, examination also of these items is recommended.

There have also been reports of changes in the laboratory results concerning blood clotting abnormality associated with embolization/sclerotherapy, and their evaluation before treatment contributes to anticipation of the risk of complications and precautionary measures such as anticoagulant administration. On the other hand, since D-dimer increases non-specifically immediately after surgery or sclerotherapy, it is not useful for the assessment of postoperative thrombotic complications.

On the basis of the above discussion, blood tests are useful for the follow-up of VM, and D-dimer, blood cell counts including platelets and hemoglobin, fibrinogen, PT, and APTT are proposed as the items to be examined.

##### 3．Evaluation of balance of benefits and risks

Regarding benefits related to this CQ, since the disease activity of LIC can be clarified, and serious complications, such as DIC and the tendency of exacerbation, can be evaluated, in VM patients by periodic follow-up with examination of blood clotting abnormalities, early therapeutic intervention and risk assessment are made possible. Regarding risks, while tests of blood clotting abnormalities do not directly exacerbate the disease, blood drawing may cause pain. Balance between these benefits and risks must be considered in evaluating the strength of recommendation concerning this CQ.

##### 4．Patients’ values/wishes

The assessment of clotting abnormalities is considered to conform to the values/wishes of many patients in that it contributes to the prediction of progression of the disease and prevention of serious conditions. However, how the burden of periodic blood drawing is accepted varies with each patient’s values/wishes.

##### 5．Cost assessment, assessment of external validity of intervention

All tests of blood clotting abnormalities proposed in this CQ are common examinations covered by National Health Insurance in Japan and are not considered to cause a large burden on medical economy.

#### Lay summary

VM may cause a condition called LIC. This condition may cause pain and bleeding due to stagnation of the blood flow in VM and may progress to a serious condition called DIC. It has been reported that, among blood test items, the D-dimer level is particularly useful for the assessment of LIC and that blood cell counts including platelets and hemoglobin, fibrinogen, PT, and APTT are also useful as references. Periodic implementation of these blood tests is expected to make appropriate monitoring of the disease activity of VM and prompt responses possible.

### CQ11: Is there a difference in the effectiveness of dye laser treatment for CM according to the site?

Recommendation: Dye laser treatment for CM is likely to be more effective in the face and neck region compared with other sites, and it is more likely to cause complications such as pigmentation in the limbs.

Strength of recommendation: 2 (weak)

Evidence: C (weak)

#### Process of preparation of recommendation

##### 1．Circumstances that make CQ an important clinical issue

Presently, dye lasers are the first-line treatment for CM and is considered to have few complications. However, cases in which complete resolution cannot be achieved even by repeated treatments and cases in which redarkening of the stain occurs with time after treatment have been reported, and it is considered important to evaluate possible differences in the effectiveness of treatment depending on the anatomical site of the lesion.

##### 2．Evaluation of evidence

###### <Literature search>

As a result of a systematic literature search, 238 papers (184 from PubMed, 54 from JCRM) were subjected to primary screening. As a result of a hand search, 3 papers (3 from PubMed, 0 from JCRM) were subjected to primary screening. As a results of primary screening, 61 papers (46 from PubMed, 15 from JCRM) were subjected to secondary screening, through which 41 papers (29 from PubMed, 12 from JCRM) were adopted.

Although the descriptions of the target disorder were not standardized, many reports were retrieved by search of the Japanese literature using capillary malformation, hemangioma simplex, and port-wine hemangioma and the English literature using port-wine stain as search words. They included a few studies that are allegedly RCTs, but, in fact, their contents were considered short of RCTs. Therefore, since none of the case series studies with a relatively large number of cases adopted through the secondary screening was a RCT, the evidence level of the literature as a whole was rated as C (weak).

###### <Evaluation>

Concerning the effects of dye laser treatment for CM, most of the reports were about the effects on hemangioma simplex or port-wine hemangioma in Japan and port-wine stain abroad. There have been a few papers^196-211)^ that evaluated the therapeutic results of dye laser treatment according to the site in a small to relatively large number of patients. The laser equipment used varied from early dye lasers to pulsed-dye lasers with variable pulse duration and a cooling system, and reports limited to variable-pulse-duration pulsed dye lasers with a cooling system, which is widely used today, were few. The outcome of dye laser treatment is known to be affected by various factors. Therefore, many of the studies pursued originality and were designed to compare positive and negative aspects of the outcome by focusing on various factors considered to affect the outcome of CM after laser treatment including the skin type, laser type, irradiation method, method of anesthesia, cooling method during irradiation, care after irradiation, diameter of the target blood vessels, depth of the vessels from the skin surface, and blood flow velocity in the blood vessels. Therefore, case series studies that discussed the outcomes after laser treatment in detail by connecting a few of these factors with the anatomical site of the lesion were also few. As observed above, because of the marked differences among the studies, it was difficult to quantitatively integrate them and evaluate them as a whole. However, despite the absence of RCTs, there have been not a few studies of relatively many cases reporting that the effects of dye laser treatment for CM vary with the site, and the outcomes did not appear to differ widely. Therefore, the adopted literature is considered to lead the recommendation statement and comments about the CQ a certain direction.

According to many reports^196-211)^, the response rate is higher in the face and neck region than in the trunk and limbs. In the face, it has been found that the response rate is higher in the palpebral, forehead and temporal, and lateral buccal regions but is significantly lower in the area of the second branch of the trigeminal nerve (dermatome V2), and that the number of irradiations tends to increase in the midline region, frequently resulting in persistence of redness^212-214)^. Regarding the response rate to laser treatment in the lower limbs, there is a report that the response rate was generally low with no significant differences according to the site^215)^ and a report that the treatment was more effective in proximal than distal lesions^216)^. There is also a report on a small number of cases, that, in the foot, pain is stronger, and the response rate is lower, but the patient satisfaction is relatively high, compared with the face^217)^. Factors that cause differences in the therapeutic effects depending on the site of the lesion are considered to include the depth of the site of CM, diameter of the vessel, hemodynamics, and skin thickness^218-223)^. Dye lasers are considered to be more effective for lesions in shallow areas of the skin and less effective in lesions in deep areas^224-227)^. It has been reported that, in the face, CM tends to be located in shallower areas of the skin in lateral regions than in the central region^214,221,228)^ and that, in the lower limbs, CM tends to be located in shallower areas of the skin, and the skin tends to be thinner, in proximal than distal regions^216)^.

The incidence of complications of dye lasers (blister formation, depigmentation, pigmentation, scar formation) is reported to be low, being 1.7% in adults, 0.6% in children, and approximately 1.4% in all patients even when all sites of the body are included, and no significant differences have been reported in the age at the beginning of treatment, Fitzpatrick skin type^229)^, site, number of treatments or irradiation energy between those who developed complications and those who did not^230)^. There are also reports that complications, such as pigmentation, depigmentation, and atrophic scars, tend to be observe more frequently in the lower limbs^217,231)^.

In addition, there are several reports that Nd:YAG lasers alone or in combination with dye lasers were effective for the treatment of CM^232-236)^, and it is considered an option for patients who respond poorly to dye lasers. Further accumulation of cases is awaited.

###### <Conclusion>

Because of the absence of reports with the same study design and high similarly of PICO items, we did not perform meta-analysis by combining data of several studies. Since most of the papers were about case series studies, the evidence level of the literature as a whole is judged to be low.

##### 3．Evaluation of balance of benefits and risks

While there are studies that suggest differences in benefits according to the site of the lesion, studies that approached whether there are differences in risks of the outcome were few. However, dye laser treatment is presently accepted widely as a treatment for CM and recognized as effective. The incidence of complications (e.g., blister formation, depigmentation, pigmentation, scar formation) is low, and serious complications are practically nil. Therefore, even treatment of lesions at sites not expected to respond well is not necessarily excluded.

##### 4．Patients’ values/wishes

Although dye laser treatment involves burdens, such as pain during irradiation and purpura formation, it is an effective treatment with a low incidence of complications and is considered to conform to the patient’s values and wishes.

##### 5．Cost assessment, assessment of external validity of intervention

Dye laser treatment for CM is covered by National Health Insurance in Japan and does not cause a large burden to the medical economy. However, it should be remembered that its effectiveness varies with the site and that the number of treatments may increase.

#### Lay summary

Dye laser treatment is currently considered the most effective treatment for CM with few serious complications. However, its effects vary with the site of the lesion. It is more effective in the face and neck region than in other regions and may be more likely to cause complications such as pigmentation in the limbs.

### CQ12: Does CM recur after dye laser treatment?

Recommendation: Although the effectiveness of dye laser treatment for CM is established, the recurrence rate may increase with time after treatment.

Strength of recommendation: 2 (weak)

Evidence: C (weak)

#### Process of preparation of recommendation

##### 1．Circumstances that make CQ an important clinical issue

Dye laser treatment is performed as a standard treatment for CM. Since this treatment requires multiple irradiations at fixed intervals, it takes a long time and is often resumed at an appropriate timing after temporary cessation. It has also been reported that the color of CM darkens again during the course of treatment. Whether this is the recurrence associated with angiogenesis of residual CM or thickened changes in the vascular wall of the remaining part of CM is unclear. Because of this background, it is considered important to evaluate whether CM may recur after dye laser treatment.

##### 2．Evaluation of evidence

###### <Literature search>

As a result of a systematic literature search, 221 papers (203 from PubMed, 18 from JCRM) were subjected to primary screening. As a result of primary screening, 26 papers (23 from PubMed, 3 from JCRM) were subjected to secondary screening, through which 12 papers (9 from PubMed, 3 from JCRM) were adopted. These papers consist of 3 on RCTs, 1 on a non-RCT, 5 on retrospective studies, and 4 explanatory/review articles, but all papers directly related to the recurrence of CM after dye layer treatment are about retrospective studies, and the evidence level is low.

###### <Evaluation>

Concerning papers that referred to “whether CM recurs after dye laser treatment”, there are 4 retrospective studies^237-239)^ after treatment by pulsed-dye laser (wavelength, 585 nm) with a cooling system, and the recurrence rate was 15.9–35%. There is also a report that the recurrence rate increased with time after treatment and was 3.1% after 1 year, 20.8% after 2 years, 40% after 3 years and 50% after 4 years^237)^. Review articles concerning Japanese patients also reported that the recurrence is possible in the short or long run after pulsed-dye laser treatment for CM^240-242)^. Therefore, it is necessary to treat CM with the recurrence after dye laser treatment in mind.

At present, CM is considered to recur after laser treatment with the development of new dilated vessels, regeneration of dilated vessels damaged by treatment, regrowth of residual dilated vessels, etc., and, as the time of their possible occurrence is also involved, discussion with clear-cut distinctions is difficult. Although there is a report that the genes that are affected by dye laser treatment shortly after treatment could be identified^243)^, their relationships with the recurrence are unclear.

Concerning the prevention of recurrence, there is a report^244)^ that the recurrence-free period was long in the patients treated within 6 months after birth with a variable-pulse pulsed-dye laser with a cooling system (wavelength, 595 nm), which is widely used today. In addition, there have been RCTs using rapamycin, which inhibits angiogenesis after laser treatment^245)^, or imiquimod^246,247)^, and these treatments are considered effective for the prevention of recurrence. However, there is also the opinion that combination therapies using rapamycin and imiquimod are effective in the short term but have not shown long-term effects^248)^. A large-scale investigation is necessary to evaluate the effectiveness of variable-pulse pulsed-dye laser treatment with a cooling system (wavelength, 595 nm) for the prevention of recurrence, and careful evaluation including the safety is necessary concerning combination therapies of dye laser treatment and drugs.

###### <Conclusion>

Because of the absence of papers with the same study design and similar PICO items, meta-analysis by combining data of several studies could not be performed. Since there were only case series studies and review articles concerning the recurrence of CM after pulsed dye laser treatment, the evidence level of the literature as a whole is inevitably judged to be low.

##### 3．Evaluation of balance of benefits and risks

Pulsed dye laser treatment is widely adopted for CM, and the incidence of its complications is reportedly low. Therefore, this treatment should not be rejected because of the possibility of recurrence.

##### 4．Patients’ values/wishes

The expectations of patients for pulsed dye laser treatment against CM are high, and it is considered to conform well to their wishes with little variation in the values or preferences in the assessment of its effectiveness according to the lesion size or symptomatic alleviation.

##### 5．Cost assessment, assessment of external validity of intervention

Pulsed-dye laser treatment for CM is covered by National Health Insurance in Japan. Treatment for CM requires multiple irradiations and a long treatment period, and the possibility of recurrence cannot be excluded. Therefore, whether the benefit of this treatment matches its cost must be evaluated carefully.

##### 6．Summary

Pulsed dye laser treatment (wavelength: 585 nm, pulse duration: 0.45 msec) with a cooling device for CM should be performed with the knowledge that the recurrence rate increases with time after treatment. Long-term large-scale studies and clinical studies of drugs including their safety and efficacy are awaited for the evaluation of how effective variable-pulse-duration pulsed-dye laser treatment (wavelength: 595 nm) with a cooling device, which has recently begun to be used, or its combination with anti-angiogenic drugs is for the prevention of recurrence.

#### Lay summary

Although dye laser treatment is performed as a standard treatment for CM, retreatment is often needed because of exacerbation of redness after initial treatment. We, therefore, evaluated the recurrence after treatment. At present, we know that there is the possibility of recurrence of redness in both short and long terms after dye laser treatment of CM, but its cause is unclear. For the prevention of recurrence, studies of new dye lasers and its combinations with drugs are under way, but there is as yet no treatment with established safety or efficacy. Therefore, in undergoing dye laser treatment for CM, it is necessary to plan the treatment with the possibility of recurrence in mind.

### CQ13: Is dye laser treatment for CM more effective as it is initiated at a younger age?

Recommendation: Laser therapy before the age of 1 year may be effective, and the earliest possible initiation of treatment is recommended as an option.

Strength of recommendation: 2 (weak)

Evidence: D (very weak)

#### Process of preparation of recommendation

##### 1．Circumstances that make CQ an important clinical issue

Concerning the timing of treatment for CM, there is the opinion that early initiation of treatment is recommended because, in young children, the skin is thinner, so the depth of penetration is larger, the vascular wall is also immature, cure after laser treatment is better, pigmentation is less and the irradiation area is small, so the treatment efficiency is higher.

##### 2．Evaluation of evidence

###### <Literature search>

As a result of systematic literature search, 51 papers (29 from PubMed, 22 from JCRM) were subjected to primary screening. As a result of primary screening, 7 papers (6 from PubMed, 1 from JCRM) were subjected to secondary screening, through which 5 papers (4 from PubMed, 1 from JCRM) were adopted. As mentioned below, the extracted literature included 3 papers on prospective studies.

###### <Evaluation>

Concerning reports that the therapeutic effects differed with the age of initiation of treatment, Oguri *et al*.^203)^ performed a non-RCT by dividing children into those aged 0–12 months, 13–24 months and 25–36 months, and observed significant differences in the response rate by combining “markedly effective” and “effective” among the groups. They also compared the response rate according to the age in months at the beginning of treatment in the 0-year-old group and reported that the response rate was higher as the treatment was initiated earlier. Furthermore, Nguyen *et al*.^249)^ divided their patients into those aged less than 1 year, those aged 1-6 years and those aged 6 or more years, and investigated the correlation between treatment response and age. They reported that those aged less than 1 year and lesions with a size of less than 20 cm2 located in the center of the face showed the best treatment responses.

Among reports suggesting no differences in the therapeutic effects according to the age at the beginning of treatment, van der Horst *et al*.^250)^ prospectively studied 100 patients with untreated CM of the head and neck region and concluded from the results of colorimetry and clinical evaluation that there were no significant differences in the therapeutic effects of pulsed-dye laser treatment among the 4 groups in which the treatment was started at the age of 0–5, 6–11, 12–17 and 18–31 years. In the retrospective study of Katugampola *et al*.^206)^, also, comparison of the 4 groups in which treatment was started at the age of 0–5, 6–12, 13–50 and 50 years or more showed no significant differences in the therapeutic effect. Moreover, Sadeghinia *et al*. also reported by a prospective study of pulsed-dye laser treatment for CM of the head and neck region in children aged 1–13 years that no differences were observed in the therapeutic effects according to age, sex, skin type, color, or size^251)^.

###### <Conclusion>

Because the papers had different conclusions, their evidence level was considered to be low when they were integrated. Comparison of papers that supported and those that did not support the usefulness of early laser treatment suggests that laser treatment is more effective when it is started at the age of less than 1 year. In addition, if the lesion is elevated and thickened with time, the condition becomes less advantageous for treatment, and the decline in the effectiveness of laser treatment is also clear from experience. In consideration of the “benefit” of early laser treatment and the “risk” of the increase in adverse events due to the vulnerability of the infant skin, the strength of recommendation was rated as 2D based on the consensus of this guideline preparation committee.

##### 3．Evaluation of balance of benefits and risks

Although an increase in adverse events due to skin vulnerability is considered a risk, its evidence is not clear, and the benefit is judged to be greater.

##### 4．Patients’ values/wishes

No literature discussing the diversity or uncertainty of the patients’ values or wishes was found. However, the disorder is likely to pose cosmetic problems, and early treatment is often requested.

##### 5．Cost assessment, assessment of external validity of intervention

Since dye laser treatment for CM is covered by National Health Insurance in Japan, it does not cause a large cost-wise burden to patients.

#### Lay summary

Since both physicians and patients are often concerned over the timing of laser treatment for CM in clinical practice, it was evaluated as a CQ. In the 8 papers extracted, the evaluation of early treatment varied, but laser treatment was suggested to be more effective when it is applied at the age of less than 1 year. Initiation of treatment as early as possible is proposed as an option based on a consensus of this guideline preparation committee, because laser treatment is likely to be more effective when it is performed at the age of less than 1 year in consideration also of the decline in its effectiveness, which is clear from experience, if the lesion is elevated and thickened with age.

### CQ14: Are there lasers or phototherapy effective for CM other than dye laser?

Recommendation: Lasers other than dye lasers or phototherapy may alleviate the stain of CM. However, as there is no evidence that they are more effective than dye lasers other than alexandrite lasers and YAG lasers in thickened CM, they cannot be first-line treatments. There is room for their tentative use in cases resistant to dye lasers.

Strength of recommendation: 2 (weak)

Evidence: D (very weak)

#### Process of preparation of recommendation

##### 1．Circumstances that make CQ an important clinical issue

Dye lasers are used for the treatment of CM. It is applied at a wavelength of 585 nm or 595 nm and a pulse duration of 0.45–40 millisecond (ms). It is effective for superficial lesions but may not be effective for deep vascular lesions. Reports of lasers other than dye lasers include those of the 488 nm argon laser, 532 nm Nd:YAG laser, 577 nm diode laser, 755 nm alexandrite laser, 1,064 nm Nd:YAG laser, and 10,600 nm CO_2_ laser. Since argon lasers and CO_2_ lasers, which are continuous-wave lasers, frequently cause scars, other pulsed lasers are mainly used for the treatment. The depth of penetration increases with the wavelength. In phototherapy, incoherent light with a wavelength of 515–1,200 nm is applied with a flash lamp unlike a single-wavelength laser irradiator. Similarly to laser, the depth of penetration increases with the wavelength.

##### 2．Evaluation of evidence

###### <Literature search>

As a result of a systematic literature search, 352 papers (166 from PubMed, 186 from JCRM) were subjected to primary screening. As a result of a hand search, 6 papers from PubMed were subjected to primary screening. As a result of primary screening, 65 papers (47 from PubMed, 18 from JCRM) were subjected to secondary screening, through which 31 papers (28 from PubMed, 3 from JCRM) were adopted. Because of limitations of the study design, such as retrospectiveness, lack of blinding, and lack of randomization, the evidence level of these references was rated as low.

###### <Evaluation>

####### I．Lasers other than dye lasers (532 nm Nd:YAG laser)^234,252-255)^

In 2003, Lorenz *et al*.^254)^ treated 43 patients previously treated with 585 nm dye lasers with 532 nm Nd:YAG lasers and 585 nm and 595 nm dye lasers and compared the results. Further improvements were observed in 5 patients by 595 nm dye laser treatment and in 6 patients by 532 nm Nd:YAG laser treatment. No adverse effects, such as long-term abnormal pigmentation or scar formation, were noted. The Fitzpatrick skin type was I–IV. In 2004, Woo *et al*.^255)^ compared the effectiveness of 532 nm Nd:YAG laser treatment and 585 nm dye laser treatment in 22 cases of CM but reported no significant difference. They reported that 75–100% improvements were observed in 27% of the patients after 10 irradiations with a 532 nm Nd:YAG laser. As adverse effects, they reported temporary itching and reddening but no scar formation. The skin type was I–IV. In 2016, Al-Dhalimi *et al*.^234)^ compared the effectiveness of 532 nm Nd:YAG laser treatment and 1,064 nm Nd:YAG laser treatment in 14 cases of CM and reported that 532 nm Nd:YAG laser treatment was significantly more effective than 1,064 nm Nd:YAG laser treatment. Pigmentation was observed in 1, but no depigmentation or scar formation was noted. The skin type was III (21.4%)–IV (78.6%).

####### II．Lasers other than dye lasers (577 nm diode laser)^256)^

In 2019, Mohamed *et al*.^256)^ applied a 577 nm diode laser 10 times to 37 cases of CM and reported that 75–100% improvements were observed in 27%. Adverse effects were temporary itching and reddening, and no scar formation was observed. The skin type was II–IV.

####### III．Lasers other than dye lasers (755 nm alexandrite lasers)^257-260)^

In 2008, Li *et al*.^259)^ compared the effectiveness of 595 nm dye lasers and 755 nm alexandrite lasers in 11 cases of CM. While both treatments were effective, and no significant differences were observed between them, 755 nm alexandrite lasers were more effective in thickened CM. As adverse effects, pigmentation was noted in 2 cases treated with the 595 nm dye laser and 3 cases treated by the 755 nm alexandrite laser, and depigmentation was observed in 1 case treated by then 755 nm alexandrite laser but none of those treated by the 595 nm dye laser. The skin type was II–IV. In 2008, McGill *et al*.^260)^ compared the effectiveness of the 532 nm Nd:YAG laser, 585 nm dye laser, 755 nm alexandrite laser, 1,064 nm Nd:YAG laser, and phototherapy in 18 patients previously treated by dye lasers. CM disappeared in 6 patients after phototherapy and in 10 patients after 755 nm alexandrite laser treatment, and, while the latter was the most effective, pigmentation and scar formation were observed in 4 patients. The skin type was I–II. In 2017, Carsen *et al*.^257)^ compared the results of treatment by the 755 nm alexandrite laser at pulse durations of 3 ms, 5 ms, and 10 ms in 16 CM patients. The therapeutic effects were significantly higher, and complications were fewer, at the pulse width of 3 ms. The skin type was I–III.

####### IV．Lasers other than dye lasers (1,064 nm Nd:YAG laser)^232,261-263)^

In 2005, Yang *et al*.^263)^ compared the effectiveness of the 585 nm dye laser and 1,064 nm Nd:YAG laser in 17 cases of CM but observed no significant difference. They applied the 1,064 nm Nd:YAG laser 3 times and reported the improvement rate was 75–100% in 6% and 51–75% in 31%. As adverse effects, scar formation was observed at output levels that caused purpura. The skin type was I–IV.

####### V．Lasers other than dye lasers (phototherapy)^207,264-275)^

In 1999, Raulin *et al*.^274)^ applied phototherapy to 40 CM lesions in 37 patients and reported improvement rates of 70–100% in 28 lesions. As adverse effects, purpura was noted in 76%, superficial blisters were noted in 8%, crust formation was noted in 20%, depigmentation was noted in 8.1%, and pigmentation was noted in 2.7%, but no scar formation was observed. The patients included 11 who previously underwent argon laser or dye laser treatment and 1 who previously underwent liquid nitrogen therapy. The skin type was I–III. In 2004, Ho *et al*.^271)^ performed phototherapy in 22 previously untreated Chinese patients with skin types of III–IV. They reported that photoirradiation was performed 5–7 times at 3–4 weeks intervals and that the improvement rate was ≥25% in ≥90%, 25–50% in ≥50%, ≥50% in 40%, and ≥75% in 9% of the patients. As adverse effects, blister formation was noted in 1, and swelling persisting for 24 hours or longer was noted in 6, but as they were resolved in 1 week, they reported that the treatment is also considered a possible option for Asian patients. In 2005, Reynolds *et al*.^207)^ performed phototherapy in 15 previously untreated patients with skin types of I–III. They reported that 6 showed depigmentation, and 1 showed pigmentation as short-term adverse effects and that depigmentation and scar formation persisted for a long time in 1 patient. Four judges rated the improvement rate as 0% in 4 patients.

In 2008, Ozdemir *et al*.^273)^ performed phototherapy in 13 previously untreated patients with CM of the head and neck region and reported that the improvement rate was 59.2% and that 47% of the patients showed 50–75% improvements. In 2010, Dong *et al*.^269)^ performed phototherapy in 30 previously untreated Chinese patients with skin type IV and reported that the improvement rate was 50–75% in 53.3% of the patients and that even patients with skin type IV could be treated safely. In addition, Adatto *et al*.^264)^ performed phototherapy in 18 patients with skin types I–IV and reported that the treatment was effective and safe. In 2003, Bjerring *et al*.^267)^ performed phototherapy 4 times in 17 CM patients resistant to dye laser treatment and reported that responses were observed in 46.7% of the patients with all patients showing 50% or greater improvements. They also reported that the improvement rate was 75–100% in 85.7% of the patients who showed responses. Phototherapy was safe and effective for the treatment of CM resistant to dye lasers except in the area of the second branch of the trigeminal nerve. In 2009, Faurschou *et al*.^270)^ and, in 2010, Babilas *et al*.^266)^ performed split-face comparisons. Faurschou *et al*.^270)^ compared dye laser treatment (0.45–1.5 ms) and phototherapy and reported that the improvement rate was significantly higher by the dye laser. Babilas *et al*.^266)^ compared the results of dye laser treatment with a pulse duration of 0.45 nm, dye laser treatment with a pulse duration of 1.5 nm, and phototherapy in 11 previously treated and 14 previously untreated patients with CM and reported that the improvement rate differed significantly between dye laser treatment with a pulse duration of 0.45 ms and phototherapy but not between dye laser treatment with a pulse duration of 1.5 ms and phototherapy.

In these papers, different types of phototherapy were used, and the therapeutic energy and treatment intervals varied, preventing comparative evaluation of the therapeutic effect, but the results suggest possible effectiveness of phototherapy for CM. There were only 2 papers that evaluated whether phototherapy was more effective than dye laser treatment in the same cases, but as 1 paper considered dye laser treatment to have been more effective than phototherapy while the other reported no difference, phototherapy is not considered to be more effective than dye laser treatment. Regarding adverse effects, both papers found no difference between dye laser treatment and phototherapy, but they still need to be used carefully. Among case reports concerning adverse effects, in 2014, Crabb *et al*.^268)^ reported a case in which phototherapy for CM of the medial angle of the eye caused inflammation of the iris, which required steroid eye drop treatment. In treatment of areas around the eye, attention to adverse effects other than common ones, such as pigmentation, depigmentation, blister formation, and scarring, is needed.

####### VI．Lasers other than dye lasers (combined laser therapy)^233,276-278)^

In 2009, Alster *et al*.^276)^ treated 25 patients with dye laser-resistant CM (skin types I–III) by dual-wavelength continuous-wave irradiation, in which the 595 nm dye laser at 0.5–1 ms was applied followed by the 1,064 nm Nd:YAG laser, and reported 25–50% improvements in 48% and 1–25% improvements in 52% of the patients. Although pain was stronger by this combination than by dye laser treatment alone, no complications, such as abnormal pigmentation and scar formation, were noted. In 2010, Ohshiro *et al*.^278)^ reported that continuous irradiation of a long-pulse-width dye laser (variable-pulse-width dye laser) and a 1,064 nm Nd:YAG laser with a fixed pulse delay (interval) through the same light guide path was effective for the treatment of CM complicated by elevated lesions or dark purple deep lesions and CM resistant to long-pulse-width dye laser treatment. They explained that this technique complements the narrowness of the safety zone, a defect of 1,064 nm Nd:YAG laser, with continuous irradiation. In 2015, Tu *et al*.^233)^ treated 16 cases of CM with skin types III–IV by combined laser therapy (595 nm dye laser and 1,064 nm Nd:YAG laser) and observed 62.5% improvement after 5 treatments. Since pain was strong, anesthetic cream was used in 18.75% before treatment. Purpura and edema were observed only immediately after laser treatment, and purpura accompanied by blisters was noted in 25% of the patients, CM disappeared within 1–2 weeks without complications, and no scar remained. In 2018, Wang *et al*.^277)^ compared the effectiveness of combined laser treatment (595 nm dye laser and 1,064 nm Nd:YAG laser) and 595 nm dye laser treatment alone in the same 61 CM patients with skin types III (72%)–IV (28%). They reported that no significant differences were observed in the improvement rate between the 2 regimens, that 10% of the patients showed blister formation at the sites of both treatments, 2% showed blister formation only after dye laser treatment alone, 31% showed blister formation only after combination laser treatment alone, and 5% developed scars as complications after combination laser therapy.

Combination laser therapy is suggested to be effective for cases resistant to dye laser, but precautions are needed against scar formation as an adverse effect.

###### <Conclusion>

To summarize the above, lasers other than dye lasers and phototherapy are regarded as treatment options for CM, but as they are more effective than dye lasers only for CM that no longer responds to dye laser treatment or elevated and thickened CM, dye laser treatment should be performed first if it is available, and other treatments may be attempted in cases resistant to dye laser treatment with sufficient informed consent.

##### 3．Evaluation of balance of benefits and risks

Benefits: If treated, the stain may be reduced in about half the patients.

Risks: Depigmentation, pigmentation, and blister formation may occur as adverse effects. Although rare, these complications may persist, or scars may develop. Pain during irradiation is tolerated with external or topical anesthesia.

##### 4．Patients’ values/wishes

Lasers other than dye lasers and phototherapy may be attempted if patients resistant to dye laser treatment wish some alternatives. They do not necessarily conform to the patients’ values or wishes.

##### 5．Cost assessment, assessment of external validity of intervention

Since these treatments are not covered by National Health Insurance in Japan, the cost is borne by the patients themselves and may significantly burden medical economy.

##### 6．Others

The endpoint of dye laser treatment is purpura formation, but the endpoint of lasers other than dye laser and phototherapy varies with the machine used.

##### 7．Summary

While lasers other than dye lasers and phototherapy are effective for CM, their effectiveness is comparable to or less than that of dye lasers for most of CM. They may be effective for CM that no longer responds to dye laser treatment or elevated or thickened CM and may be attempted with sufficient explanation including the fact that they are not covered by National Health Insurance in Japan.

#### Lay summary

Although dye laser treatment is performed as a standard treatment for CM, complete cure is difficult to achieve. Therefore, lasers other than dye lasers are studied, but none has been established as safe or effective. In undergoing laser treatments other than dye laser, it is necessary to limit their target to intractable CM and develop a treatment plan in consideration also of complications (laser treatments other than dye lasers are not covered by National Health Insurance in Japan).

### CQ15: Is propranolol safe and effective for infantile hemangioma?

Recommendation: If administered under careful monitoring, oral propranolol therapy is the first choice for the treatment of infantile hemangioma.

Strength of recommendation: 1 (strong)

Evidence: A (strong)

#### Process of preparation of recommendation

##### 1．Circumstances that make CQ an important clinical issue

There was the serendipity that regression of hemangioma was induced in a child under steroid therapy with a giant infantile hemangioma by propranolol administered for obstructive hypertrophic cardiomyopathy in 2008.^279)^ Based on this report, oral propranolol therapy began to be used for the treatment of infantile hemangioma, and its high efficacy against alarming hemangioma/life-threatening hemangioma in the proliferating phase and in patients with cosmetic problems, such as giant lesions in the face, those with ulcerated and hemorrhagic lesions and those who may develop functional impairment, has been demonstrated, resulting in its use (Hemangiol^®^, Pierre Fabre Dermatologie, Boulogne, France) as the first choice in Western countries. In addition, its effectiveness for the treatment of hemangiomas after the proliferating phase was also described. Moreover, a group of physicians used propranolol earlier due to cosmetic significance and at the request of the family even in cases of small or localized lesions, and it is also effective in such cases. Since propranolol was included in the list of insurance-covered treatments also in Japan in 2016, this is considered an important CQ.

##### 2．Evaluation of evidence

###### <Literature search>

As a result of a systematic literature search, 568 papers (385 from PubMed, 183 from JCRM) were subjected to primary screening. As a result of a primary screening, 53 papers (50 from PubMed, 3 from JCRM) were subjected to secondary screening, through which 29 papers (29 from PubMed) were adopted. As a result of hand search, 8 papers (8 from PubMed) were adopted.

###### <Evaluation>

Including RCTs, systematic reviews, and meta-analyses, 29 references were adopted^280-308)^. For example, Hogeling *et al*.^291)^ administered placebo or propranolol at 2 mg/kg per day for 6 months with randomization to 40 patients aged 9 weeks to 5 years with infantile hemangiomas in the face or sites with the potential for disfigurement. They reported significant improvements in size, redness and elevation in the propranolol group. Elevated lesions disappeared in 4 of the 19 patients in the propranolol group but none of the 18 patients in the placebo group. As for adverse events, the trial was interrupted in 1 patient due to upper respiratory tract infection, and conditions including bronchiolitis, gastroenteritis, streptococcal infection, cool extremities, dental caries and sleep disturbance were observed.

Zaher *et al*.^292)^ observed 45 patients by randomly dividing them into 15 each treated by p.o. administration, topical application and intralesional injection of propranolol. Responses were observed in 60% in the oral group, 20% in the topical ointment group and 13.3% in the injection group. No major adverse events were noted, and the trial was discontinued in 1 in the oral group and 3 in the injection group due to inconvenience or pain of the treatment.

Malik *et al*.^293)^ randomly allotted 30 patients aged 1 week to 8 months to propranolol alone, prednisolone alone or both propranolol and prednisolone. They found that the mean initial response times were lower in the propranolol group than in the prednisolone group but that there was no clear difference between the propranolol + prednisolone group and propranolol alone group. All 10 patients in the propranolol group and 9 patients in the corticosteroid group responded to the 3-month treatment. However, adverse events were observed in 2 of the 10 patients in the propranolol group (asymptomatic hypoglycemia, insomnia) and 9 of the 10 patients in the steroid group (cushingoid appearance, gastrointestinal upset), and were more frequent in the latter group.

Bauman *et al*.^294)^ performed a phase 2, investigator-blinded, multicenter RCT in 44 patients aged 2 weeks to 6 months. Many patients refused to participate in the trial for fear of being allocated to the prednisolone group, and recruiting was cancelled in the middle of the trial because of the frequent occurrence of severe adverse effects in the oral prednisolone group, resulting in implementation of the trial with 19 cases. Moreover, as the administration was discontinued early in 5 of the 8 patients in the oral prednisolone group due to the adverse effects, the trial was completed to the end in only 9 patients in the propranolol group and 2 in the prednisolone group. Propranolol or prednisolone (2 mg/kg per day) was administered p.o. until discontinued due to toxic effects or clinical response. During 4 months of treatment, no significant differences were observed between the 2 groups, for example, with regression of 5 of the 6 tumors in the corticosteroid group and 9 of the 10 tumors in the propranolol group. For long-term analyses, the effects of prednisolone appeared earlier. No significant differences were observed in the adjusted area reduction in consideration also of regression in the center of the lesion between the oral propranolol and oral prednisolone groups (P = 0.91). While the incidence of adverse events as a whole did not differ between the 2 groups, severe adverse events were observed in 1 of the 11 patients in the propranolol group but 5 of the 7 patients in the prednisolone group and were observed significantly more frequently in the latter group^294)^.

Léauté-Labrèze *et al*.^295)^ carried out an RCT in patients aged less than 4 months by comparing 7 administered and 7 not administered propranolol. Because color changes and softening were observed within 24 h, and the thickness and size of the lesions decreased within 4 weeks in the propranolol group, the treatment was considered useful for the prevention of scarring. No serious adverse effects were observed except asymptomatic mild decreases in heart rate and diastolic blood pressure.

There have also been comparisons between atenolol and propranolol and between laser and laser + topical propranolol^296,297)^. In 2015, the largest RCT was published in the New England Journal of Medicine, also reporting that propranolol was significantly effective for hemangioma compared with placebo^298)^. Hemangioma showed complete or nearly complete resolution after 6 months of treatment in 2 (4%) of 55 patients in the placebo group and 61 (60%) of 101 patients in the 3 mg/kg per day propranolol group.

Furthermore, there have also been a few systematic reviews and meta-analyses primarily of observational studies. Menezes *et al*.^299)^ reviewed 49 English-language papers published between June 2008 and September 2010, and summarized 6 studies with 10 or more patients administered propranolol (154 patients in total). Propranolol was administered to infants with a mean age of 4.5 months at a dose of 2 mg/kg per day in 65% and 3 mg/kg per day in 25.3%. Two-thirds of the patients were treated with propranolol alone. Recurrence was observed in 21% after treatment for a mean of 4.3 months, and adverse events including hypotension, somnolence, wheezing, insomnia, agitation, nightmare, cool hands, night sweat, gastroesophageal reflux disease and psoriasiform rash appeared in 18.1%.

Marqueling *et al*.^300)^ reviewed the therapeutic results in 1,264 patients (including 806 girls) in 41 reports published from 2008 to 2012 retrieved from Medline and Cochrane. The treatment was initiated at a mean age of 6.6 months at 2.1 mg/kg per day and continued for a mean of 6.4 months. The overall response rate was 98%, and the treatment was also effective in clinically problematic areas such as the face (100%), airway (100%), periorbital (98%), head and neck region (97%), and parotid gland (82%). However, recurrence was observed in 17% after treatment. Adverse effects were noted in 371 of 1,189 patients. Changes in sleep (136 patients) and acrocyanosis (n = 61) were the most frequent among them, and hypotension was observed in 44, bradycardia in 9 and hypoglycemia in 4 as serious complications. In conclusion, the grade of recommendation was 1, quality of evidence is A and propranolol was recommended as the first-line drug for complicated infantile hemangiomas. Regarding adverse effects, the grade of recommendation was 1, and the quality of evidence was A or B. While serious adverse effects may be observed, their frequency is low, and they can be usually avoided by proper monitoring at initiation of treatment.

Xu *et al*.^301)^ on the other hand, evaluated volume changes, improvement in overall appearance, visual function and adverse effects using 15 online databases. The data of 419 cases were analyzed, but meta-analysis was not performed because of the wide differences among studies. Some studies showed superiority of propranolol compared with corticosteroid in reducing volume and improving the overall appearance. No marked differences were noted in adverse effects or visual function.

In addition, in a meta-analysis of 16 studies (2,629 cases) and 25 studies (795 cases) published in 1965–2012, 69% of the patients responded to 12-month corticosteroid therapy, but the response rate to propranolol was 97% with a significant difference^302)^.

In periorbital hemangiomas, the response rate to propranolol was found to be significantly higher than that to corticosteroid by meta-analysis of papers published before 2013^303)^, and propranolol showed the strongest effects against airway hemangiomas compared with steroid, CO_2_ laser treatment and vincristine on meta-analysis^304,305)^.

Recently, Chinnadurai *et al.* performed meta-analysis by extracting 18 papers published between 1982 and 2015 retrieved from databases^306)^. The expected clearance was significantly higher in propranolol compared with steroid and control [95%, 95% Bayesian credible interval (BCI): 88–99% for propranolol; 43% (95% BCI: 21–66%) for oral steroid; 6% (95% BCI: 1–11%) for control]. The strength of evidence concerning the reduction of lesion size of hemangioma was also superior in the propranolol group compared with the steroid and control groups.

In a 2018 Cochrane review, a meta-analysis using 3 placebo-controlled trials was reported^307)^. Among the primary endpoints, no significant differences were observed in adverse effects between the propranolol and placebo groups. Regarding the clearance, only the report by Léauté-Labrèze *et al*.^298)^was analyzed, and the endpoint was considered to have been achieved in 49% in the propranolol 1 mg/kg group and 60.3% in the 3 mg/kg group, with significant differences compared with 3.6% in the placebo group.

Furthermore, Yang *et al.* performed meta-analysis by extracting 18 papers from 6 databases. The controls were other treatments including placebo, steroid, and topical therapy. The response rate was significantly higher in the propranolol group compared with the control group, but no significant differences were observed in the incidence of adverse effects or recurrence rate. The incidence of adverse effects was higher in the high-dose (3 mg/kg) group than in the medium-dose (2 mg/kg) group. Also, the treatment was more effective when the treatment period was 6 months or longer than was less than 6 months^308)^.

###### <Conclusion>

Regarding the effectiveness and adverse effects of propranolol, a large number of systematic reviews and meta-analyses based on observational studies are already present in the above papers. We, therefore, used only 4 reports^291,293,294,298)^ on interventional studies for meta-analysis.

As a result of meta-analysis, regarding “tumor reduction”, it was found that propranolol had significantly stronger reducing effects than placebo and that it had a stronger reducing effect, which, however, was not significant, compared with corticosteroid. Concerning “complications”, propranolol was compared with steroid and was shown by 2 RCTs to have significantly fewer adverse events than corticosteroid. Because this meta-analysis found significantly stronger reducing effects of propranolol compared with placebo and fewer complications compared with steroid, and because our results were similar to those of systematic reviews of many existing observational studies considered to have high-quality evidence, we considered that there was a major tendency in this CQ and judged the evidence level as A.

##### 3．Evaluation of balance of benefits and risks

As discussed above, propranolol was judged to be significantly more effective than placebo and equally or more effective compared with steroid. Concerning the safety, propranolol is considered to cause adverse effects significantly less frequently than steroid. Since, with accumulation of therapeutic results, the number of RCTs, systematic reviews, and meta-analyses has increased recently, the references are considered to have an extremely high evidence level. Also, based on similar assessments, overseas treatment guidelines also recommend propranolol as the first choice for infantile hemangiomas.^309)^

##### 4．Patients’ values/wishes

The severity of disease and burden of treatment vary widely among cases. Although there are no studies that discuss the diversity or uncertainty of the patients’ values/wishes, many patients with alarming hemangioma/life-threatening hemangioma, ulcer-forming and easily-bleeding hemangioma, disabling hemangioma, and hemangioma that poses aesthetic problems such as a large lesion involving the face wish treatment.

##### 5．Cost assessment, assessment of external validity of intervention

Since this treatment is covered by National Health Insurance in Japan, the burden on patients in terms of cost is small. In addition, the burden of treatment of infants is reduced in many local governments.

^＊^Estimated action mechanism: Beta-blockers have a wide range of actions on the blood vessels and vascular endothelium, and have various actions on cell proliferation and vascular remodeling. Thus, the mechanism of action of propranolol on infantile hemangiomas is still unclear. In vascular endothelial cells, propranolol is considered to induce vascular contraction by suppressing nitric oxide production, inhibit renin production, control angiogenesis by regulating the expression of vascular endothelial growth factor, basic fibroblast growth factor, matrix metallopeptidase-2/9, and induce apoptosis, but it may also affect pericytes and hemangioma stem cells.^310-315)^

#### Lay summary

Although the usefulness of propranolol for the treatment of infantile hemangiomas has been already established, the latest evidence concerning its efficacy and safety is summarized in this CQ. As a results of 29 reports adopted including those with a high evidence level, such as RCTs, systematic reviews, and meta-analyses, propranolol was judged to be significantly more effective than placebo and equally or more effective compared with steroid. Moreover, concerning the safety, propranolol was considered to cause adverse effects significantly less frequently than steroid. Our meta-analysis also indicated that propranolol has a significantly greater “tumor size reducing effect” than placebo and a greater, although not significantly greater, tumor size reducing effect, compared with steroid. In addition, regarding “complications”, the incidence of adverse events was found to be significantly lower by treatment with propranolol compared with steroid.

By integrating the above observations, concerning this CQ, the usefulness of propranolol is considered to be strongly affirmed, and the evidence level was rated as A.

### CQ16: What treatments are effective for ulcer formation in infantile hemangioma?

#### ①Propranolol

Recommendation: Oral propranolol therapy is recommended for ulcer formation.

Strength of recommendation: 1 (strong)

Evidence: B (moderate)

#### ②External timolol therapy

Recommendation: External timolol therapy is recommended for ulcer formation.

Strength of recommendation: 2 (weak)

Evidence: D (very weak)

#### ③Antibiotics

Recommendation: Topical and systemic administration of antibiotics is recommended, particularly, for possibly infected ulcer formation.

Strength of recommendation: 2 (weak)

Evidence: D (very weak)

#### ④Dressings

Recommendation: The use of dressings is recommended for ulcer formation.

Strength of recommendation: 2 (weak)

Evidence: D (very weak)

#### ⑤Laser therapy

Recommendation: Although laser therapy for ulcer formation by an expert may be effective in some cases, the evidence is not considered sufficient at present.

Strength of recommendation: 2 (weak)

Evidence: D (very weak)

#### ⑥Systemic administration of steroid

Recommendation: Systemic administration of steroid is recommended not to be performed for ulcer formation.

Strength of recommendation: 2 (weak)

Evidence: D (very weak)

#### Process of preparation of recommendation

##### 1．Circumstances that make CQ an important clinical issue

Ulcer formation is an important complication that may seriously affect the patient’s QOL in infantile hemangioma. According to cross-sectional analysis in a multicenter prospective cohort study in 1,096 cases of infantile hemangioma by Chamlin *et al*.^316)^, it was complicated by ulcer, which was or was not bleeding, in 173 (15.8%), the median age of the patients was 4.0 months (standard deviation, 8.5; mean, 6.6 months), and the age at the first examination was significantly lower in patients with ulcerated hemangioma (median, 3.5 months; mean, 3.98 months) than in those with non-ulcerated hemangioma. By the site, ulcer formation was observed in 21 (30%) of 71 patients in the lower lip, 25 (25%) of 100 patients in the neck and 46 (50%) of 93 patients in the perianal/perigenital area, and the frequency was statistically lowest in the upper eyelid (P = 0.0140). Ulcer formation was observed more frequently in mixed or segmental hemangiomas. Bleeding was noted in 78 lesions (41%) and was mild in 56 (29%), moderate in 11 (6%) and severe in 4 (2%). Severe bleeding occurred in 3 lesions in the limbs and 1 lesion in the face, and bleeding occurred in 2 cases at home. Two cases required blood transfusion by hospitalization, because they showed symptoms due to serious bleeding. Of the ulcerated hemangiomas, 67 (35%) were in the proliferating phase. Ulcerated hemangiomas more often required treatment (OR, 6.86; 95% CI, 3.70–12.71; P < 0.0001), and non-ulcerated hemangiomas were more often observed without treatment (OR, 19.01; 95% CI, 11.23–28.88; P < 0.0001). Ulcerated hemangiomas tended to be treated by conventional wound care or pulsed-dye lasers (OR, 2.03; 95% CI, 1.19–3.46; P < 0.0091), and non-ulcerated hemangiomas were more often treated by topical glucocorticoid administration (OR, 2.57; 95% CI, 1.49–4.43; P < 0.0007) or surgical resection (OR, 2.04; 95% CI, 1.08–3.86; P < 0.0286). However, propranolol has recently been suggested to be effective regardless of the presence or absence of ulcer formation, and as it has few adverse effects, it is expected to become the first-line treatment in the future. This CQ, which sums up the evidence concerning these treatment choices, is an important clinical issue.

##### 2．Evaluation of evidence

###### <Literature search>

As a result of a systematic literature search, 331 papers (212 from PubMed, 119 from JCRM) were subjected to primary screening. As a result of primary screening, 46 papers (43 from PubMed, 3 from JCRM) were subjected to secondary screening, through which, 27 papers (25 from PubMed, 2 from JCRM) were adopted. As a results of hand search, 2 papers (2 from PubMed) were adopted. The extracted literature included only a few papers with a high evidence level such as those of systematic reviews and RCTs and consisted mostly of retrospective studies, case series studies, or case reports.

###### <Evaluation>

####### I．Oral propranolol therapy

Hermans *et al.* conducted a comparative observational study in 20 patients with ulcerated infantile hemangioma administered propranolol and 36 not administered propranolol^283)^. Not only the color and elevation of the lesion but also pain was reduced from early after the beginning of administration. The administration was concluded before the age of 1 year in 19 patients, and no recurrence of ulcer was noted in any of these patients except that some reactivation (enlargement) of hemangioma was observed after the discontinuation in 4 of these patients. The mean time until complete cure of ulcer was 8.7 weeks, and those in whom the administration was initiated later (>3.5 months) tended to require a longer time until cure than those in whom the administration was initiated earlier (P = 0.025). Also, the period in which ulcer persisted was 8.7 and 22.4 weeks in the treated and control groups, respectively, with a significant difference (p = 0.012). Temporary sleepiness/malaise was observed in 6 patients, fussing during sleep in 2 patients, coldness of the limbs in 6 patients, anorexia in 2 patients and gastrointestinal disorders (diarrhea, vomiting) in 1 patient, but no adverse event was noted in 9 patients. Vercellino *et al.*, Sadykov *et al.*, and Lahrichi *et al.* also reported the results of prospective studies that propranolol was effective for ulcer formation^317-319)^.

Moreover, Tiwari *et al.* carried out a RCT in 64 patients aged 1 months or older with previously untreated ulcerated infantile hemangioma of the head and neck region between an oral propranolol group and oral ibuprofen + paracetamol group^320)^. The mean time until cure of ulcer was 17.93 ± 2.22 days in the propranolol group and 27.71 ± 2.33 days in the oral ibuprofen + paracetamol group with a significant difference (P < 0.001). However, there was no significant difference in pain score, which also suggested the effectiveness of propranolol for pain control. Although generalized maculopapular skin rash was reportedly observed in 3 patients in the propranolol group, the administration could be continued.

According to the systematic review by Wang *et al.*, also, of the 197 patients with ulcerated infantile hemangioma orally treated with propranolol, epithelialization was obtained in 191 including 10 who did not respond to corticosteroid. However, as a complication, hyperkalemia, although not severe, was observed in 3 patients, and attention should be paid to cases suspected to have tumor lysis syndrome^321)^. Other similar cases have been reported, and the package insert of Hemangiol^®^ Syrup for children 0.375% (propranolol hydrochloride oral solution) in Japan also indicates careful administration.

####### II．External timolol therapy

According to the systematic review by Wang *et al.* that primarily analyzed case reports of infantile hemangioma, epithelialization was observed in 39 of the 46 patients treated by external application of timolol, a β-blocker^321)^. In the retrospective study of 30 patients with ulcers 3 cm or less in diameter reported by Boos *et al.*, also, ulcer was reported to have disappeared in 21, suggesting that the treatment is useful for the treatment of relatively small lesions^322)^.

In the prospective study by Weibel *et al.*, 6 patients with relatively small infantile hemangioma in the proliferative phase were treated externally with 0.5% timolol gel with improvements in all patients. However, as an increase in the blood concentration and urinary excretion of timolol were observed, they called attention to these findings^323)^.

Moreover, according to the case report by Gill *et al.*, a 2-month-old girl with a giant ulcerated infantile hematoma involving the face to the neck was treated by topical administration of 2 drops each of 0.5% timolol and 0.2% brimonidine (which are used for the treatment of glaucoma) with the addition of external application of 2% lidocaine and oral administration of oxycodone at 0.05 mg/kg. Since adverse effects including respiratory depression or apnea considered to be due to brimonidine appeared 90 minutes after the second administration, particular precautions are necessary in the concomitant use of these drugs^324)^.

####### III．Topical and/or systemic administration of antibiotics

In a retrospective study, Kim *et al*.^325)^ topically administered antibiotics in 40 patients with ulcerated infantile hemangioma and reported that the results were better in 37 patients (92.5%), worse in none and no change in 3 patients (7.5%). They also systemically administered antibiotics in 26 patients and reported that the results were better in 24 patients (92.3%), worse in 2 patients (7.7%) and no change in no patients. In a retrospective study, Wananukul *et al*.^326)^ topically and/or systemically administered antibiotics in 41 patients with ulcerated hemangioma and reported improvement in 19 patients (46%). Also, in a retrospective study, Pandey *et al*.^327)^ treated 608 patients showing ulcer formation with an ointment containing an antibiotic (mupirocin, sodium fusidate, sisomicin or metronidazole) combined with systemic administration of an antibiotic (amoxiclav at 20–40 mg/kg per day) in those with ulcers with an area of more than 10 cm^2^ and examined the effectiveness of treatment according to the time until cure. The time until cure was 32.63 ± 13.06 days in superficial lesions, 42.89 ± 19.89 days in mixed lesions and 57.03 ± 16.12 days in extensive lesions, with a mean of 40.09 ± 19.41 days in all lesions combined, showing significant differences among the 3 groups (P < 0.05). They also reported that the time until cure was significantly longer in larger (>10 cm^2^) than smaller ulcers (P < 0.05). In this study, although whether the wounds were infected was not examined, they were reportedly contaminated and were suspected to be infected. In a systematic review, Wang *et al.* cited these data but suggested that antibiotics should be administered only when there is active infection^321)^. Also, there is no mention about serious complications of treatment.

####### IV．Dressings

In the same retrospective study, Kim *et al*.^325)^ treated 25 patients with infantile hemangioma using dressings and reported that the results were better in 23 patients (92%) and no change in 2 patients (8%). Oranje *et al*.^328)^ applied polyurethane film and reported rapid relief of pain and cure of ulcer in 1–2 months. In addition, Bauland *et al*.^329)^ treated 41 patients using a non-adhering dressing containing an antibiotic and reported that the results were good in 26 patients (63.4%), moderate in 5 patients (12.2%) and little change in 10 patients (24.4%).

####### V．Laser therapy

There were reports on the argon laser, 1,064 nm Nd:YAG laser, and 532 nm Nd:YAG (potassium titanyl phosphate, KTP) laser in the 1980s–90s, but recent reports are primarily about treatment using the dye laser. In a retrospective study, Morelli *et al*.^330)^ treated 37 patients with ulcerated hemangioma by dye laser treatment (SPTL1b^®^ [Candela Corporation, Wyland, MA, USA]; wavelength, 585 nm; spot size, 5–7 mm; energy, 5–6.8 J/cm^2^; pulse duration, 0.45 ms) and reported that the number of irradiations until cure was 1 in 26 patients (68%) and 2 in 8 patients (21%), and that the mean period from the first treatment until cure of ulcer was 2.84 ± 0.2 weeks. In a retrospective study, also, Lacour *et al*.^331)^ irradiated 8 patients with ulcerated hemangioma that resisted conventional treatments using the same equipment and reported acceleration of cure. David *et al*.^332)^ performed dye laser treatment (PhotoGenica V^®^ [Cynosure, Westford, MA, USA]; wavelength, 585 nm; spot size, 5–7 mm; energy, 5–6.8 J/cm^2^; pulse duration, 0.3–0.5 ms) in 78 patients and reported the effectiveness of laser therapy alone in 72 (92.3%). Also, in a retrospective study, Michel^333)^ performed 1 or 2 times of 595 nm dye leaser treatment (2 pulsed irradiations with a 10% overlap; spot size, 7 mm; energy, 4–8 J/cm^2^) using Dermobeam 2000^®^ (Deka MELA, Calenzano, Italy) with a cooling system and reported resolution of pain in 10 of the 12 patients. Moreover, in a retrospective study, Di Maio *et al*.^334)^ performed laser treatment in 65 patients with hemangioma with ulcer and reported that the effects were excellent and that no clear adverse events were observed, because scarring, which was noted in a few patients, did not differ markedly compared with scarring that occurs after conventional treatments.

As for relatively recent retrospective studies, Li *et al.* treated 22 patients with ulcerated infantile hemangioma using a dual wavelength laser system with a 595 nm dye laser and 1,064 nm Nd:YAG laser (Cynergy Multiplex)^335)^. They irradiated the ulcer surface at a wavelength of 595 nm, a spot size of 7 mm, an energy of 4–5 J/cm^2^, and a pulse duration of 0.5 ms and non-ulcered areas at a wavelength of 595 nm or 1,064 nm, a stop size of 7 mm, an energy of 10–12 J/cm^2^, and a pulse duration of 10 ms. Then, targeting deeper skin lesions, they added Nd:YAG laser treatment (energy: 30–35 J/cm^2^, pulse duration: 15 ms). As a result, complete cure was achieved in 20 patients (90.9%) within 2 weeks after a single laser treatment with a mean time until cure of 13.5 ± 4.5 days, and no recurrence of ulcer, re-enlargement of hemangioma, or complication was noted during a follow-up of all patients for 6–24 months.

However, Kim *et al*.^325)^ treated 22 patients with a pulsed-dye laser and reported that the results were better in 11 patients (50%), worse in 1 patient (4.5%) and no change in 4 patients (18.2%), but warned that 5 patients in the proliferating phase showed ulcer formation after irradiation. In addition, the systematic review by Wang *et al*.^321)^ also reports the appearance of new ulcers, although in non-irradiated areas, in 5 patients despite the achievement of epithelialization in all 200 patients treated by pulsed-dye laser.

####### VI．Corticosteroid

There are previous reports suggesting the effectiveness of corticosteroid for infantile hemangioma, but, according to the analysis in the systematic review by Wang *et al.*, the 31 patients who underwent oral, topical, or external corticosteroid treatment failed to respond^321)^.

In the retrospective study by Kim *et al*.^325)^, 7 patients were treated by local steroid injections, and the results were reported to have been better in 4 patients (57.1%), worse in 1 patient (14.3%) and no change in 1 patient (14.3%). They also systemically administered steroid to 22 patients and reported that the results were better in 16 patients (72.7%), worse in 1 patient (4.5%) and no change in 5 patients (22.7%). Based on these results, they considered that the treatment was effective for reducing the lesion size. Furthermore, in the retrospective study by Polites *et al*.^336)^, of the 52 patients with ulcerated infantile hemangioma, 29 were treated by oral propranolol, and 23 by oral corticosteroid. As a result, the cure rate of ulcer was 93% in the propranolol group and 74% in the corticosteroid group, and the median time until cure was 80 days and 126 days, respectively. Also, the incidence of adverse events was 24% in the propranolol group and 44% in the corticosteroid group, and ulcer exacerbation rate was 17% and 22%, respectively. No significant differences were observed in any data, and both drugs were effective, and propranolol appears to be superior in efficacy and safety.

####### VII．Others

As other options, surgery, prostaglandin ointment, dry ice, negative-pressure wound therapy, protective stoma powder, intralesional bleomycin injection, and interferon have been reported^321,325,337-341)^. Also, becaplermin (Regranex^®^), an external preparation of recombinant human platelet-derived growth factor, is a treatment for diabetic foot ulcer approved in 1997 by the Food and Drug Administration. Although it has been reported to be effective for the treatment of ulcerated hemangioma inpatient by Sugarman and in 8 patients by Metz *et al*.^342,343)^, the cases are insufficient to evaluate its effectiveness at present.

###### <Conclusion>

Because of the absence of papers with the same study design and highly similar PICO items, meta-analysis by merging data of several papers was not performed.

##### 3．Evaluation of balance of benefits and risks

Oral propranolol: Considered useful, and the evidence level is rated as B, but as some attention to “risks” is needed, the strength of recommendation was rated as 2.

External timolol application: Considered useful, but the evidence level is D, and the strength of recommendation was rated as 2. Also, as external timolol therapy is not covered by National Health Insurance in Japan, it is necessary to carefully evaluate indications.

Antibiotics: Considered useful, but the evidence level is D, reports about “risks” are few, and, consequently, the strength of recommendation was rated as 2.

Dressings: Considered useful, but the evidence level is D, reports about risks are few, and, consequently, the strength of recommendation was rated as 2.

Laser therapy: Regarding “benefits”, there are several reports on the effectiveness of laser therapy for ulcers, but many of them are relatively old reports without controls. The evidence is not considered sufficient, and further accumulation of cases is necessary. Also, laser therapy performed by an expert in limited cases may be useful as a surgical treatment. However, as there is the risk of ulcer formation as an adverse effect of laser treatment for non-ulcerated infantile hemangioma, more precautions are considered necessary in treating already ulcerated lesions, and the treatment is not recommendable to non-experts. Therefore, the evidence level was rated as D, and the strength of recommendation as 2, on the basis of the consensus of this guideline preparation committee.

Corticosteroid: Although suggested to be effective, there are also negative opinions, and, in consideration also of patients being children and the availability of other treatment options, corticosteroid is not considered to be recommendable at present. On the basis of the consensus of this guideline preparation committee, the evidence level was rated as D, and the strength of recommendation as 2.

##### 4．Patients’ values/wishes

Since ulcer formation reduces QOL, many patients wish treatment, but the severity of the disorder and burden of treatment vary widely among cases. Although the literature discussing the diversity or uncertainty of the patients’ values/wishes was not found, antibiotics and dressings are considered likely to be accepted as treatment options because of the rarity of adverse effects and ease of use, and oral propranolol and external timolol therapies because of their effectiveness. Conversely, corticosteroid causes a large burden as a treatment for infants.

##### 5．Cost assessment, assessment of external validity of intervention

Propranolol is established as a treatment for infantile hemangioma, and dressings and antibiotics have been established as basic treatments for ulcers. In a precise sense, corticosteroid is not listed as a treatment covered by National Health Insurance in Japan, and its benefits are not considered to sufficiently match the cost or resources.

#### Lay summary

Ulceration is an important complication of infantile hemangioma, and as it is extremely difficult to treat and causes problems including bleeding, it was selected as a CQ to be evaluated. Treatments, such as oral propranolol therapy, external timolol therapy, local and/or systemic administration of antibiotics, dressings, laser treatments, oral/topical/external corticosteroid therapy as well as surgery, prostaglandin ointment, dry ice, negative-pressure wound therapy, stoma protective power, intralesional bleomycin injection, and interferon, are mentioned in the 36 papers extracted. Among them, oral propranolol had the highest evidence level and was considered particularly useful.

### CQ17: Is topical therapy effective for infantile hemangioma?

Recommendation: Topical medications such as β-blocker,^＊^ imiquimod,^＊^ and adrenocortical hormone are less effective than systemically administered drugs but can be options for uncomplicated infantile hemangioma if drugs with milder adverse effects are selected.

^＊^Not covered by National Health Insurance in Japan

Strength of recommendation: 2 (weak)

Evidence: B (moderate)

#### Process of preparation of recommendation

##### 1．Circumstances that make CQ an important clinical issue

Topical medications for infantile hemangioma are less effective than systemically administered drugs but can be options for uncomplicated infantile hemangioma if drugs with milder adverse effects are selected. Particularly, topical β-blocker therapy is effective for superficial hemangioma, but there is the problem that it is not covered by National Health Insurance in Japan.

##### 2．Evaluation of evidence

###### <Literature search>

As a result of a systematic literature search, 196 papers (135 from PubMed, 61 from JCRM) were subjected to primary screening. As a result of primary screening, 85 papers (81 from PubMed, 4 from JCRM) were subjected to secondary screening. As a result of secondary screening, 58 papers (58 from PubMed, 0 from JCRM) were adopted.

Many reports were about imiquimod and adrenocortical hormone before 2009 but were about β-blockers (timolol, propranolol, carteolol) after 2010. They included 4 systematic reviews and 1 meta-analysis.

###### <Evaluation>

Studies comparing the results of topical treatments and case series studies with a relatively large number of cases were adopted as relatively high-quality papers. The topical medications used in the adopted papers were most frequently timolol^323,344-374)^, followed by propranolol^292,297,374-382)^, imiquimod^383-392)^, adrenocortical hormone^393,394)^, carteolol^395,396)^, and others^343,397)^.

###### [Topical propranolol therapy]

####### I．Comparisons with placebo

Price *et al.* searched for reports of the use of topical propranolol therapy (Embase, Medline, PubMed, Cochrane Library, period of search: 2012–2017) and performed a systematic review of 12 studies, 597 patients, and 632 lesions^382)^. Of all lesions, the condition was improved in 90%, and a size reduction of 50% or more was observed in 59%. Of all studies, responses to treatment were observed in 67–100%, and a size reduction of 50% or more was observed in 42–86%. The highest percentage of 50% or greater size reductions was observed by application of 0.5% nano-propranolol hydrogel (made more osmotic by the addition of colloidal silicone dioxide) 3 times/day, continued until a ≥90% size reduction was achieved (maximum treatment period: 11 months). The number of applications (2 or 3 times/day) or the drug concentration did not affect the therapeutic effect. No difference was observed between 2.5% and 5% propranolol gels, and both were more effective than placebo.

####### II．Comparison with oral propranolol therapy

Regarding studies that compared oral and topical propranolol therapies, the treatment response was better (86.7 vs. 66.7%), therapeutic effect appeared sooner (mean: 2.67 weeks vs. 5.87 weeks), and treatment period was shorter (3–9 months with a mean of 5.13 months vs. 5–10 months with a mean of 7.47 months) by oral therapy. The treatment was more effective when it was initiated earlier after birth (Complete response was observed in 52% of those in whom treatment was initiated 3–9 weeks, 33% of those in whom treatment was initiated 10–20 weeks, and 0% of those in whom treatment was initiated 22–52 weeks after birth). However, comparison was difficult, because the evaluation method, administration method, dose, and dosing frequency were not standardized. In addition, the evaluation method was subjective, and there was a bias risk. No systemic complication associated with topical propranolol therapy was reported. Itching erythema was reported as a local complication (8 cases, 1.3%), and no increase in the incidence of systemic complication or local complication rate was observed in studies with patients including low-birth-weight infants. It is concluded that topical propranolol therapy is not as effective as oral propranolol therapy but that it is less likely to cause complications and may be suited for relatively small and superficial lesions with a risk of aesthetic sequelae^382)^.

####### [Topical timolol therapy]

Khan *et al.* performed a systematic review by incorporating all RCTs, case-control studies, case series studies, and case reports of infantile hemangioma treated by topical timolol therapy alone^371)^. Regarding 691 patients in a total of 31 studies, the primary endpoint was defined as the treatment response, secondary endpoints as complications including re-enlargement, and response rate as the percentage of patients who showed some improvement. The response rate was 91% in the timolol group, 9% in the control group (placebo or no treatment), and the relative risk between the timolol and control groups was 8.96 (95% CI: 5.07–15.47) with no heterogeneity (P = 0.71, I^2^ = 0%). There were differences in the drug dose, treatment period, endpoint, and follow-up interval among the studies. The mean treatment period was 4.11 months (2 weeks to 12 months), and the mean duration of follow up after the end of treatment was 3–6 months (not mentioned in a majority of reports). Sleep disorder was reported in 2 cases as a systemic complication. Re-enlargement of the lesion was observed in 4 cases in 3 studies as a local complication, but the treatment was resumed, and the lesion was completely resolved, in 3 cases. Due to the risk of confounding bias, selection bias, and omitted variable bias, the overall quality of the papers was rated as moderate to low.

Also, Zheng *et al.* performed meta-analysis of 10 studies retrieved from PubMed, Embase, China National Knowledge Infrastructure (CNKI), Wanfang, and Cochrane Library by October 25, 2016 [Relevance criteria: 1) RCT or cohort study, 2) infants with hemangioma, 3) an intervention group treated with topical timolol alone and a control group treated with another drug alone, placebo, or observation alone, and 4) evaluation of the response rate and complications]^372)^. The response rate was higher by topical timolol therapy compared with laser, placebo, or observation but did not differ compared with topical propranolol therapy.

####### [Topical carteolol therapy]

Gan *et al.* randomly allocated 349 patients with superficial infantile hemangioma (previously untreated, aged 12 months or less, superficial skin lesion, lesion thickness measured by ultrasound ≤3 mm, no spontaneous regression at the beginning of treatment) to 2 groups and compared 224 treated by applying gauze moistened with 1–2 drops of 2% carteolol solution to the lesion and covering it with film or applying it 2 times/day and 125 observed without treatment (35 of the 160 allocated to this group required other treatments and excluded)^395)^. The patients were examined and photographed monthly and followed up for 6 months. The outcome was rated as complete resolution with no recurrence (class 1), partial resolution and control of enlargement (class 2), or no response or continuation of enlargement (class 3). In the carteolol group, 10.7% (24 patients) were rated as class 1, 72.3% (162 patients) as class 2, and 17% (38 patients) as class 3: in the observation group, as 5.6% (7 patients) were rated as class 1, 25.6% (32 patients) as class 2, and 68.8% (86 patients) as class 3, and no clear adverse event was noted during the treatment period.

####### [Types and frequency of adverse reactions and adverse events and others]

None of the topical medications caused systemic adverse reactions, and their adverse reactions were primarily local. Imiquimod caused pain, reddening, ulcer, or erosion relatively frequently. Timolol, propranolol, and carteolol have been reported to have caused itching, dermatitis, and ulcer, but their frequency was low. Sleep disorder has been reported in a very small number of cases. No local adverse reaction to adrenocortical hormone has been reported.

###### <Conclusion>

Although there are many reports that topical application of β-blockers was effective for the treatment of superficial infantile hemangioma, none of them compared the outcome under the same conditions. Standardization of the administration method, dose, dosing frequency, and method for the evaluation of the therapeutic effect is necessary.

##### 3．Evaluation of balance of benefits and risks

Βeta-blockers exhibit excellent effects with few adverse reactions if applied to selected patients. Although imiquimod has been reported to be as useful as β-blockers, it is not superior with regard to adverse reactions. Also, adrenocortical hormone therapy is not superior to β-blockers in effectiveness.

##### 4．Patients’ values/wishes

Although no studies have discussed the diversity or uncertainty of the patients’ values/wishes, topical medications are acceptable as a treatment option because of the mildness of adverse reactions and ease of use.

##### 5．Cost assessment, assessment of external validity of intervention

Since, at present, topical use of β-blockers or imiquimod is not covered by National Health Insurance in Japan, reports of the results of future studies are awaited.

#### Lay summary

Since reduction in lesion size was obtained with few adverse reactions in many reports that evaluated the effectiveness of topical β-blocker therapy, it is effective, although its effectiveness is inferior to oral β-blocker therapy. However, the treatment is not covered by National Health Insurance in Japan as of 2021.

### CQ18: Is compression therapy effective for infantile hemangioma?

Recommendation: Although an appropriate compression method must be performed for individual patients, compression therapy may be regarded as an option on condition that the therapy is carried out by a skilled physician with sufficient attention to skin abnormalities and growth disturbance of the site of compression or neighboring areas.

Strength of recommendation: 2 (weak)

Evidence: D (very weak)

#### Process of preparation of recommendation

##### 1．Circumstances that make CQ an important clinical issue

Compression therapy has been used as a relatively safe treatment since the time when other treatment options were few. Because of the lack of a consensus about its usefulness, discussion concerning this CQ is important.

##### 2．Evaluation of evidence

###### <Literature search>

As a result of systematic literature search, 52 papers (29 from PubMed, 23 from JCRM) were subjected to primary screening. As a result of hand search, 1 document was subjected to primary screening. As a result of primary screening, 22 papers (11 from PubMed, 10 from JCRM, 1 from another source) were subjected to secondary screening, through which 5 papers (2 from PubMed, 2 from JCRM, 1 from another source) were adopted.

###### <Evaluation>

According to a case report of ulcerated infantile hemangiomas of the limbs by Kaplan *et al*.^398)^, the ulcers of most patients showed rapid improvements and cure within 2 weeks by compression therapy using the self-adherent wrap Coban^®^ (3M, St. Paul, MN, USA) combined with topical treatment with an antibiotic ointment (or early systemic antibiotic administration when secondary infection was apparent). They concluded that, compared with antibiotic ointment alone, its combination with compression therapy was more effective and is a safe and easy treatment that promotes regression of hemangiomas.

Ochi *et al*.^399)^ reported 12 cases of infantile hemangioma (9 girls and 3 boys with a mean age of 8.4 months; sites of the lesion were limbs in 6, head and neck in 5 and trunk in 1). By treatment using elastic bandages (5 patients), Presnet^®^ (ALCARE, Tokyo, Japan)(4), supporter (1), or Elatex^®^ (ALCARE, Tokyo, Japan) and cryotherapy (2), the hemangiomas disappeared or decreased in size in 11 of the 12 patients, with only 1 (head and neck) showing no improvement. The time until the disappearance of the lesion in the 11 responders was 2 months to 3 years (mean, 19.5 months), no complications associated with compression therapy were noted, and the authors recommended early initiation of compression therapy if the site of the lesion can be compressed.

Totsuka *et al*.^400)^ treated 3 girls with parotid gland hemangiomas (mean age, 4.3 months) by splinting using a resin plate and compression using a handmade cap. The mean duration of treatment was 13 months (8–16 months), and the patients were followed up until a mean age of 4.6 years (2–7 years), resulting in clinical and echographic disappearance of hemangioma in all 3. Because infantile hemangiomas often regress spontaneously, it is impossible to conclude that they regressed due to compression therapy, but they reported the therapy to be safe and effective.

Two relatively recent review articles also support its usefulness^401,402)^.

###### <Conclusion>

Since only 3 case series studies and 2 expert opinions are available, the evidence level is extremely low and is rated as D (very weak). Because of the lack of multiple papers with the same study design and highly similar PICO items, meta-analysis by combining data of multiple papers was not performed.

##### 3．Evaluation of balance of benefits and risks

Thus, concerning factors related to “benefits” of compression therapy, there are reports that suggest the effectiveness of compression methods appropriate for sites (elastic bandages, Presnet, splinting with a resin plate). However, it must be noted that they are all old reports. Concerning factors related to “harm”, while compression is a relatively safe and simple method without reports of serious complications, the occurrence of dermatitis and growth disturbance at the site of compression or surrounding areas is possible.

In consideration of these observations, compression therapy may have more benefits than risks, the recommendation grade was rated as 2D according to a consensus of this guideline preparation committee on condition that the therapy is performed carefully by a skilled physician. Although this guideline does not exclude compression therapy, accumulation of evidence is necessary. For cases of infantile hemangioma that requires treatment, it is necessary first to evaluate treatments including oral propranolol, oral administration, local injection, or topical application of steroid, and laser therapy.

##### 4．Patients’ values/wishes

Although there is no literature that discusses the diversity and uncertainty of the patients’ values/wishes, compression therapy is accepted as a treatment option because of the rarity of adverse reactions and ease of use.

##### 5．Cost assessment, assessment of external validity of intervention

Since compression is not covered by National Health Insurance in Japan at present, it is necessary to accumulate evidence for the future.

#### Lay summary

Propranolol is the first choice for the treatment of infantile hemangioma, but its administration is not always possible depending on adverse reactions and family environment, and the treatment is occasionally ineffective although infrequently. Since the availability of other options and improvement of adjuvant therapies are necessary, compression therapy was evaluated as a CQ.

Although none of the 5 references extracted had a high evidence level, they indicated the effectiveness of compression appropriate for the site. While the treatment is relatively safe, easy to perform, and free of reports of serious adverse reactions, it is possible that compression causes dermatitis and growth disturbance at the site of compression or in neighboring areas.

In consideration of these points, compression therapy is judged to be acceptable as an option on condition that it is performed carefully by a skilled physician with attention to skin disorders and growth disturbance of the site of compression or surrounding areas based on a consensus of this guideline preparation committee.

However, further accumulation of evidence is needed, and, for infantile hemangiomas that require treatment, it is necessary to first evaluate treatments including oral propranolol, oral administration, local injection, or external application of steroid, and laser therapy.

### CQ19: What is an appropriate timing to start/stop oral propranolol therapy for infantile hemangioma?

Recommendation: There is the possibility that oral propranolol therapy for infantile hemangioma is more effective when it is started within 6 months after birth and that the risk of re-enlargement is reduced when it is ended within 12–15 months after birth. For early initiation of treatment, strict monitoring is necessary, because the advantage of treatment in infants aged less than 5 weeks is unclear, and because there have been reports of premature infants who were orally treated with propranolol and required chest compression due to apnea or a low heart rate. To obtain favorable therapeutic outcomes by oral propranolol therapy, it is important to make preparations for oral therapy from early after the onset (checking of the birth history, cardiopulmonary function, liver function), and, in actual drug administration, management including the dose adjustment and concomitant laser therapy is necessary according to the disease condition of each patient.

Strength of recommendation: 2 (weak)

Evidence: D (very weak)

#### Process of preparation of recommendation

##### 1．Circumstances that make CQ an important clinical issue

Conventionally, steroid therapy has been performed as a drug therapy for infantile hemangioma. Since oral propranolol therapy became covered by National Health Insurance in Japan in 2016, it is increasingly recognized as the first choice. The evaluation of the timing of the initiation and cessation of oral propranolol therapy and measures to control adverse reactions is one of the important clinical issues. It is necessary to assess the evidence concerning the appropriate timing of treatment on the basis of the disease condition of infantile hemangioma.

##### 2．Evaluation of evidence

###### <Literature search>

As a result of systematic literature search, 495 papers (356 from PubMed, 139 from JCRM) were subjected to primary screening. As a result of hand search, 2 papers from PubMed 2 were subjected to primary screening. As a result of primary screening, 33 papers (32 from PubMed, 1 from JCRM) were subjected to secondary screening, through which 10 papers (9 from PubMed, 1 from JCRM) were adopted. The evidence level of these references was rated low because of the limitations of the study design such as the retrospectiveness, lack of blinding, and non-random allocation.

###### <Evaluation>

Concerning reports that evaluated maximization of the effects and the timing of initiation of oral propranolol therapy, Shah *et al*.^403)^ conducted a multicenter retrospective cohort study by classifying 997 patients into those in whom treatment was initiated within 3 months after birth (453 infants, 46.2%), within 3–6 months after birth, and 6 months or more after birth, treating them for a mean of 12 months, and evaluating the therapeutic effect 12–15 months after treatment. The evaluation was made using serial clinical photographs by a visual analogue scale (VAS) of-100 (doubled in size), 0 (no change), and +100 (disappearance). The VAS score was higher by 6% (95% CI: 2.8–8.7, P < 0.001) and 6.3% (95% CI: 3.1–9.5, P < 0.001), respectively, in those treated within 3 months and 3–6 months after birth compared with those treated 6 months or more after birth. Also, re-enlargement was observed after treatment in 231 (25.3%) of the 912 infants. The risk of re-enlargement was highest in those in whom the treatment was ended within 9 months after birth and lowest in those in whom the treatment was ended 12–18 months after birth.

Also, Sondhi *et al*.^404)^ divided patients with infantile hemangioma into those in whom oral propranolol therapy was initiated within 6 months after birth (20 infants, treatment was continued to the age of 1 year) and those in whom the treatment was initiated 6–36 months after birth (9 infants, treated for 24 weeks) and reported that the VAS score was higher in those in whom the treatment was initiated within 6 months after birth.

In the guidelines of the American Academy of Pediatrics, Krowchuk *et al*.^309)^ recommended oral propranolol therapy to be initiated 5 weeks or more after birth and ended after 12–15 months.

Concerning reports from Japan, Hirano *et al*.^405)^ evaluated the effectiveness of oral propranolol therapy by dividing the patients into those in whom the treatment was initiated within 6 months after birth (24 infants) and 6 months or more after birth (10 infants) and reported that the therapeutic effect was higher in the first group.

To evaluate the appropriateness of earlier initiation of oral propranolol therapy, El Hachem *et al*.^406)^ treated 343 infants by initiating treatment within 5 weeks after birth (15 infants, mean treatment period: 9.5 months) or 5 weeks or more after birth (328 infants, mean treatment period: 6.8 months) and reported that the therapeutic effect was comparable between the 2 groups.

As for studies in which oral propranolol therapy was initiated later, Tian *et al*.^407)^ reported that the tumor volume reduction rate was ≥75% in 53%, 50–75% in 25%, 25–50% in 22%, and 0–25% in 0% of the 31 cases in which the treatment was initiated 12 months or more (mean 18.1 months) after birth for a mean of 10.1 months.

Concerning the response rate to oral propranolol, Zhang *et al*.^408)^ divided 578 patients into those in whom the treatment was initiated within 2 months, 2–8 months, and 8–12 months after birth (the number of patients in each group is unknown, mean treatment period: 6 months) and reported that the treatment response rate was 98.1%, 93.3%, and 73.7%, respectively. There was no mention about comparison of the therapeutic effect. Also, the same authors^409)^ increased the number of cases to 853, and, in 797 patients undergoing oral propranolol therapy, compared the response rate between those in whom the treatment was initiated within 8 months (n = 787) and 8 months or more (n = 66) after birth: the response rate was 92.3% and 59.1%, respectively.

Regarding the safety of oral propranolol therapy in premature infants, Kado *et al*.^410)^ initiated treatment in 5 infants born at a gestational age of 25–27 weeks with a birth weight of less than 1,000 g at a corrected age of-3 weeks to +4 weeks and reported that they could be treated without serious adverse reactions. However, Frost *et al*.^411)^ reported that treatment was initiated at a corrected age of-3 weeks in an infant born at a gestational age of 27 weeks and that chest compression was needed due to apnea and a low heart rate.

###### <Conclusion>

To summarize the above observations, there is the possibility that oral propranolol therapy is more effective when it is initiated within 6 months after birth and that the risk of re-enlargement is lower when the treatment is ended within 12–15 months after birth. The advantage of the early initiation of treatment is unclear in infants within 5 weeks after birth.

Also, concerning the use of propranolol in premature, low-birth-weight infants, its safety has not been established as there are reports that no serious adverse reactions were observed and reports that chest compression was needed due to apnea and a low heart rate. However, early treatment with strict monitoring may also be tolerated in premature/low-birth-weight infants if there is the possibility of death or severe sequelae due to the lesion. In addition, oral propranolol therapy may also be effective even after the optimal timing of its initiation. For reference, the administration period is recommended to be 6 months, as Shah *et al*.^403)^ compared 3 months and 6 months and reported that the risk of recurrence was lower in the 6-month group. There is no report of the administration continued over 6 months.

##### 3．Evaluation of balance of benefits and risks

In the evaluation of the appropriate timing of the initiation and end of oral propranolol therapy, consideration of “higher therapeutic effect” as a benefit and “the development of complications due to oral administration” and “recurrence (re-enlargement) of the lesion after the end of oral therapy” as risks is necessary.

Regarding the time of the initiation of oral propranolol therapy, the treatment is more effective when it is initiated within 6 months than 6 months or more after birth (benefit). However, in patients in whom oral therapy is initiated early, the possibility of complications (risk) must be considered. Particularly, in early introduction of oral therapy in premature infants, strict monitoring with corrected age in mind is necessary.

Regarding the time of the end of oral propranolol therapy, the risk of re-enlargement was highest in the group in which the oral therapy was ended within 9 months (risk) and lowest in the group in which it was ended 12–18 months after birth (benefit). Reports about the occurrence of complications appear to be scarce, but attention to the occurrence of hypoglycemia (risk) becomes necessary with progression of weaning and increases in activity. It is necessary to make careful judgments about the time to end the therapy in consideration of these benefits and risks, particularly, in patients who continue to be treated for a long time after the age of 1 year.

##### 4．Patients’ values/wishes

The alleviation of aesthetic impairment and avoidance of functional impairment by oral propranolol therapy may conform to the patients’ (and parents’) values and wishes. The patients’ (and their parents’) values/wishes are expected to be markedly influenced by the site and size of hemangioma and the therapeutic effect. The physician must make judgments about the appropriate time to initiate the treatment and treatment period for each case in consideration also of the patients’ (and their parents’) values/wishes.

##### 5．Cost assessment, assessment of external validity of intervention

Oral propranolol (Hemangiol^®^) is basically covered by National Health Insurance in Japan, but there is the following statement in its package insert (precautions for use related to efficacy and indications).

I．This drug should be administered only when a physician with sufficient knowledge about this drug and experience in the treatment of infantile hemangioma has judged that the benefits of this drug surpass the risk.

II．In principle, the drug should be used for the treatment of infantile hemangioma in the proliferative period requiring systemic treatment.

Concerning the appropriate timing of the initiation and end of oral propranolol therapy for infantile hemangioma to maximize its effectiveness (reduction of the tumor size, more benefits than risks), evidence that supports extension of indications of oral administration beyond the above observations is lacking at present.

##### 6．Others

As a supplement to adverse reactions to oral propranolol therapy, the literature concerning adverse events related to oral propranolol in underage patients with disorders other than infantile hemangioma was reviewed.

As a result of a systematic literature search, 91 papers (23 from PubMed, 68 from JCRM) were subjected to primary screening. As a result of hand search, 5 papers (2 from PubMed, 3 from JCRM) were subjected to primary screening. As a result of primary screening, 19 papers (16 from PubMed, 3 from JCRM) were subjected to secondary screening, through which 4 papers (4 from PubMed, 0 from JCRM) were adopted.

For disorders other than infantile hemangioma, oral administration of propranolol is occasionally performed in underaged patients for a long time of 5–10 years or in premature infants only when there is the possibility that serious complications or progression of serious disease condition can be avoided by the therapy.

Specifically, oral propranolol therapy is carried out to avoid sudden death due to hypertrophic cardiomyopathy, and wheezing, hypoglycemia, depressive mood, depression, insomnia, night terrors, impotence, hypotension, bradycardia, bronchospasm, and apnea have been reported as adverse events. To control these conditions, reduction or discontinuation of the oral administration of propranolol or treatments of adverse events are performed^412)^. Also, if propranolol is administered to patients with retinopathy of prematurity born at a gestational age of 32 weeks or less, the dose is carefully adjusted by monitoring the circulatory dynamics and blood potassium concentration in a facility such as neonatal intensive care unit. When propranolol is administered for retinopathy of prematurity, since a β-blocker is administered intensively during a fixed period, no serious adverse reaction such as death was observed during a short-term follow-up^413-415)^.

For safety conducting oral propranolol therapy, compliance with the oral administration method including oral caloric intake is important in long-term administration. In administering the drug to premature infants, careful monitoring of the blood pressure, heart rate, respiration, blood glucose, and electrolytes and dose adjustment are essential.

#### Lay summary

It is recommended to initiate oral propranolol therapy by avoiding low-birth-weight infants, newborn infants, and infants within 5 weeks after birth and to end the oral therapy within 12–15 months after birth. The treatment tends to be more effective when oral therapy is initiated early after birth. Oral propranolol therapy should be initiated/ended by consulting with the physician in charge when its benefits are judged to surpass its risks, and appropriate medication administration according to the state of living and health of the patient is necessary during the treatment. Strict monitoring is needed, particularly, in premature or low-birth-weight infants.

### CQ20: Is cryocontact therapy useful for infantile hemangioma?

Recommendation: Cryocontact therapy may be regarded as a treatment option on condition that it is performed by a skilled physician with sufficient attention to skin disorders.

Strength of recommendation: 2 (weak)

Evidence: C (weak)

#### Process of preparation of recommendation

##### 1．Circumstances that make CQ an important clinical issue

Since cryocontact therapy is advantageous in that it can be performed at a low cost by a relatively simple procedure in the examination room, it is frequently employed in clinical settings. However, as there is no consensus about its usefulness, the discussion in this CQ is important.

##### 2．Evaluation of evidence

###### <Literature search>

As a result of systematic literature search, 277 papers (207 from PubMed, 70 from JCRM) were subjected to primary screening. As a result of primary screening, 12 papers (6 from PubMed, 6 from JCRM) were subjected to secondary screening, through which 8 papers (5 from PubMed, 3 from JCRM) were adopted. As a result of hand search, 1 paper (1 from PubMed) was adopted. The extracted literature included 3 expert opinions or guidelines and 1 prospective study as mentioned below.

###### <Evaluation>

Cryocontact therapy is mentioned as a treatment for infantile hemangioma by European expert groups and in Western guidelines^416-418)^. In a prospective study with a relatively high evidence level, Goelz *et al.* compared 17 lesions treated by cryotherapy (nitrogen-cooled cryo-contact therapy, NCCT, -196℃, contact time: 2–6 seconds) and 17 untreated lesions in 13 neonates born at a gestational age of ≤34 weeks with at least 2 infantile hemangiomas (diameter <10 mm)^419)^. Primary endpoints (disappearance of the lesion at the age of 1–2 years, no or mild pigmentation, no scarring) were achieved in 13/17 lesions in the NCCT group and 2/17 lesions in the untreated group (P < 0.001). Regarding adverse events, scarring was observed in 4/17 lesions in the NCCT group, persistence of the lesion was observed in 14/17 lesions in the untreated group, and scarring after surgical resection was observed in 1/17 lesion. From these results, it was concluded that cryotherapy is useful for early regression of infantile hemangioma and prevention of sequelae of untreated lesions and produces satisfactory aesthetic outcomes.

Reischle *et al.* evaluated the effectiveness of cryotherapy at a relatively high temperature (-32℃) using a cooling machine (KRYOMED)(10 seconds×2 cycles, 4-week intervals) as marked improvement (complete disappearance of the lesion), improvement (regression of hemangioma to a flat erythema to pale erythema), or no improvement (no improvement or enlargement of the lesion) in a case series^420)^. Of the 11 patients with plaque-type infantile hemangioma, 6 showed marked improvement, and 5 showed improvement. In the 11 patients with mixed-type infantile hemangioma, superficial lesions showed marked improvement in 6, improvement in 4, and 1 showed no improvement, and subcutaneous lesions showed marked improvement in 2, improvement in 7, and no improvement in 2. Of the 2 patients with subcutaneous infantile hemangioma, 1 showed improvement, and 1 showed no improvement. In the lesions that showed improvement or better outcomes, the mean number of treatments was 2.2 in plaque-type infantile hemangioma, 5.8 in mixed-type infantile hemangioma, and 3 in subcutaneous infantile hemangioma. From these results, Reischle *et al.* considered that even mild cryotherapy at -32℃ is effective for infantile hemangioma.

In Japan, also, Yatsushiro *et al.* performed cryotherapy by applying dry ice (3–5 seconds×1, 1-month intervals) in 27 patients with infantile hemangioma (16 plaque-type lesions, 9 tumor-type lesions, 3 subcutaneous-type lesions) and reported that the outcome 3 month after the beginning of treatment was complete response in 3 (11%) and partial response 18 (67%) and that no adverse effects were observed in any patient^421)^. Hirano *et al.* treated 15 patients with infantile hemangioma by cryotherapy using a cryotherapy machine under general anesthesia and reported satisfactory results in 13 but scarring as an adverse effect in 2^422)^. Ochi *et al.* treated 9 patients with infantile hemangioma by cryotherapy using cryotherapy machine under general anesthesia and reported complete regression in 5, practical regression in 2, no change in 1, and erosion/size reduction in 1. Mild scarring occurred in all patients as an adverse effect^399)^.

###### <Conclusion>

However, as the treatment method (liquid nitrogen, dry ice, instrument used, duration of pressure application/contact), or number of treatments varied widely, heterogeneity among studies was judged to be high, and evaluation by quantitative integration was impossible.

##### 3．Evaluation of balance of benefits and risks

As observed above, concerning factors related to “benefits” of cryocontact therapy, Goelz *et al.* showed its effectiveness by a prospective study in comparison with untreated lesions (no randomization and restriction of cases to infants born at a gestational age of ≤34 weeks with at least 2 small lesions), and all case series studies reported that complete regression was observed at the age of 5 years in about half the patients by the wait & see approach although the outcomes were not compared with spontaneous regression, suggesting the effectiveness of cryocontact therapy^419,423)^.

Regarding factors of “risks”, sufficient attention to blistering, scarring, and ulceration is necessary as general precautions although the procedure is relatively safe and simple with no report of serious adverse effects. In consideration of these points, the recommendation grade was rated as 2C on condition that the treatment is performed carefully by a skilled physician on the basis of a consensus of this guideline preparation committee. Although the present guideline does not exclude cryocontact therapy, accumulation of evidence is necessary, it is necessary to first evaluate oral propranolol or oral administration, local injection, or topical application of steroid for patients with infantile hemangioma that require treatment.

##### 4．Patients’ values/wishes

Although there is no literature that discusses the diversity or uncertainty of the patients’ values/wishes, cryocontact therapy is acceptable as a treatment option because of the rarity of adverse effects and simplicity of the procedure.

##### 5．Cost assessment, assessment of external validity of intervention

Since cryocontact therapy is covered by National Health Insurance in Japan in some prefectures, the burden of the cost on patients is small.

#### Lay summary

Propranolol is the first choice for the treatment of infantile hemangioma, but it cannot be administered to all patients depending on adverse reactions and family circumstance, and it is ineffective, although in a few cases. As improvements in other options and adjuvant therapies were necessary, cryocontact therapy was evaluated as a CQ.

Of the 12 papers extracted, cryocontact therapy was effective in 1 with a relatively high evidence level and also suggested to be effective in other papers with a lower evidence level. However, while no clear adverse effects have been reported, attention to blistering, scarring, and ulceration is necessary as a general precaution in cryocontact therapy. In consideration of these observations, cryocontact therapy is acceptable as a treatment option on condition that it is performed by a skilled physician with sufficient attention to skin disorders based on a consensus of this guideline preparation committee.

However, further accumulation of evidence is necessary, and it is necessary to first evaluate oral propranolol or oral administration, local injection, or topical application of steroid, and laser therapy for patients with infantile hemangioma that require treatment.

### CQ21: What are adverse reactions to oral propranolol therapy for infantile hemangioma that need attention and measures to prevent them?

Recommendation: Adverse reactions to propranolol therapy include hypotension, bradycardia, bronchial spasm, sleep disorder, gastrointestinal abnormalities, and hypoglycemia. Since they may leave severe sequelae, precautions are needed. Risk factors of adverse reactions are a low age (<90 days) and low body weight (<5 kg), and more strict monitoring may be necessary in patients with these risk factors. There was no evidence of the effectiveness of screening for heart disease before treatment by electrocardiogram (ECG), echocardiography, and blood glucose measurement for avoiding or preventing adverse reactions. However, as summarized by review articles, high-risk cases should be treated by hospitalization, and sufficient education of parents to avoid hypoglycemia during treatment is necessary.

Strength of recommendation: 2 (weak)

Evidence: D (very weak)

#### Process of preparation of recommendation

##### 1．Circumstances that make CQ an important clinical issue

Oral propranolol therapy began to be covered by National Health Insurance in Japan in 2016 and is becoming the first choice, but evaluation of measures to control its adverse effects is necessary. In CQ21, adverse reactions observed when propranolol, which is a β-blocker, was administered to patients with infantile hemangioma are summarized, and matters including what adverse reactions can occur, mortality rate, and incidence of severe adverse reactions are considered. In addition, it is important to evaluate whether the occurrence of adverse reactions can be truly predicted by radiography, ECG, Holter ECG, ultrasonography, and blood tests, which are performed before treatment, checking of vital signs at the beginning of treatment and before and after dose increases, or their monitoring during treatment, to validate the setting of the time and timing of oral administration and the dose, which are adjusted to prevent adverse reactions, to evaluate the optimal preventive measure, and to check whether there are routine but unnecessary pretreatment assessment, checking and monitoring of vital signs, or preventive measures.

##### 2．Evaluation of evidence

###### <Literature search>

As a result of systematic literature search, 479 papers (340 from PubMed, 139 from JCRM) were subjected to primary screening. As a result of primary screening, 67 papers were extracted. As a result of secondary screening, 16 papers (1 in Japanese, 15 in European languages) were extracted. The evidence level of these references was low, because they were overseas reports or had limitations of the study design such as retrospectiveness, lack of blinding, and lack of randomization and rated as D (very weak).

###### <Evaluation>

[Types and frequencies of adverse reactions and adverse events, and others]

According to the data of postmarketing surveillance in Japan, propranolol was administered orally to 74 patients with infantile hemangioma (administered orally at 3 mg/kg/day in 64, at 2 mg/kg/day in 9, and at 1 mg/kg/day in 1), and peripheral cold sensation in 1 patient, asthma in 2, wheezing in 1, diarrhea in 6, soft stool in 1, urticaria in 1, and reduced blood pressure in 1 were reported as adverse reactions. No severe adverse reaction was reported (Ozeki *et al.*, 2019)^424)^.

As for meta-analysis, Yang *et al.*(2019) reviewed the outcomes of 1,839 cases in 18 studies and reported the occurrence of adverse events in 451 cases (24.52%)^308)^. The adverse events consisted of 19 cases of hypotension/bradycardia, 8 cases of bronchial spasm, 2 cases of insomnia, 13 cases of lethargy, 78 cases of sleep disorders, 45 cases of agitation, 79 cases of diarrhea/anorexia, 52 case of vomiting, 31 cases of constipation, 68 case of bronchitis, 29 cases of peripheral limb coldness, and 25 others. The incidence of adverse events was 3.6% at medium doses up to 2 mg/kg/day but was 86.22% at a dose of 3 mg/kg/day.

Concerning the effects of propranolol on the growth and development, Moyakine *et al.*(2016) designed a prospective study and compared the growth and development at the age of 4 years between 82 patients administered propranolol and the same number of those not administered propranolol^425)^. No significant difference was noted in the growth or development.

###### [Analysis of risk factors of adverse reactions/events]

Ji *et al.*(2018) retrospectively studied the incidence of adverse events in 1,260 patients at 3 facilities orally administered propranolol at 2 mg/kg/day and reported that 73% of intolerable adverse events occurred within 30 days after the beginning of treatment and that, as a result of multivariate analysis, a low age (<90 days) and a low body weight (<5 kg) were risk factors^426)^.

Concerning infants with underlying diseases, in a French nationwide survey (Droitcourt *et al.*, 2018), 11 cardiovascular events (including 9 cases of conduction disorder) were reported in the 269 patients with some underlying disease (including 133 with heart disease), and their incidence was increased by oral propranolol therapy^427)^.

Also, Ji *et al.* reported (2017) that, in 51 neonates orally administered propranolol with careful management including not allowing the feeding interval to exceed 4–6 hours, orally administering propranolol within 30 minutes after feeding, and frequently measuring vital signs and blood glucose level, 13 showed diarrhea, 11 showed sleep disorder, 7 showed bronchiolitis, 6 showed bradycardia, 4 showed vomiting, 4 showed peripheral coldness, 3 showed hypotension, 3 showed a bad temper, 2 showed bronchial hypersensitivity, 2 showed constipation, and 1 showed viral upper airway inflammation as adverse events^428)^. Regarding severe adverse reactions, 2 cases (diarrhea, bronchial hypersensitivity) required interruption, and 1 (bronchial hypersensitivity) ended in discontinuation of administration.

###### [Measures to prevent adverse reactions, examinations before treatment, and monitoring during treatment]

As for the necessity of screening for heart disease before treatment, Frongia *et al.*(2018), Lund *et al.*(2018), Yarbrough *et al.*(2016), Streicher *et al.*(2016), Raphael *et al.*(2015), Salice *et al.*(2017), Hengst *et al.*(2015), Jacks *et al.*(2015), and Song *et al.*(2015) evaluated the necessity of (Holter) ECG and echocardiography^429-437)^. Oral administration was avoided in no patient because of abnormal findings before treatment, and no increase in the incidence of adverse events was observed in patients with abnormal findings. Therefore, the necessity of ECG or echocardiography is weak. Regarding hypoglycemia, there is no literature concerning the necessity of routine measurement of blood glucose or studies on preventive measures.

###### <Conclusion>

To summarize the above discussion, symptoms including hypoglycemia, hypotension, peripheral coldness, diarrhea, constipation, and wheezing have been reported as adverse events associated with oral administration of propranolol. Intolerable adverse events are likely to occur within 30 days after the beginning of treatment, and a low age (<90 days) and a low weight (<5 kg) are risk factors. The incidence of cardiovascular events is higher in patients with underlying disease such as heart disease. Special precautions are needed in administering propranolol to neonates, but the treatment is carried out safety in most cases, although there have been instances of discontinuation of administration forced by bronchial hypersensitivity. While there are many reports that screening for heart disease before treatment is unnecessary, there is no literature on the evaluation of hyperglycemia.

##### 3．Evaluation of balance of benefits and risks

The balance between benefits and risks is discussed concerning this CQ. Adverse effects and adverse events associated with propranolol include common ones such as diarrhea, those of the circulatory system such as hypotension and bradycardia, those of the respiratory system such as bronchoconstriction and wheezing, and others including hypoglycemia. All these are effects are risks to patients, and may even lead to sequelae or death. Also, hypoglycemia is a serious adverse effect that may lead to sequelae, but its incidence is not necessarily high. In addition, it can be avoidable by appropriate guidance and precautions such as oral medication to prevent hypoglycemia and interruption of administration when there is a risk of hypoglycemia such as infection during treatment. Hesitation of treating infantile hemangioma that need treatment due to excessive fear of uncommon adverse reactions is a disadvantage (risk) to patients. In contrast, the clarifying the frequencies and risk factors of adverse reactions is important (benefit) to patients. Implementing the preventive measures and consequent avoidance of adverse reactions is beneficial to patients, but performing unnecessary examinations before treatment and monitoring during treatment is poses risks to patients. The balance between these benefits and risks must be considered in the evaluation of the strength of recommendation in this CQ.

##### 4．Patients’ values/wishes

Avoidance of adverse reactions to oral propranolol may conform to the patients’ values and wishes. Regarding unnecessary preventive measures, examinations before treatment, and monitoring during treatment, the absence of abnormality alone is insufficient to prove the unnecessariness, and they may contribute to the patients’ sense of security and resolution of anxiety. In this sense, their elimination does not necessarily conform to the patients’ values and wishes.

##### 5．Cost assessment, assessment of external validity of intervention

Propranolol therapy and associated assessment and tests before treatment are all covered by National Health Insurance in Japan and do not cause a large burden to medical economy.

##### 6．Others

Regarding preventive measures, no literature with a high evidence level was found. However, concerning recommendable administration methods and patient education, Patel *et al.*(2014) made the following statements in a review article titled “How should propranolol be initiated for infantile hemangiomas”. They are presented for reference^438)^.

・Echocardiography is unnecessary as a routine examination before treatment (because simple infantile hemangioma is unlikely to be complicated by cardiac anomaly).

・ECG is recommended for patients with a heart rate lower than the criterion for the age, with congenital heart disorder, or with a familial history of arrhythmia. In PHACES syndrome, examination of the head and aortic arch by magnetic resonance imaging (MRI) is recommended.

・Initiation of treatment by hospitalization is recommended to patients aged less than 8 weeks, those with insufficient social support, and those with circulatory, respiratory, or glycometabolic complications. In hospitalized patients, propranolol administration is initiated at 1 mg/kg, the blood pressure and heart rate are examined 1 and 2 hours after administration, the dose may be increased to 2 mg/kg if no problem occurs after 3 administrations, and the patient may be discharged if there is no problem after 2 hours.

・Outpatient treatment by an experienced physician may be initiated in patients 48 weeks or more after birth, with social support, and without complications. In outpatient treatment, also, the blood pressure and heart rate must be monitored 1 and 2 hours after the initial administration, and the patient may be discharged if there is no problem. The dose is increased gradually over 3–7 days.

・Regarding adverse reactions (hypotension, bradycardia, hypoglycemia, wheezing), education of parents about matters including an appropriate dosing interval is important.

#### Lay summary

As adverse reactions to oral propranolol therapy for infantile hemangioma, hypotension, bradycardia, and bronchial spasm as well as symptoms including diarrhea, anorexia, vomiting, hypoglycemia, insomnia, lethargy, sleep disorders, agitation, and coldness of limb peripheries have been reported. Also, adverse reactions may occur less frequently at a medium dose (2 mg/kg/day) than at a high dose (3 mg/kg/day). Since adverse reactions are more likely to occur in young patients (<90 days), low-body-weight patients (<5 kg), and patients with underlying disorders, and precaution is needed.

Although there have been no reports that ECG or echocardiography before treatment is useful for the prevention of adverse reactions such as hypotension and bradycardia or that measurement of the blood glycose level is useful for the prediction of hypoglycemia, the evaluation remains insufficient. While oral propranolol therapy can be carried out safety in most cases, it is important to administer the drug carefully to patients with risk factors and to be ready to perform appropriate treatments and preventive measures to avoid severe adverse reactions.

### CQ22: What gastrointestinal examinations are useful for children suspected to have blue rubber bleb nevus syndrome? When should the examinations be started?

Recommendation: It is recommended to start screening by examinations including blood tests and fecal occult blood test as early as possible. In children suspected to have gastrointestinal bleeding, the usefulness of endoscopic examination, technetium-99m (Tc-99m)-labeled red blood cell scintigraphy, and single photon emission computed tomography (CT)/CT (SPECT/CT) has been reported for the identification of the source of bleeding. If no abnormality is detected by screening, there is no standard for the timing of search for gastrointestinal lesions to diagnose this disease or evaluate the future risk of bleeding. Among the examinations that led to the detection of gastrointestinal lesions in past reports, CT and MRI may be relatively less invasive and be performed early.

Strength of recommendation: 2 (weak)

Evidence: D (very weak)

#### Process of preparation of recommendation

##### 1．Circumstances that make CQ an important clinical issue

Gastrointestinal lesions of blue rubber bleb nevus syndrome (Bean syndrome) are observed in the entire gastrointestinal tract but more frequently affect the small intestine, and they are difficult to detect by examinations. Since the implementation of examinations is more difficult in children, this is an important CQ.

##### 2．Evaluation of evidence

###### <Literature search>

As a result of a systematic literature search, 197 papers (153 from PubMed, 44 from JCRM) were subjected to primary screening. As a result of primary screening, 37 papers (35 from PubMed, 2 from JCRM) were subjected to secondary screening, through which 28 papers (27 from PubMed, 1 from JCRM) were adopted. As mentioned below, the extracted literature was all case reports or case series studies.

###### <Evaluation>

Since blue rubber bleb nevus syndrome is an extremely rare disease, the literature is mostly case reports or review articles, no clinical studies relevant to CQ such as those in which the sensitivity and specificity of various tests were evaluated in a large number of cases have been reported. Therefore, examinations that were useful for detecting gastrointestinal lesions were reviewed among case reports primarily about children.

As examinations useful for detecting gastrointestinal lesions, blood tests, CT, MRI, abdominal ultrasonography, upper and lower gastrointestinal endoscopy, single/double balloon endoscopy, ultrasound endoscopy, capsule endoscopy, CT enterography, small bowel series, Tc-99m-labeled red cell scintigraphy, and Tc-99m-labeled red cell SPECT/CT have been reported^439-466)^.

Particularly, small bowel lesions are difficult to examined by endoscopy, but single/double balloon endoscopy, capsule endoscopy, CT enterography, CT, and MRI as well as upper and lower gastrointestinal endoscopy have been reported to be useful.

There is no clear criterion concerning the time to begin examination. However, as neonates who developed gastrointestinal bleeding shortly after birth have been reported^445)^, infants suspected to have this disease should be examined as early as possible. Invasive examinations are unlikely to be tolerated in infants, blood tests (presence or absence of anemia/consumption coagulopathy) and fecal occult blood test can be performed^446,450,451,455,458,460,461,464)^.

If gastrointestinal bleeding is suspected, examinations such as endoscopy, particularly double-balloon endoscopy and capsule endoscopy, Tc-99m-labeled red cell scintigraphy, and Tc-99m-labeled red cell SPECT/CT have been reported to be useful for the determination of the source of bleeding in children^440,443,446,447,449,458,464,465)^.

If no abnormality has been detected by screening tests, and if a search for gastrointestinal lesions needs to be performed non-emergently to diagnose this disease or evaluate the future risk of bleeding, there is no standard for the timing, which may vary among facilities. Among the above examinations, CT and MRI can be performed earlier and with relatively milder invasion, and are worth attempting if this disease is suspected^444,448,455,458,460,463,466)^.

There have also been reports of failure to detect lesions by these examinations^450,453,460,465)^, but the necessity of the other examinations for the gastrointestinal lesions mentioned above should be considered when the patient reaches the age that tolerates them.

###### <Conclusion>

Because of the absence of multiple papers with the same study design and highly similar PICO items, meta-analysis by combining data of multiple studies was not performed. The evidence of the literature as a whole is judged to be low, because it was mostly case series studies.

##### 3．Evaluation of balance of benefits and risks

A factor related to “benefits” of the above examinations is the possibility of early detection of gastrointestinal bleeding, which is a complication that may affect the prognosis. On the other hand, there are also highly invasive examinations as a “risk” factor, and sufficient precautions are necessary, although there has not been a report of an increase in the incidence of complications specific to this disease. In consideration of these observations, the recommendation grade was rated as 2D on the basis of a consensus of this guideline preparation committee.

##### 4．Patients’ values/wishes

Although no literature that discussed the diversity or uncertainty of the patients’ values/wishes was found, the uncertainty of values and preferences is large concerning invasive examinations.

##### 5．Cost assessment, assessment of external validity of intervention

Many examinations are covered by National Health Insurance in Japan, and the burden on patients is light.

#### Lay summary

Blue rubber bleb nevus syndrome is a disease that causes venous malformation in the skin and viscera, particularly the small intestine. Bleeding from the gastrointestinal tract, i.e., the stomach, small intestine, and large intestine, may pose problems.

This disease is often suspected first in infancy to early childhood, in which case, physicians often face problems such as to what degree of invasiveness is tolerated to examine venous malformation of the gastrointestinal tract, which is important for the diagnosis and requires the greatest attention and when such examinations should be performed in their clinical practice. Therefore, these problems are evaluated as a CQ.

The evidence level of all 28 references extracted was low, but they reported the usefulness of blood tests, CT, MRI, abdominal ultrasonography, upper and lower gastrointestinal endoscopy, single/double balloon endoscopy, ultrasound endoscopy, capsule endoscopy, CT enterography, small bowel series, Tc-99m-labeled red cell scintigraphy, and Tc-99m-labeled red cell SPECT/CT. In addition, neonates that developed gastrointestinal bleeding shortly after birth have been reported. No major complications due to examinations have been reported.

In consideration of these points, the following statement of recommendation was formulated as a consensus of this guideline preparation committee: It is recommended to start screening by examinations including blood tests and fecal occult blood test as early as possible. In children suspected to have gastrointestinal bleeding, the usefulness of endoscopic examination, Tc-99m-labeled red blood cell scintigraphy, and SPECT/CT has been reported for the identification of the source of bleeding. If no abnormality is detected by screening, there is no standard for the timing of search for gastrointestinal lesions to diagnose this disease or evaluate the future risk of bleeding. Among the examinations that led to the detection of gastrointestinal lesions in past reports, ultrasonography, CT, and MRI may be relatively less invasive and be performed early.

Although cases in which the lesions could not be detected by ultrasonography, CT or MRI have also been reported, the necessity of the other examinations of gastrointestinal lesions mentioned above will be evaluated when the patient reaches the age sufficient to tolerate them.

### CQ23: What are examinations useful for the follow-up of patients with Klippel-Trenaunay syndrome?

Recommendation: Ultrasonography and MRI are useful for the follow-up of Klippel-Trenaunay syndrome because of the ease of the procedure and non-invasiveness. Echocardiography is also recommended for cases with accompanying blood clotting abnormality, if D-dimer is high by a blood test in consideration of the possibility also of pulmonary hypertension. Plain radiography is also recommended for the follow-up of leg-length inequality.

Strength of recommendation: 2 (weak)

Evidence: D (very weak)

#### Process of preparation of recommendation

##### 1．Circumstances that make CQ an important clinical issue

Klippel-Trenaunay syndrome is a mesodermal disorder that causes soft tissue and bone overgrowth and slow-flow vascular malformation of the affected limbs. It may also be accompanied by complications including deep venous thrombosis, pulmonary embolism, and chronic blood clotting abnormality. The necessity of therapeutic intervention differs among cases, and the severity and activity of the disease vary. Therefore, assessing the evidence concerning appropriate examinations for this disorder was addressed as an important clinical question.

As items of intervention, blood tests (cell counts, biochemistry, clotting), plain radiography, limb girth measurements, plain MRI, contrast-enhanced MRI, plain CT, contrast-enhanced CT, ultrasonography, and angiography were evaluated.

##### 2．Evaluation of evidence

###### <Literature search>

As a result of systematic literature search, 247 papers (171 from PubMed, 76 from JCRM) were subjected to primary screening. As a result of primary screening, 48 papers (43 from PubMed, 5 from JCRM) were subjected to secondary screening, through which 17 papers (16 from PubMed, 1 from JCRM) were adopted. They included 9 review articles, 3 case reports, and 5 case series studies.

###### <Evaluation>

As a result of qualitative systematic review, there was no RCT focusing on CQ23 “What are examinations useful for the follow-up of Klippel-Trenaunay syndrome?”.

There was no report discussing the usefulness of follow-up examinations, and examinations mentioned in the case reports, case series studies, and review articles are summarized below.

Ultrasonography is performed for the local evaluation of venous and lymphatic vessel malformations and evaluation of thrombus formation in deep veins and acute pain^467)^. Ultrasonography has been reported to be more useful for venous thrombosis than MR angiography^468)^. For screening of thrombus formation, there were papers that discussed the usefulness of CT in addition to ultrasonography^469)^ and the usefulness of venography^470)^. Also, a 50% or greater difference in arterial blood flow between limbs may contribute to the prediction of future limb length discrepancy, and periodic ultrasonography is recommended to patients with Klippel-Trenaunay syndrome aged 1 year or older^471)^. In 1 paper, ultrasonography was used for the follow-up after sclerotherapy^195)^.

Radiography is performed primarily for the follow-up of leg length inequality, but leg length inequality is also evaluated using CT. One paper discussed the necessity of plain radiography and CT for the judgment of the time of therapeutic intervention for leg length inequality^472)^. While annual examination by plain radiography recommended after the age of 2 years to the end of bone growth^471)^, there was also the literature that recommended annual follow-up for a leg length inequality of 1.5 cm or greater^473,474)^ and that arteriography was performed if, in addition to leg length inequality, asymmetry was detected by examinations such as Doppler ultrasonography^475)^.

MRI is discussed as an examination used for the diagnosis and follow-up. MRI is essential for preoperative assessment^468)^ and useful also for the follow-up^474,476)^. It is useful for delineation of lesions in the abdomen and pelvis^470)^. The importance of vascular screening by MR venography has also been suggested^471,477)^. There was a report that MRI and lymphoscintigraphy were useful in patients in whom the differential diagnosis of Klippel-Trenaunay syndrome from lymphedema was difficult^478)^.

D-dimer and fibrinogen are used for the follow-up of LIC in Klippel-Trenaunay syndrome. An elevation of the D-dimer level is useful for the differentiation of Klippel-Trenaunay syndrome and Parkes Weber syndrome^190)^. There is a report of a disease in which Klippel-Trenaunay syndrome is complicated by heterozygous defects of Factor VIII and fibrinogen^479)^, suggesting the importance of screening of clotting function. In children, examination of the presence or absence of anemia as well as clotting abnormality is important^477)^. D-dimer and echocardiography are recommended for the assessment of pulmonary hypertension^480,481)^.

###### <Conclusion>

Because of the absence of multiple papers with the same study design and highly similar PICO items, meta-analysis combining data of multiple was not performed. On the whole, the evidence level of the literature, which consisted of case reports, case series studies, and review articles, is judged to be low.

##### 3．Evaluation of balance of benefits and risks

Since the evaluated items of intervention were examinations to be used for the follow-up, they are benefits to patients regardless of their results, and none of them are a risk. A possible risk is radiation exposure due to CT scanning, but its order of priority among examinations is not high, and it is not frequently performed.

##### 4．Patients’ values/wishes

Since the anxiety of the families, particularly, of child cases is strong, implementation of examinations at an appropriate timing conforms with the patients’ and their families’ values/wishes. It is possible that the opinion of the medical staff based on the medical viewpoint and the patients’ and their families’ values and wishes differ concerning which examinations should be performed and how frequently they should be performed.

##### 5．Cost assessment, assessment of external validity of intervention

The examinations listed above do not cause a large burden to patients or medical economy, because they are not assumed to be performed frequently for the follow-up and are covered by National Health Insurance in Japan.

#### Lay summary

Klippel-Trenaunay syndrome is a disease that causes bone and soft tissue overgrowth and low-flow vascular malformations. It is known to be complicated by deep venous thrombosis, pulmonary embolism, and blood clotting abnormality. Although examination of changes in clinical symptoms such as pain and swelling is important for the regular assessment of the disease state and the presence or absence of deep venous thrombosis, simple and non-invasive examinations such as ultrasonography, MRI, and blood tests are useful. If Klippel-Trenaunay syndrome is accompanied by blood clotting abnormality, blood tests are performed periodically. If the D-dimer level is high, echocardiography is performed with the possibility of pulmonary hypertension in mind. Also, overgrowth of the affected limbs may cause leg length inequality. Plain radiography is also performed for periodic evaluation of leg length inequality.

### CQ24: What are drug therapies effective for a syndrome presenting with combined vascular malformations?

Recommendation: Drug therapies are effective for combined vascular malformations, and patients with *PIK3CA* gene variants, in particular, are expected to respond to mTOR inhibitor (sirolimus*) and phosphatidylinositol 3-kinase (PI3K) α inhibitor (alpelisib**).

*Partially not covered by National Health Insurance in Japan, **Not covered by National Health Insurance in Japan

Strength of recommendation: 2 (weak)

Evidence: D (very weak)

#### Process of preparation of recommendation

##### 1．Circumstances that make CQ an important clinical issue

Recently, gene variants related to angiogenesis/lymphangiogenesis have been reported as causes of hemangiomas/vascular malformations. In the ISSVA classification, also, gene variants have been demonstrated in many combined vascular malformations. Among these gene variants, the PI3K/AKT/mTOR and RAS/MAPK/ERK pathways are attracting attention. Somatic activating mutations of the *PIK3CA* gene coding for the enzyme PI3Kα catalyst subunit located in the upstream of the PI3K/AKT/mTOR pathway, which is important for cell proliferation and energy metabolism, has been shown to cause a group of diseases, which are called *PIK3CA*-related overgrowth spectrum (PROS). PROS includes Klippel-Trenaunay syndrome and CLOVES syndrome, which are combined vascular malformations accompanied by overgrowth. The evaluation of the efficacy and safety of drug therapies targeting these pathways may further widen the options of treatments for combined vascular malformations.

##### 2．Evaluation of evidence

###### <Literature search>

As a result of systematic literature search, 272 papers (256 from PubMed, 16 from JCRM) were subjected to primary screening. As a result of primary screening 39 papers (36 from PubMed, 3 from JCRM) were subjected to secondary screening, through which 9 papers (9 from PubMed, 0 from JCRM) were adopted. The literature consisted of 2 systematic reviews, 2 prospective studies, and 5 retrospective studies.

###### <Evaluation>

Drug therapies for combined vascular malformations include sirolimus, an mTOR inhibitor, alperisib, a PI3Kα inhibitor, and miransertib, an AKT inhibitor. Of these drugs, the largest number of papers were about sirolimus.

Regarding systematic reviews about sirolimus, 69 of the 73 papers were about retrospective studies^482)^. Of a total of 373 patients with vascular malformations, 56 and 317 were treated by topical and oral administration, respectively, of sirolimus, and improvements in systemic symptoms and tumoral lesion size were observed in both groups. Sirolimus is known to be effective primarily for lymphatic vessel malformations, and many of the patients included in the adopted literature had lymphatic vessel or venous malformations. Reports on drug therapies for slow-flow combined vascular malformations such as Klippel-Trenaunay syndrome and CLOVES syndrome are still small in number. While no RCT with a high evidence level was found, prospective studies have been conducted to evaluate the safety and efficacy of sirolimus therapies, and a report up to phase II trials was obtained^110)^.

Concerning the report of alperisib, a PI3Kα inhibitor, it was administered to 19 patients with CLOVES syndrome, and effects including improvements in systemic symptoms and reductions in lesion size were observed^483)^.

Regarding the AKT inhibitor miransertib, a phase 0/1 pilot study was carried out in 6 patients with Proteus syndrome. The lesion size was reduced by the administration of miransertib, and it was effective for mitigating the symptoms of Proteus syndrome^484)^.

###### <Conclusion>

To summarize the above, drug therapies for combined vascular malformations include molecularly targeted therapies with attention to gene variants developing in the pathogenic course. Although the number of reports is still small, it is effective for the alleviation of symptoms. However, reports on recommendable administration periods or long-term follow-up are scarce, and future studies and reports of papers with a high evidence level are awaited.

##### 3．Evaluation of balance of benefits and risks

Sirolimus, alperisib, and miransertib were all shown to contribute to improvement in systemic symptoms, lesion size reduction, and improvement in QOL^110,482-489)^. Many of the reported adverse effects were grade 1–2 headache, malaise, erythema, and gastrointestinal symptoms, and few life-threatening adverse effects have been reported. However, as there have been cases that developed malignant tumors of the skin (basal cell carcinoma) or malignant lymphoma^110)^, reports of long-term follow-up are awaited.

In addition, there is the possibility of reactivation due to cessation of administration, and future evaluation of the administration period and criteria for discontinuation of administration is necessary, but drug therapies may have more benefits than risks.

##### 4．Patients’ values/wishes

If radical surgical treatment is difficult, drug therapies that mitigate symptoms or reduce the tumor size, which makes surgical resection possible, conform with the patients’ wishes. Drug therapies for combined vascular malformations may conform with the patients’ values/wishes unless their adverse effects are life-threatening or markedly impairs the patients’ QOL.

##### 5．Cost assessment, assessment of external validity of intervention

In Japan, the use of sirolimus for the treatment of intractable lymphatic vessel diseases began to be covered by National Health Insurance in Japan in September 2021. A detailed protocol has not been established concerning its use for combined vascular malformations, and future reports are awaited.

Also, alperisib and miransertib have not been covered by National Health Insurance in Japan. It is hoped that more drug therapies are approved in Japan by accumulation of further studies and clinical trials.

#### Lay summary

Cellular pathways involved in the development of gene variants and diseases related to vascular or lymphatic vessel malformations have emerged as the etiology of combined vascular malformations. Presently, drugs that act on pathway causing such disorders are being developed, and, if there are variants in the gene called *PIK3CA*, and drugs such as mTOR inhibitors (sirolimus) and PI3Kα inhibitors (alperisib) have been shown to be promising. Sirolimus was approved as a treatment covered by National Health Insurance in Japan for combined vascular malformations and Klippel-Trenaunay-Weber syndrome in January 2024.

### CQ25: What are appropriate treatments for Kasabach-Merritt phenomenon caused by kaposiform hemangioendothelioma and tufted angioma?

Recommendation: As treatments, sirolimus, everolimus, vincristine, surgical intervention, embolization, propranolol,^＊^ radiation, interferon-α,^＊^ steroids, and topical steroid injection were extracted. Among these treatments, reported cases that respond to sirolimus have increased recently, and systematic reviews, multiple retrospective observational studies, and papers that discussed its blood concentration and adverse reactions were observed. There were also a considerable number of reports about vincristine, but many of them were published before the spread of sirolimus. It is difficult to discuss the efficacy or safety of any treatment based on specific figures. Also, papers that referred to adverse reactions or late complications were fewer than those that referred to therapeutic effects. Concerning drugs, such as vincristine and interferon-α, there is concern over the risk of late complications, and further accumulation of information is awaited. This CQ is considered to require evaluation by study designs including RCTs.

^＊^Not covered by National Health Insurance in Japan (as of January 2023)

Strength of recommendation: 2 (weak)

Evidence: D (very weak)

#### Process of preparation of recommendation

##### 1．Circumstances that make CQ an important clinical issue

Kasabach–Merritt syndrome or Kasabach–Merritt phenomenon (KMP) is blood clotting abnormality that complicates kaposiform hemangioendothelioma (KHE) and tufted angioma (TA), but the methods for its treatment or management have not been established. Drugs including steroids, anticancer agents, such as vincristine, interferon, and propranolol, have been used for its treatment, but cases that responded to the mTOR inhibitor sirolimus have recently been reported. Other than drug therapies, surgery, radiotherapy, and embolization have also been attempted, but which of these treatments including drugs is optimal remains open. It is considered important to evaluate the optimal treatment by reviewing the efficacy, safety, and occurrence of late complications of each treatment.

##### 2．Evaluation of evidence

###### <Literature search>

As a result of a systematic literature search, 534 papers (311 from PubMed, 223 from JCRM) were subjected to primary screening. As a result of primary screening, 137 papers were extracted. As a result of a hand search, 1 paper (1 from PubMed) was subjected to primary screening. As a result of secondary screening, 46 papers (7 in Japanese, 39 in European languages) were extracted. They consisted of 1 systematic review, 13 retrospective observational studies, and 32 case reports. The evidence level of these references is rated low and is judged to be D (very weak). In this CQ, the literature concerning cases including those pathologically or clinically diagnosed with KHE or TA complicated by Kasabach-Merritt syndrome or KMP was reviewed. Also, since the disease is more often called KMP than Kasabach-Merritt syndrome, it is uniformly referred to as KMP in this CQ.

###### <Evaluation>

Screening of the literature showed that treatments for KHE and TA complicated by KMP include sirolimus, everolimus, vincristine, surgical intervention, embolization, propranolol, radiotherapy, interferon-α, steroids, and local steroid injection. These treatments are discussed individually in the following sections.

####### ■Sirolimus

Concerning the use of sirolimus, 17 papers consisting of 1 systematic review, 4 retrospective observational studies, and 12 case reports were adopted. In the systematic review (Freixo *et al.* 2020), 162 patients with vascular tumor and 211 with vascular malformation orally administered sirolimus were evaluated. These patients included 121 with KHE and 8 with TA, and while the number of patients with KMP is not mentioned, clinical improvements were observed in 95.5%, and normalization of blood clotting abnormality was observed in 93%, in a mean of 13.7 days. Also, a reduction of the tumor size was observed in 90.1% of the patients^482)^. In a multicenter retrospective cohort study of 52 KHE patients (Ji *et al.*, 2017), 37 patients with KMP were included, 21 of them had been treated previously, blood test findings were improved in all patients after sirolimus administration, the mean period until improvement in the platelet count was 14 days, and 5 weeks were needed before improvement in the fibrinogen level^490)^. However, reactivation was observed in 2 patients treated with sirolimus alone, and additional administration of prednisolone was needed. In a single center observational study of 22 KHE patients (Zhang *et al.*, 2018), sirolimus was administered at 0.8 mg/m^2^, and some response was observed in all patients, although the responses were poor in those previously administered prednisolone^491)^. There is a report of the use of sirolimus as the first-line treatment (Wang *et al.*, 2018), and, in a retrospective observational study of 8 KHE patients, sirolimus was administered at 0.05 mg/kg, and responses were observed in all patients^492)^. There is also a report of a neonate with KMP in whom sirolimus was effective by administering it first at 0.8 mg/m^2^, but reducing the dose to 0.4 mg/m^2^, aiming at a trough blood concentration of 10–15 ng/mL (Cabrera *et al.*, 2020)^493)^. There are other reports about the effectiveness of sirolimus in patients who did not respond to the initial treatment with other drugs including steroids, e.g., those by Tasani *et al.* (0.8 mg/m^2^ in those aged <6 months, 2 mg/m^2^ in those aged ≥6 months)(2017), Wang *et al.* (0.8 mg/m^2^ × 2/day, target trough blood concentration: 10–15 ng/mL)(2019), Yao *et al.* (0.8 mg/m^2^)(2020), Tan *et al.*(2018), Takemori *et al.*(0.25 mg/kg to 1.0 mg/kg)(2018), Kumagai *et al.*(1.6 mg/m^2^, target trough blood concentration: 5–15 ng/mL)(2018), Cashell *et al.*(2018), and Alaqeel *et al.*(target trough blood concentration: 7–10 ng/mL)(2016)^494-501)^. Brill *et al.*(2020) compared sirolimus alone and a combination of sirolimus and arterial embolization by administering sirolimus with a target trough blood concentration of 4–15 ng/mL and reported that KMP improved earlier in the combination treatment group (median time until improvement: 7 days vs. 3 months in the sirolimus alone group)^502)^. Mariani *et al.*(2019) administered sirolimus at an initial dose of 0.8 mg/m^2^, maintaining the trough blood concentration low at 2.3–6.3 ng/mL, and reported that the treatment was effective^503)^. Regarding adverse reactions to sirolimus, Ying *et al.*(0.1 mg/kg)(2018) and Wang *et al.*(target trough blood concentration: 10–15 ng/mL)(2020) reported patients that died due to infective pneumonia despite response of KMP to sirolimus administration^504,505)^. Russell *et al.*(2018) reported a case in which KMP was improved by sirolimus treatment but was complicated by carinii pneumonia^506)^.

####### ■Everolimus

Chiguer *et al.*(2020) reported a case that did not respond to prednisolone, acetylsalicylic acid, and ticlopidine but responded to everolimus at 0.1 mg/kg^507)^. In these cases, everolimus was used, because sirolimus was not available.

####### ■Vincristine

Regarding the use of vincristine, 10 papers consisting of 2 retrospective observational studies and 8 case reports were adopted. In a retrospective study (Haisley-Royster *et al.*, 2002), vincristine (1–1.5 mg/m^2^ or 0.05–0.065 mg/kg) or a combination of vincristine and prednisolone or interferon-α was administered to 15 patients with KMP, including 5 with KHE and 3 with TA, in whom prednisolone, interferon-α, and embolization were ineffective, and despite the increase in the platelet count to ≥20,000/μL after 4 weeks and recovery of fibrinogen level to ≥50 mg/dL after 3.4 weeks, recurrence was observed in 4^508)^. In a retrospective observational study of 37 KHE patients (Wang *et al.*, 2015), vincristine 0.05 mg/kg was effective for steroid-resistant and recurrent KHE, and the platelet count normalized in 26 after a mean of 7.6 weeks and in 25 patients with hypofibrinogenemia after a mean of 8.2 weeks^509)^. The treatment was ineffective in 8, and other treatments including sirolimus was necessary. Concerning vincristine, aspirin, and ticlopidine, Fernandez-Pineda *et al.*(0.05 mg/kg)(2010) and Fernandez-Pineda *et al.*(0.05 mg/kg)(2013) reported cases that showed improvements in KMP^510,511)^. In addition, Owada *et al.*(2017), Hyakuna *et al.*(2007), Jiang *et al.*(2017), and Garcia-Monaco *et al.*(2012) reported cases in which combinations of vincristine with treatments, such as interferon-α and sclerotherapy, were effective for KMP^512-515)^. Among old papers, there is a case report that KMP associated with KHE was successfully treated with vincristine, actinomycin D, and cyclophosphamide (Hu *et al.*, 1998)^516)^.

####### ■Surgical intervention

There is a retrospective study of 13 surgically treated TA patients (Lei *et al.*, 2018), of whom resection was complete in 10 and incomplete in 3^517)^. The platelet count normalized 1–3 days after surgery. Rapid improvements in KMP by surgical intervention were also reported by Shabtaie *et al.*(2016), Tatsuta *et al.*(2014) and Pascal *et al.*(2017)^518-520)^. Maeoka *et al.*(2020) reported cases in which KMP was improved by surgical drainage^521)^.

####### ■Embolization

Shen *et al.*(2014) and Garcia-Monaco *et al.*(2012) reported that embolization was effective against steroid-resistant KMP in 8 and 2 KHE patients, respectively, but some patients required vincristine administration due to reactivation^515,522)^.

####### ■Propranolol

In a retrospective observational study to examine the effectiveness of propranolol (Chiu *et al.*, 2012), 9 KHE patients and 2 TA patients administered propranolol at 0.3–3 mg/kg were evaluated, and the treatment was effective in 4 of the 11 patients^523)^. There is also a case report that propranolol became effective by increasing the dose from 2 to 3.5 mg/kg (Filippi *et al.*, 2016)^524)^. In 1 case report, KHE responded to a combination of propranolol (2 mg/kg) and vincristine (1.5 mg/m^2^), which was administered only 4 times (Hermans *et al.*, 2011)^525)^.

####### ■Radiotherapy

In a report of a retrospective observational study of radiotherapy for KMP (Kawamori *et al.*, 1995), KMP was improved at the end of radiotherapy in 4 of the 5 patients, but 1 died without responding^526)^. Other cases treated by irradiation were reported by Watanabe *et al.*(2011), Kodama *et al.*(2014), and Yamamoto *et al.*(2016), with improvements in KMP observed within 3 days after irradiation^527-529)^.

####### ■Interferon-α

Concerning interferon-α, Wu *et al.*(2016) carried out a single center retrospective observational study and reported that the administration at 1 to 3 × 10^6^ U/m^2^ was effective and safe in 12 patients with recurrent KHE^530)^. In a single center retrospective observational study by Kim *et al.*(2016), 11 of the 13 KMP patients were treated with prednisolone + interferon-α (10^5^ U/m^2^), and responses were observed in 10^531)^.

####### ■Steroids

Of the 45 papers collected, steroids were administered systemically in 30. Steroids alone were ineffective in all these reports, and it was simultaneously or subsequently combined with other treatments.

####### ■Topical steroid therapy

In a retrospective observational study of 6 cases (Lee *et al.*, 2012), KMP was reported to be improved by 2-to 6-month topical steroid therapy^532)^.

####### ■Others

In a review article on KHE, Schmid *et al.*(2018) summarized the treatment methods and outcomes in a total of 231 cases consisting of a series of 16 cases in addition to 215 cases extracted by a systematic review^533)^. Complete resection was performed in 42 cases including 14 with complicating KMP, resulting in cure without recurrence. In unresectable cases, improvements in KMP were observed in 29% of the patients treated with steroids (32% of those treated with steroids alone), 47% of those treated with vincristine (78% of those treated with vincristine alone), and 44% of those treated with interferon-α (92% of those treated with interferon-α alone). Also, KMP was improved in about 1/3 each of the patients treated by embolization, radiotherapy, or propranolol therapy. Responses to aspirin with ticlopidine or dipyridamole were observed in 2/3 of the patients. Sirolimus was used in 34 cases, of which sirolimus was used alone in 29 but in combination with drugs including vincristine, steroids, and cyclophosphamide in 5, with improvements in KMP in 30 of the 31 cases. In this study, adverse reactions to the drugs were not investigated, but the authors stated, by citing the literature, that poorly responding steroids, radiotherapy, propranolol, or embolization should not be used as the first-line treatment because of late complications and that interferon-α is not recommended due to concern over neurological complications. They also mentioned that the administration period of sirolimus has not been established.

In a report summarizing the treatment courses of 11 cases encountered in a Japanese hospital by Yasui *et al.*(2013), steroids were the first choice, but its efficacy rate was low at 20% (2/10), and, although interferon was added in 6, it was effective in only 16% (1/6)^534)^. As the second choice, chemotherapy (vincristine, cyclophosphamide) was selected in 6 (55%), and it was reportedly effective in 83% (5/6, vincristine regimen in 3/5) with no recurrence. Propranolol was used in combination with other treatments in 3 but was not effective in any of them. According to this report, for steroid-resistant cases, vincristine rather than interferon is preferred as the first choice, and, if it is ineffective, chemotherapy including cyclophosphamide is recommended.

###### <Conclusion>

Analysis was performed to evaluate the CQ “What are appropriate treatments for Kasabach-Merritt phenomenon caused by kaposiform hemangioendothelioma and tufted angioma?”, but no paper with a high evidence level, such as a prospective study, was found. By literature screening, sirolimus, everolimus, vincristine, surgical intervention, embolization, propranolol, radiotherapy, interferon-α, steroids, and topical steroid therapy were extracted as treatments. Among them, reports of cases in which sirolimus was effective are increasing recently, and a systematic review and multiple retrospective observational studies as well as reports referring to the blood concentration and adverse reactions of sirolimus were observed. In Japan, a clinical trial (investigator-initiated multicenter phase III trial to evaluate the efficacy and safety of NPC-12T (granules/tablets) against refractory vascular tumor/vascular malformation) is underway, and the release of the results is awaited. There were also many reports concerning vincristine, but many of them were published before the spread of sirolimus. For any treatment, it is difficult to determine the effectiveness or safety based on specific values. Also, papers that addressed adverse effects or late complications were fewer than those that referred to therapeutic effects. There is concern over the risk of late complications by drugs, such as vincristine and interferon-α, and irradiation, and further accumulation of information is necessary. For the future, the evaluation of this CQ is considered to require validation by a research design such as RCTs.

##### 3．Evaluation of balance of benefits and risks

The balance between benefits and risks concerning this CQ is discussed. Drug therapy, surgical intervention, and radiotherapy are major treatments for KMP, and the selection of a treatment with high efficacy is beneficial to patients. However, these treatments have the risk of various adverse reactions and, occasionally, sequelae, and they have harmful effects (risks), particularly, on this disease, which develops in infancy. In these reports, matters, such as late complications of radiotherapy, are not mentioned at the time of their publication. There is also concern over the risk of severe adverse reactions to not only sirolimus, which was developed recently, but also drugs such as vincristine and interferon. However, KMP may be lethal in some patients if left untreated, and the avoidance of treatment for fear of such adverse reactions and complications is not an option (risk) to the patients. In the literature reviewed concerning this CQ, treatments were considered effective in most reports while which treatments were effective remains unclear. Also, data concerning the frequency of adverse effects of treatments or their severity and late complications are not sufficient. In evaluating the strength of recommendation concerning this CQ, the balance between these benefits and risks must be considered.

##### 4．Patients’ values/wishes

Selecting treatments that are highly effective for KMP is considered to conform to the patients’ values and wishes. By pursuing higher efficacy, resolution of KMP and improvement in survival rate can be achieved for the time being, but selecting treatments that are likely to cause late complications is not necessarily in conformity to the patients’ values and wishes.

##### 5．Cost assessment, assessment of external validity of intervention

Steroids, vincristine, surgical intervention, embolization, and radiotherapy are all covered by National Health Insurance in Japan and do not cause a large burden on medical economy. However, many other drug therapies (e.g., sirolimus, everolimus, propranolol, interferon-α) are presently not covered by National Health Insurance in Japan and must be administered in clinical trials or clinical studies. Concerning sirolimus, a clinical trial (investigator-initiated multicenter phase III trial to evaluate the efficacy and safety of NPC-12T (granules/tablets) against refractory vascular tumor/vascular malformation) is underway, and the release of the results is awaited.

#### Lay summary

Kasabach-Merritt syndrome (recently called KMP) is severe blood clotting abnormality associated with KHE and TA, but its treatment has not been established. There have been reports of a variety of treatments including sirolimus, everolimus, vincristine, surgical intervention, embolization, propranolol, irradiation, interferon-α, steroids, and topical steroid therapy, but which treatments are more recommendable remains unclear. Recently, expectations for the effectiveness of sirolimus have increased, and it was approved as a treatment covered by National Health Insurance in Japan for hemangioendothelioma and TA in January 2024.

### CQ26: Is surgical resection effective for soft tissue/superficial LM?

Recommendation: Although surgical resection is an effective treatment, it should be performed after a comprehensive evaluation of cosmesis, survival and functional prognosis, resectability and the possibility of recurrence/complications.

Strength of recommendation: 2 (weak)

Evidence: D (very weak)

#### Process of preparation of recommendation

##### 1．Circumstances that make CQ an important clinical issue

Surgical resection has a long history as a major treatment for LM. It can be completely removed by total resection, but total resection is not necessarily a goal, because the disorder is not malignant, and surgery is often performed for cosmetic, functional, and symptomatic improvements. Particularly, cosmetic problems are important in lesions located in shallow areas such as the body surface and soft tissue. However, as surgical resection is known to be associated with complications including bleeding, infection, disfigurement, and neuroparalysis, the indications for surgical resection can be difficult to determine.

##### 2．Evaluation of evidence

###### <Literature search>

As a result of a literature search, 132 papers in Japanese and 759 papers in European languages were retrieved. Through primary screening, 6 papers in Japanese and 79 papers in European languages were subjected to secondary screening. They did not include the literature with a high evidence level, such as RCTs, with the exception of 1 systematic review, and the others were case series or case reports. Therefore, in the recommendation statement concerning this CQ, the results and discussions in each case series were integrated, and the literature considered useful was presented as review data despite inadequacy of evidence.

###### <Evaluation>

The effectiveness of surgical resection of LM was evaluated from the following considerations: (i) effectiveness of surgical resection for extending the survival period (survival rate or mortality); (ii) resection rate of the lesions (resectability); (iii) functional outcome after resection (function); (iv) recurrence rate (recurrence); and (v) complications (complication).

The outcomes concerning the survival varied considerably, and factors, such as the site, size, and extent of the lesion, were considered to be involved in the state of survival after surgical resection^535)^. The resection rate of lesions was cystic>mixed> cavernous types in descending order^536,537)^. According to the site, the total resection rate was high in localized primary lesions in the head and neck region but low in those infiltrating the surrounding areas^537)^. Lesions at other sites are also totally resected if they are non-infiltrative and well-circumscribed, but lesions that involve important organs, such as the blood vessels, esophagus, and trachea or nerves, are often treated by fenestration and endoluminal cauterization, and lesions with extensive infiltration into the parotid gland, oral cavity, tongue, and cranium are often managed by partial resection^536)^. Few reports have evaluated the functional outcomes of surgical resection using objective data. However, regarding primary LM of the orbit, there have been reports of improvements in visual function and cosmesis such as improvements in visual acuity and alleviation of exophthalmos^538-540)^. The recurrence rate is considered to be high after resection of lesions that extend to multiple regions^541-543)^. The recurrence rate was higher in patients with oral or facial lesions than in those with neck lesions. Also, when the lesions were classified according to the site into suprahyoid or infrahyoid and unilateral or bilateral lesions, the recurrence rate and complication rate were, in ascending order, unilateral and infrahyiod<unilateral and suprahyoid<unilateral and supra-infrahydoid<bilateral and suprahyoid<bilateral and supra-infrahyoid. Complications after surgical resection of head and neck lesions are relatively common, and conditions including facial nerve paralysis, salivary fistula, hoarseness, disfigurement, and airway obstruction have been reported^544-547)^. Particularly, facial nerve paralysis is likely to be caused by resection of a lesion occupying the parotid region and infiltrating surrounding tissues^544)^.

####### ■Limitations

In the adopted literature, reports prospectively evaluating the results of surgical resection performed according to predetermined indications and those retrospectively evaluating the indications based on the results obtained are mixed. Therefore, the criteria for surgical resection are not uniform, and differences in the background of the subjects must be considered in evaluating the effectiveness of surgical resection.

###### <Conclusion>

In evaluating the CQ “Is surgical resection effective for soft tissue/superficial LM?”, the effectiveness was analyzed from five considerations, i.e., effectiveness of surgical resection for extending the survival period (mortality), resection rate of the lesions (resectability), functional outcome after resection (function), recurrence rate (recurrence), and complications (complication). While there was no paper with a high evidence level, differences were observed in functional outcome, recurrence rate, and contents or incidence of complications according to the lesion type (cystic or cavernous), the site of the primary lesion, and relationship with other treatments. Many reports showed that the functional outcome was poor, and the recurrence rate and complication rate after resection were high, in patients with lesions occupying a large area or with symptoms such as airway obstruction. In contrast, some reports suggested the effectiveness of surgical resection in patients with cystic LM or those unresponsive to sclerotherapy. Research by designs, such as RCT, is considered necessary for the future for precise evaluation of the effectiveness of surgical resection.

##### 3．Evaluation of balance of benefits and risks

Whether surgical resection is effective is judged according to the balance between its benefits and risks such as complications. For soft tissue/superficial LM, cosmetic improvement is important. No inclusive selection criteria for surgical resection have been established since there are no criteria for the cosmetic outcome, and since the incidence of complications, cure rate, and recurrence rate differ according to the site of the lesion. However, it is evident that there are cases in which benefits of surgical resection surpass its risks, and decisions should be made after evaluation of individual cases.

##### 4．Patients’ values/wishes

Although the patients’ values/wishes differ according to the site of the lesion, not only cosmetic but also functional improvements have high priority, particularly, in facial (orbital) or oral lesions. There are cases in which symptoms are not improved by conservative treatment, and maximum possible surgical resection may be necessary. However, as lesions are not localized, they are often resected only partially. Since lesions in other areas are often surgically resected not only for treating infection and symptoms, such as bleeding, but also for cosmetic improvements, treatment must be selected in consideration of risks associated with scarring of the surgical wound. Also, as evaluated in CQ27, sufficient evaluation with the possibility of changes in the values depending on the treatment period in mind is necessary.

##### 5．Cost assessment, assessment of external validity of intervention

Along with sclerotherapy, surgical resection is already approved as a treatment covered by National Health Insurance in Japan, and its validity as a treatment per se is established. However, there is the possibility of generation of unexpected cost due to the occurrence of complications, and careful evaluation of indications is necessary in individual cases.

#### Lay summary

Surgical resection is a major treatment for LMs. Localized lesions may be resected totally, but lesions extending to a wide area and deeply involving the surrounding important tissues can often be resected only partially. There are reports that functional disorders often recur after surgery when only partial resection is possible. However, treatment is necessary if there are cosmetic problems that interfere with normal daily living or if functional impairment (e.g., visual disorder due to compression of the eye, jaw closing dysfunction due to enlargement of a tongue lesion) persists, and surgical resection is an option. Surgical resection has been reported to be effective, particularly, in patients with cystic lesions and those who do not respond to sclerotherapy. Since the effects of surgery and risks of complications vary widely depending on the site and size of the lesion, it is necessary to select treatment by considering all these matters.

### CQ27: What is the optimal timing of surgery for soft tissue/superficial LM?

Recommendation: It is impossible to recommend optimal timing of surgery, and judgment according to the condition of each case is necessary.

Strength of recommendation: 2 (weak)

Evidence: D (very weak)

#### Process of preparation of recommendation

##### 1．Circumstances that make CQ an important clinical issue

Soft tissue/superficial LM not a malignant disease. Emergent treatment may be necessary in infancy in life-threatening conditions, such as airway obstruction due to compression by the lesion, but the initiation of treatment immediately after the diagnosis is generally considered unnecessary. The natural course of the disease differs considerably among individuals, particularly in infancy, and the lesions may show a tendency of spontaneous regression but may also cause various functional problems due to rapid enlargement. Moreover, there are cosmetic problems characteristic of this disease in addition to functional problems, and early therapeutic effects are necessary to make social life comfortable. For these reasons, the selection of optimal timing of treatment, surgery in particular, is a major issue.

##### 2．Evaluation of evidence

###### <Literature search>

As a result of a literature search, 94 papers in Japanese and 446 papers in European languages were retrieved. Through primary screening of these papers, 6 in Japanese and 75 in European languages were subjected to secondary screening. None of these papers were on a systematic review or RCT with a high evidence level, and all were case series reports or case reports. Therefore, in the evaluation of the recommendation statement, the results and discussions of these case series reports were integrated, and those considered useful for the preparation of the recommendation statement were presented as review data although they are insufficient as evidence.

###### <Evaluation>

There were few papers about analysis with respect to “appropriate timing of surgical treatment for LM”. Data concerning the patients’ age at the time of surgery were available, but few papers evaluated the appropriateness of the age. Among the limited retrieved data, comments on the timing of surgery are shown below.

Concerning the timing (age) of surgery, its postponement until the age of 3 years is recommended in expectation of natural regression or for reasons such as the ease of identification of surrounding structures at the time of surgery, ease of control of bleeding, and simplicity of postoperative management unless the lesion is small or requires urgent treatment due to symptoms such as respiratory disorder^548)^.

Also, while the optimal timing of surgery is not mentioned, it is considered necessary to determine the time of surgery in consideration of problems that change with the patient’s age: High among priorities in patients with head and neck or giant lesions are airway protection and appropriate nutrition in neonates, control of bleeding and infection and treatment of dysarthria and dental problems in infancy, and management of skeletal and cosmetic problems in school age^549)^. There is also a report that late LM of the skin/mucosa (lymphangioma circumsriptum) that appear in adulthood is likely to be resolved without recurrence by resection with subcutaneous tissue if the lesion is localized^550)^.(See CQ37)

###### <Conclusion>

As a result of a literature search for evaluating the CQ “What is the optimal timing of surgery for soft tissue/superficial LM?”, some papers mentioned the time of surgery, but few discussed its appropriateness. Three papers that mentioned the appropriateness of the timing of surgery were analyzed, but the analysis fell short of generalization of the issue.

High-quality research is necessary for the evaluation of the appropriate timing of surgery in this CQ, but no new reports of high quality was observed after the evaluation for the preparation of the previous edition, Japanese clinical practice guidelines for vascular anomalies 2017. Concerning this disorder, it is considered extremely difficult to design clinical studies to induce general discussion by equalizing conditions, because it is often treated non-surgically first and is diverse in distribution and histological type of lesions.

##### 3．Evaluation of balance of benefits and risks

For the selection of the timing of surgical resection, it is necessary to sufficiently consider the balance between benefits and risks at each time of resection in addition to the validity of the selection of resection evaluated in CQ26. The time of surgery must be determined according to the disease and social conditions of each patient in consideration of problems that arise at different ages: High in the order of priority in the management of head and neck lesions or giant lesions are airway protection and appropriate nutritional management necessary for the development in the neonatal period, control of symptoms, such as bleeding and infection, and treatment of dysarthria and dental problems in infancy, and skeletal and cosmetic problems in school age. In addition, it is important to make comprehensive evaluation by also considering negative aspects of surgical treatment (the possibility of scarring, deformation, and complications such as delayed wound healing).

##### 4．Patients’ values/wishes

While surgical resection is useful as a treatment, it may cause scarring, deformation, and complications such as delayed wound healing (See CQ26). In infancy, the patients’ values concerning cosmetic impairment, functional impairment, and symptoms due to the lesion and those concerning benefits and risks of treatment are the values of their parents, but the patients develop self-consciousness with their growth, and discord may develop between the patients’ and their parents’ values. In addition, values concerning the disease and its treatment may change in the patients’ social activities. The timing of surgery must be determined individually in consideration of all such factors.

##### 5．Cost assessment, assessment of external validity of intervention

Surgical resection is widely recognized as an effective treatment for LM if it is selected appropriately. It is covered by National Health Insurance in Japan, and there is no major change in the treatment cost depending on the time of its implementation regardless of age. It is appropriate to propose surgical resection as a treatment option in all stages while also considering other less invasive treatments.

#### Lay summary

Since LMs vary widely in site, size, and symptoms caused by the lesion among patients, the optimal timing of surgery to obtain a favorable outcome differs in each patient. There have been few studies that scientifically evaluated the optimal timing of surgery. There is a report that surgery should be postponed until the age of about 3 years in consideration of the possibility of natural regression and the relative increase in the ease of surgery itself and the management before and after surgery unless immediately treatment is necessitated by a condition such as respiratory disorder due to airway compression by the lesion. Also, feelings and values about the disease and its treatment may change with age and social environment, and the timing of surgical treatment must be determined by careful evaluation of each of these factors.

### CQ28: What treatments are effective for facial (orbital/palpebral) microcystic LM?

Recommendation: Sclerotherapy, surgery, radiofrequency ablation, and drug therapy are considered. While they have been shown to be effective, complications, such as functional impairment, have been reported, and the treatment should be selected carefully according to the condition of each patient.

Strength of recommendation: 2 (weak)

Evidence: D (very weak)

#### Process of preparation of recommendation

##### 1．Circumstances that make CQ an important clinical issue

The face (including the orbital region) is a site of predilection for LM, and treatment for microcystic LM, in particular, often poses difficulties. Since the face is an exposed region, cosmetic problems weigh heavily, and since it includes functionally vital organs, such as the eyes and mouth, the selection of a safe and effective treatment is extremely important.

##### 2．Evaluation of evidence

###### <Literature search>

As a result of a literature search, 49 papers in Japanese and 335 papers in European languages were retrieved and subjected to primary screening. Eventually, 2 papers in Japanese and 66 papers in European languages were subjected to secondary screening for this CQ. Many of them were case series reports or case reports with the exception of 2 systematic reviews. Therefore, in the evaluation of the recommendation statement for this CQ, the results and discussions in these systematic reviews and each of these case series were integrated. Moreover, as a result of hand search for the literature concerning lymphovenous anastomoses, 1 case series report was added as a reference. Consequently, 2 papers in Japanese and 44 papers in European languages judged to be useful for the preparation of the recommendation statement were listed as review data, although they are insufficient as evidence.

###### <Evaluation>

The following were mentioned as treatments for microcystic LM of the face (including the orbit and eye lids) in the reviewed literature.

・Surgical treatment

・Sclerotherapy (OK-432, bleomycin, ethanol, monoethanolamine oleate, doxycycline, sodium tetradecyl sulfate, pingyangmycin, polidocanol foam)

・Radiofrequency ablation

・Drug therapies: Sirolimus, sildenafil, propranolol

In most reports, these treatments were performed in various combinations rather than alone.

####### ■Surgical treatment

Regarding the usefulness of surgical treatments for head and neck microcystic LM, there are few reports that compared surgery alone and its combinations with other treatments^536,551,552)^, and most of the literature was reports of the results of surgery combined with other treatments including slerotherapy^545,553-562)^.

Microcystic LM is more difficult to totally resect than macrocystic LM, and its surgical treatment has been suggested to be less radical^536,542,545,554)^. Also, surgery has often been combined preoperatively or postoperatively with other treatments, such as sclerotherapy, suggesting that refractory cases are treated multidisciplinarily.

Comparisons of the recurrence rate and complications between surgery and sclerotherapy have shown neither treatment as superior^551,563)^. In a study that compared surgery and sclerotherapy in 174 cases, the possibility that additional treatment becomes necessary within 1 year was comparable between the surgery and sclerotherapy groups: 75.3% and 83.6%, respectively^551)^. In this report, patients with microcystic LM accounted for only 34.8%, and the results were not those concerning microcystic LM alone.

Conditions including facial nerve paralysis, recurrent nerve palsy, vagus nerve palsy, transient respiratory, eating, and articulation disorders, hematoma/lymphorrhea, infection, salivary gland fistula, seroma, and eyelid closing disorder have been reported as complications^536,545,552-555,557-559)^. Although microcystic and macrocystic LM were mixed, Lee *et al*.^552)^ reported 16 cases that required dissection of the facial nerve. According to the report, the facial nerve was displaced by the lesion, but its course could be anticipated by preoperative CT in 10 cases.

Microcystic LM of the head and neck region is treated by combinations of various methods depending on the case, but the grounds for the selection of surgical treatments are weak. Surgical treatment was suggested to be advantageous in cases that do not respond to sclerotherapy, those that require emergency treatment due to a condition, such as airway obstruction, and those that require histological examination, but further evaluation is necessary.

####### ■Sclerotherapy

The effectiveness of sclerotherapy was evaluated from the following considerations.

I．Treatment responses

A．Size

B．Symptoms

C．Functions

D．Cosmetics

II．Complications

The contents of the accounts concerning the effectiveness of sclerotherapy are summarized according to these considerations.

The sclerosing agents mentioned in the literature varied widely, including OK-432, bleomycin, ethanol, monoethanolamine oleate, doxycycline, sodium tetradecyl sulfate, pingyangmycin, and polidocanol foam, multiple agents were occasionally used in combination.

###### I．Treatment responses

####### A．Size

Many of the papers that referred to the regression rate of the lesion classified the responses into 1) excellent or complete (regression rate ≥90%), 2) good or substantial (regression rate ≥50% and <90%), 3) fair or intermediate (regression rate ≥20% and <50%), and 4) poor or none (regression rate <20%).

Although there were few reports that collected cases of facial lesions alone, Yang *et al.* reported that the regression rate after sclerotherapy was ≥90% in 19 (63%) and ≥50% in 10 (33%) of the 30 patients with head and neck lesions^564)^. In addition, the regression rate was reported to be ≥50% in 18 (85.7%) of the 21 patients with head and neck lesions by Alomari *et al*.^565)^ and in 30 of the 31 patients, including those with mixed type lesions, by Chaudry *et al*.^566)^

On the other hand, Smith *et al.* reported that none of the 17 patients, some of whom had mediastinal lesions, showed complete or substantial response^567)^. Giguère *et al.* also reported that all 5 patients with head and neck lesions showed no response (poor) to sclerotherapy^568)^. While these studies were RCTs evaluating the time of sclerotherapy, the results suggest that sclerotherapy is ineffective for microcystic LM regardless of the time of treatment.

De Maria *et al.* conducted a systematic review and meta-analysis of 23 papers concerning sclerotherapy for head and neck LM and reported that the response of head and neck microcystic LM to sclerotherapy was complete cure in 35.1%, partial cure in 39.3%, and no effect in 17.3%^569)^. Also, the response of macrocystic LM was complete cure in 53.1%, partial cure in 15.5%, and no effect in 2.6%, and the size-reducing effect of sclerotherapy was considered weaker in microcystic LM than in macrocystic LM. According to the type of sclerosing agent, the complete cure rate of head and neck LM including macrocystic and mixed types was 62.4% with doxycycline, 61.4% with monoethanolamine oleate, 48.6% with OK-432, 31.4% with bleomycin, and 27.8% with pingyangmycin.

####### B．Symptoms

There is no published work that evaluated symptoms based on objective data, and few reports referred to symptoms. Chaudry *et al.* reported that symptoms disappeared after sclerotherapy with bleomycin in 75% of the patients who complained of pain^566)^. Also, Da Ros *et al.* reported that symptoms disappeared in 5 (31%) and were alleviated in 9 (56%) of the 16 patients with head and neck LM^570)^. Other literature includes only sporadic case reports that symptoms, such as bleeding and respiratory disorder, were relieved by sclerotherapy^571,572)^.

####### C．Functions

Ravindranathan *et al.* performed sclerotherapy in 3 patients with microcystic LM extending from the face to the tongue and pharynx and reported improvements of respiratory disorder and dysphagia due to airway stenosis observed before treatment^573)^. Poonyathalang *et al.* administered sodium tetradecyl sulfate to a patient with orbital LM primarily complaining of visual defect and reduced visual acuity due to retrobulbar hemorrhage and reported alleviation of the symptoms^574)^. Relevant literature concerning function was scarce similarly to that on symptoms.

####### D．Cosmetics

Cosmetic improvements are difficult to evaluate objectively. Poonyathalang *et al.* administrated sodium tetradecyl sulfate to three patients with orbital lesions with exophthalmos as the primary symptom and reported improvement by measuring the degree of protrusion before and after the treatment^574)^. There have also been reports of objective assessment based on the degree of satisfaction in the patients’ families. According to Chaudry *et al.*, all patients with head and neck lesions (9 with microcystic lesions, 22 with mixed lesions) and their families reported improvements in the size and appearance of the lesions^566)^. Also, Alomari *et al.* treated 32 patients consisting partially of those with macrocystic LM but mostly of microcystic LM of head and neck region by sclerotherapy and reported that the condition was evaluated to have improved compared with that before treatment by the families of 26 (81.3%) patients^565)^.

###### II．Complications

As complications in the facial region, there are a large number of reports of transient complications associated with sclerotherapy, such as fever, local swelling and pain, intracystic hemorrhage and infection, although the lesions were poorly characterized in some reports^557,558,561,562,564,566,569,574-583)^. In addition, complications considered to have been caused by the effect of treatment, such as ulcer of the oral mucosa and tongue, facial nerve paralysis, Horner’s syndrome, leakage of saliva and respiratory insufficiency due to airway obstruction, have been occasionally reported^568,569,573,574)^. There have also been reports of an elevation of the intraorbital pressure, exophthalmos, intraorbital hemorrhage, corneal damage and external ocular muscle paralysis due to enlargement of the mass after sclerotherapy for ocular LM^574,584)^. In a systematic review and meta-analysis of 25 papers on sclerotherapy for head and neck LM, De Maria *et al*.^569)^ reported that the incidences of complications of sclerotherapy for microcystic LM of the head and neck region were 3.6% for pulmonary disorders, 24.9% for systemic complications (fever, metabolic acidosis, hemolytic anemia, hypotension), 1.1% for permanent complications (facial nerve paralysis, Horner’s syndrome), 2.6% for temporary local complications (swelling, inflammation, transient epidermal necrosis, hematoma, transient neuropathy, pain), and 0.8% for skin necrosis/scarring. According to sclerosing agents, in head and neck LM including macrocystic and mixed types, the incidence of permanent complications was high with doxycycline (5.9%) and monoethanolamine oleate (4.1%).

As complications caused by sclerosing agents, skin ulcer and necrosis and nerve damage due to ethanol leakage, hypotension during anhydrous ethanol injection, and epidermal detachment and topical hair loss due to doxycycline have been reported^557,565,585)^. However, complications caused by OK-432 and polidocanol foam were reportedly mild^578,582)^. Pulmonary fibrosis is widely known to be a complication of bleomycin, but, according to Chaudry *et al*.^566)^ and Yang *et al*.^564)^, impairment of respiratory function does not occur at a dose routinely employed for sclerotherapy.

Papers that analyzed only microcystic LM of the face are scarce, and many evaluated lesions of not only the face but also the neck and other areas or reported the results by including LM with different properties such as the macrocystic and mixed types. Also, in addition to this point, the definition of microcystic LM or therapeutic standard of sclerotherapy (method for the use of sclerosing agents, number of administrations) considerably varies, and differences in the background of patients must be taken into consideration in evaluating the effectiveness of sclerotherapy.

####### ■Radiofrequency ablation

Three papers on 43 cases were adopted as references concerning radiofrequency ablation for microcystic LM of the head and neck region.

Kim *et al.* performed radiofrequency ablation a total of 54 times in 22 patients (1–7 times/patient) with mucosal LM of the mouth (localized LM) that repeatedly bled or was infected for symptomatic alleviation, and management in the intensive care unit was necessary due to swelling of the tongue in 2^586)^.

Cho *et al.* evaluated ablation using insertion type and non-insertion type electrodes in 15 patients and reported that regression of the lesions on imaging examination and cosmetic improvements were achieved with no significant difference in the effectiveness. As complications, pain was reported with non-insertion type electrodes, and 1 case of House-Brackmann grade II facial nerve paralysis and 1 case of hypesthesia due to mental nerve injury were reported with injectable electrodes^587)^.

Obatake *et al*.^588)^ performed ablation in 2 patients with macroglossia protruding from the oral cavity and reported regression of the lesion in 1 but no regression resulting in glossectomy in 1.

Although radiofrequency ablation is performed for inducing regression of the lesion or alleviation of symptoms, all reports are insufficient in the number of cases and lacked comparison with other treatments, so the evidence is considered inadequate.

####### ■Drug therapy

Regarding the usefulness of drug therapy for microcystic LM of the head and neck region, 1 paper on sirolimus^589)^, 2 papers on sildenafil^590,591)^, and 1 paper on propranolol^592)^ were adopted.

Concerning sirolimus, it was reported that the blood drug concentration was controlled at 7–13 ng/mL in 19 patients, including 9 with mixed type LM, that regression of the lesion was observed in all patients, and that the treatment tended to be more effective in younger and previously untreated patients^589)^. Complications, which were observed in 17 patients, were mild ones such as cellulitis, stomatitis, eczema, vomiting, and intracystic hemorrhage.

Concerning the literature on sildenafil, there were 2 papers about 11 cases^590,591)^. Regression of the lesion was reported in some patients, but as the evaluation method or patient background was not uniform, and as the number of patients was small, they are not considered to be sufficient as evidence. In all papers, the blood concentration of sildenafil was controlled at 2–4 mg/kg/day. Regression of the lesion was observed in only 2 patients, and, in comparison with patients with macrocystic LM in the same report, the treatment was suggested to be less effective for microcystic LM. Mild diarrhea was reported as a complication.

Oral propranolol therapy was reported to be effective for inducing regression in some regions other than the head and neck region, but no regression was obtained in head and neck microcystic LM^592)^.

None of the oral drug therapies was compared with other treatments in any report, and evidence is considered to be insufficient for the selection of treatment.

^＊^Other than the above, lymphaticovenular anastomosis has recently been reported as a treatment option for LM^593)^. It is not widely accepted at present, and there are expectations for its future development.

###### <Conclusion>

In evaluating the CQ “What treatments are effective for facial (orbital/palpebral) microcystic LM?”, the therapeutic effects and complications of surgery, sclerotherapy, radiofrequency ablation, and drug therapy were analyzed, but most papers were case series or case reports. Concerning surgery, total resection was more difficult, and the treatment was suggested to be less radical, in microcystic than macrocystic LM. Comparison between surgery and sclerotherapy showed neither as superior. The degree of regression of the lesion by sclerotherapy varied among reports, but it was smaller in microcystic LM than in macrocystic LM. Although some papers referred to symptoms, functional outcome, and cosmetics, they were insufficient for general discussion of sclerotherapy against facial microcystic LM. Radiofrequency ablation has been reported to be performed for induction of lesion regression and alleviation of symptoms, but the evidence is insufficient at present. Regarding drug therapy, also, there is no report of its comparison with other treatments, and the literature was inadequate as evidence for the selection of treatment. On the basis of the above observations, it is presently difficult to determine indications for the treatment of facial microcystic LM by setting up some criteria. Therefore, validation of each treatment by research designs including RCT is considered necessary for the evaluation of this CQ.

##### 3．Evaluation of balance of benefits and risks

Although no report with a high evidence level was observed, surgery, sclerotherapy, radiofrequency ablation, and drug therapy were all effective and are likely to be beneficial. However, as the treatments may be ineffective, as there are many reports of complications, and as there is the possibility that risks surpass benefits, careful evaluation of indications in individual cases is needed.

##### 4．Patients’ values/wishes

While all treatments are in line with the patients’ wishes, there is the possibility of the occurrence of complications, particularly, in surgical treatment, and it is necessary to select treatments with sufficient understanding of the possibility of their negative effects.

##### 5．Cost assessment, assessment of external validity of intervention

Surgical resection and sclerotherapy are already recognized as treatments by coverage by National Health Insurance in Japan. Concerning drug therapy, oral sirolimus was approved as a treatment for refractory lymphatic disorders in late September 2021, but its cost has not been sufficiently evaluated, and the future announcement is awaited. Radiofrequency ablation is presently not covered by National Health Insurance in Japan, and its validity must be carefully evaluated in individual cases.

#### Lay summary

Facial (including orbital and palpebral) microcystic LM has been treated by surgery, sclerotherapy, radiofrequency ablation, or drug therapy. Microcystic LM is more difficult to totally resect or be reduced in size by either surgery or sclerotherapy than macrocystic LM, and which treatment is more effective remains uncertain. Radiofrequency ablation and drug therapy may be performed for inducing regression of the lesion or alleviation of symptoms, but their effectiveness has not been sufficiently demonstrated to the present. Because of the absence of criteria for the evaluation of indications or treatment methods, it is necessary to select treatments according to the condition and wishes of each patient.

### CQ29: What treatments are effective for intra-abdominal LM?

Recommendation: Surgery and sclerotherapy are relatively common treatments, but each of them has the risk of complications, and their relative advantage is not clear. Treatment must be selected carefully in consideration of the condition of each patient including the site and size of the lesion.

Strength of recommendation: 2 (weak)

Evidence: D (very weak)

#### Process of preparation of recommendation

##### 1．Circumstances that make CQ an important clinical issue

Abdominal lesions are estimated to account for 10–20% of all lymphatic malformation (LM), and the selection of treatment is difficult depending on the spatial relationship between the lesion and organs, particularly, in the abdomen, which contains important organs. While surgical resection is expected to be effective, less invasive treatments are considered desirable in view of intra-/postoperative stress to the patient and the possibility of severe complications such as lymphatic fluid leakage and bowel obstruction. Sclerotherapy is also expected to be effective to an extent, but it poses important questions, such as whether it can be performed safety without complications and how effective it is over a long period, because it causes severe inflammation. Recently, also, other approaches including pharmacologic treatments have emerged as possible options. It is extremely important to select appropriate treatments from these options. The latest information concerning this point is summarized.

##### 2．Evaluation of evidence

###### <Literature search>

As a result of systematic literature search, 270 papers in Japanese and 155 papers in European languages were subjected to primary screening. As a result, 20 papers in Japanese and 25 papers in European languages were subjected to secondary screening. Of these papers, 2 in Japanese and 16 in European languages had contents corresponding to this CQ, but they were all case series or case reports and did not include RCT. The results and discussion in the references judged to be useful for the preparation of the recommendation statement were integrated and presented as review data, although their evidence level is low.

###### <Evaluation>

The effectiveness of each treatment for abdominal LM was evaluated from the following considerations.

I．Treatment responses

A．Size

B．Symptoms

II．Complications

The contents of descriptions about the effectiveness of each treatment for abdominal LM were summarized from these considerations.

However, sclerotherapy was performed before, after or during surgical resection in many reports, and reports of the results of sclerotherapy alone were few. There was no paper that directly compared observation without treatment, sclerotherapy and surgical resection. There were only case reports concerning pharmacologic treatments such as sirolimus.

Few papers analyzed intra-abdominal lesions alone, and many papers included lesions in other areas or collectively evaluated lesions in different intra-abdominal regions including the mesentery, retroperitoneum and viscera. The literature in which exclusive evaluation of abdominal lesions was impossible was excluded.

In addition, the type of LM, such as macrocystic, microcystic, and mixed types, their definitions, criteria for treatments (indications of surgery and sclerotherapy, types and methods of the drugs used for sclerotherapy, and number of their administrations), and time of treatment varied among papers, and few of them made distinctions by these factors in the evaluation.

As a result of a review in consideration of the above points, abdominal LM was treated by surgery, drainage by puncture, sclerotherapy (OK-432, bleomycin), or pharmacologic treatments (Kampo formulas) or was occasionally observed without treatment. Of these treatments, surgical resection and sclerotherapy were evaluated in a relatively large number of cases, and the number of cases in reports about other treatments was extremely small, e.g., reports of a single case. Therefore, the information of case series primarily of surgical resection and sclerotherapy is summarized as follows.

Treatments are discussed individually in the following sections.

####### ■Surgical treatment

Reports of surgical treatments consisted mostly of those about resection of lesions located in the mesentery, retroperitoneum, and greater and lesser omentum^594-606)^. There were also sporadic reports of surgical resection of lesions in solid organs such as the spleen^599,604)^ and adrenal gland^597,599)^. Few reports that evaluated differences in the therapeutic effect or complications according to the patient background or surgical procedure were observed in the present review of the literature. Therefore, differences in the patient background and contents of treatment must be considered in evaluating the effectiveness of surgical treatments.

###### I．Treatment responses

####### A．Size

Surgical treatment was complete resection in many cases^594-606)^. Therefore, the lesions disappeared in most cases, but the postoperative follow-up period varied from a few months to about 10 years, and attention to this point is necessary^597,598,600,602-606)^.

There have been reports of combined resection of the lesion with surrounding organs. Kim *et al.* reported that complete resection could be performed in 24 cases with mesenteric lesions but that it was combined with intestinal resection in 21 cases because the lesions involved the intestinal wall or was close to the feeding vessel^596)^. Tran *et al.* performed laparoscopic surgery in 47 cases of macrocystic LM (derived from the mesentery in 35, from the greater or lesser omentum in 12) and obtained disappearance of the lesion in 46, but reported that combined intestinal resection was performed in 8 of them^603)^. Zhou *et al.* reported that splenectomy was performed in all 7 cases with splenic lesions with a favorable postoperative course^604)^.

Evaluation was also made with regard to recurrence after surgical resection. Recurrence was observed in 5 (2.7%) of a total of 188 cases in the 13 case series reports retrieved^597,599,603)^. All these recurrent cases had undergone partial resection, reportedly because total resection was impossible due to extensive adhesion of the lesion to the surrounding organs.

Regarding the extent of resection, there were opinions that combined resection of tissues around the lesion is recommended if perilesional infiltration is suspected with respect to recurrence and complications^597)^ and that combined intestinal resection may be unavoidable in patients with mesenteric lesions^601)^, but there are also reports that whether complete resection of the lesion even with concomitant resection of important organs cab be recommended is controversial^598)^, and the question remains open.

####### B．Symptoms

Symptoms are diverse and include abdominal pain, ileus, torsion, infection, hemorrhage, vomiting/suckling disorder, urination disorder, abdominal mass, and fever^595,596,599-603,605,606)^. Their occurrence is considered to depend on the site and size of the lesion and age of the patient.

As mentioned above, since many patients included in case series had undergone complete resection, symptomatic improvements are considered to have been obtained in most of them, but there were few reports that clearly stated “symptoms were resolved”. However, Chiappinelli *et al.* observed abdominal pain in 7 and abdominal mass in 3 of the 14 cases of LM involving the mesentery or intestine and reported that the symptoms were alleviated in all cases after surgery^595)^.

###### II．Complications

There have been sporadic reports of complications of surgical resection, such as ileus^596,600,606)^, ascites^596,606)^, and wound infection^606)^, and 1 case of pancreatic fistula was also reported^598)^. As for serious complications, there was a report of massive intestinal resection necessitated by inferior vena cava obstruction or aberration of LM into the intestinal wall^594)^. Chylous ascites was often observed in cases of partial resection^596,606)^.

Regarding complications of complete laparoscopic resection, Tran *et al.* reported that laparoscopic procedure was switched to open surgery in 3 (6.4%) of the 47 abdominal LM cases due to tight adhesion in 2 and intraoperative hemorrhage in 1^603)^.

####### ■Sclerotherapy

Drugs used for sclerotherapy varied widely and included OK-432, bleomycin, ethanol, doxycycline, sodium tetradecyl sulfate, acetic acid, steroid/tetracycline, and 50% glucose solution. There was no paper that evaluated differences in effectiveness of sclerotherapy for abdominal LM according to the drug used, administration method of each drug, or number of administrations.

Such differences in the patient background and contents of treatment must be considered in evaluating the effectiveness of sclerotherapy. This CQ was evaluated without particular consideration of differences in the shape of LM or the drug used for sclerotherapy.

###### I．Treatment responses

####### A．Size

There were 7 papers that referred to regression of abdominal LM by sclerotherapy^601,607-612)^. There were multiple reports of case series treated with doxycycline. According to the report by Chaudry *et al*.^608)^, 90% or greater regression was observed in 7, and 20% or greater regression was observed in 1, of the 10 patients with mesenteric or retroperitoneal LM. Evaluation by imaging examination was not made in 2. The lesions were mixed type LM in the case with a small regression rate and were macrocystic LM in the other cases. In the report by Madsen *et al*.^609)^, also, complete resolution of the lesion was achieved in 5, and 80% or greater regression was observed in 2, of the 10 treated cases of mesenteric or retroperitoneal LM. Evaluation by imaging examination was not made in 2, and additional surgery was performed in 1 despite regression. According to the report by Russell *et al*.^610)^, regression was observed in all 5 cases of retroperitoneal LM (median regression rate: 62%).

As for reports concerning other drugs, Oliveira *et al*.^601)^ reported that 70% regression was achieved in 1 of the 2 cases of macrocystic LM treated with OK-432. Won *et al*.^612)^ reported complete resolution of the lesion in 1 case of retroperitoneal macrocystic LM by treatment with acetic acid. Shiels *et al*.^611)^ reported that sclerotherapy using sodium tetradecyl sulfate and ethanol was effective in 2 cases of macrocystic LM, but they did not mention the regression rate. However, according to Alqahtani *et al*.^607)^, none of the 10 cases treated with steroid/tetracycline or 50% glucose solution responded to the treatment.

####### B．Symptoms

There were 4 papers that referred to symptoms in cases that underwent sclerotherapy for abdominal LM^601,608-610)^. Chaudry *et al*.^608)^ reported that, of the 10 cases that underwent sclerotherapy, 3 complained of chronic abdominal pain, 3 had acute abdominal pain, 1 had fever/chill, 1 had anemia, and 2 had palpable masses but that the symptoms were alleviated in all cases after treatment without reactivation.

In the report by Madsen *et al*.^609)^, symptoms were observed in 8 of the 10 treated cases of mesenteric or retroperitoneal LM, and the symptoms, which were abdominal pain in 5, abdominal distension in 5, constipation in 2, infection in 2, anorexia in 2, and nausea/vomiting in 1, were alleviated in all patients.

Russell *et al*.^610)^ reported that 4 of the 5 cases of retroperitoneal LM had symptoms, which were fever in 3, nausea/vomiting in 3, abdominal distension in 2, and abdominal pain in 1, and that all symptoms were resolved as a result of treatment.

Oliveira *et al*.^601)^ treated 1 case of palpable mass and 1 case of palpable mass and abdominal compartment syndrome/poor general condition by sclerotherapy. The symptom was relieved in the case with palpable mass alone after 2 times of OK-432 sclerotherapy, but the treatment was switched to surgery in the case of abdominal compartment syndrome because of enlargement of the mass due to intracystic hemorrhage.

###### II．Complications

Three papers mentioned complications of sclerotherapy for abdominal LM in specific terms. There was no report of death caused by complications of treatment.

Oliveira *et al*.^601)^ reported that, of the 3 cases that underwent OK-432 sclerotherapy, 1 developed subileus after treatment, and 1 showed exacerbation of abdominal compartment syndrome due to intracystic hemorrhage and required emergency surgery. Chaudry *et al*.^608)^ reported that the drug leaked into the retroperitoneal cavity in 1 of the 10 cases that underwent doxycycline sclerotherapy but that the lesion was resolved without particular problems.

According to the report by Madsen *et al*.^609)^, extracystic leakage of the drug was observed in 1 of the 10 cases treated by doxycycline sclerotherapy, but the condition was relieved by conservative treatment. Also, while infection was noted in 1, it could be managed by antibiotic therapy.

Won *et al*.^612)^ performed sclerotherapy using acetic acid in 1 case of retroperitoneal macrocystic LM, and although the patient suffered pain and hematuria, they concluded that the relationship of hematuria with the treatment was unclear because of the concurrence of menstruation.

###### <Conclusion>

In evaluating the CQ “What treatments are effective for intra-abdominal LM?”, the literature was reviewed for its treatments, but no reference with a high evidence level could be found.

Reports on surgical treatments were largest in number, followed by those on sclerotherapy. There was no literature that compared surgical treatment with sclerotherapy or other treatments, and their relative effectiveness could not be evaluated, but many papers suggested that surgery is effective for reducing the lesion size (eliminating the lesion) if complete resection is possible. On the other hand, complete resection is difficult in extensive lesions invading areas outside the primary lesion, and there was a tendency of higher incidences of recurrence and complications. Regarding the issue of whether adjacent organs should be resected with the primary lesion, there were sporadic reports of combined resections including partial intestinal resection if the lesion involved the intestine, but the information was insufficient for generalization.

Also, regarding sclerotherapy, sufficient regression of the lesion or improvements in symptoms were obtained in some cases, but the response rate varied among reports, and the literature was insufficient for general discussion of sclerotherapy. Also, the effectiveness of sclerotherapy varied to an extent according to the disease type, and there were reports that it was less effective in microcystic or mixed LM compared with macrocystic LM. Ileus was also reported as a complication of sclerotherapy, and attention to ileus as well as to intracystic hemorrhage is considered necessary. However, chylous ascites, which was reported in surgery, was not reported as a complication of sclerotherapy.

To summarize the above, while there were some reports evaluating the effectiveness of each treatment, it is difficult to discuss its effectiveness or safety based on actual numbers, and future validation of this CQ by study designs, such as RCT, is considered necessary.

##### 3．Evaluation of balance of benefits and risks

Surgical resection and sclerotherapy are expected to be more or less effective and are clearly beneficial, but their indications vary depending on the condition of individual lesions. At the same time, they have been shown to possibly cause serious complications, and sufficient consideration of their risks is also necessary. With appropriate selection of treatments, benefits are considered greater than risks.

##### 4．Patients’ values/wishes

Implementation of the treatments discussed here for abdominal LM conforms with the patients’ values/wishes, particularly when there are severe symptoms or when there is a tendency of exacerbation. However, symptoms are occasionally unclear, in which case, the patients’ values or wishes about the necessity of treatment are not uniform.

##### 5．Cost assessment, assessment of external validity of intervention

All treatments are approved to be covered by National Health Insurance in Japan, and the costs of treatments performed as necessary are considered reasonable compared with their effects.

#### Lay summary

Treatments for abdominal LM are typically surgical resection and sclerotherapy, and there are not many reports on other treatments. All treatments are generally considered effective, but the possibility of complications has also been reported. Benefits and risks of each treatment vary depending on the site of the lesion relative to important organs, its size, and symptoms. Treatment is evaluated positively if a tendency of enlargement of the lesion is observed, or if there are symptoms, such as pain, but it is necessary to carefully select appropriate treatments according to the condition of each patient.

### CQ30: What treatments are effective for refractory chylous ascites?

Recommendation: Conservative approaches, such as fasting, parenteral nutrition, and medium-chain fatty acids, and pharmacologic treatments, such as octreotide, are attempted first. If they are ineffective, treatments, such as sclerotherapy, surgery, and lymph vessel embolization, are considered. The effectiveness of all these treatments is uncertain, and they are used in combinations as necessary.

Strength of recommendation: 2 (weak)

Evidence: D (very weak)

#### Process of preparation of recommendation

##### 1．Circumstances that make CQ an important clinical issue

Refractory chylous ascites causes loss of large amounts of protein and lymphocytes, decreases in the blood lipid levels, and abdominal pain, unpleasantness and dyspnea due to abdominal distention, and markedly reduces the patients’ quality of life, occasionally affecting their survival. The cause of ascites varies widely, and often remains unknown. Treatment of chylous ascites may require continuous drainage or periodic drainage by puncture to avoid abdominal distention, and weaning from drainage is often difficult. It is a very important point for clinicians to make proper judgments by understanding treatments and their effects and demerits, so a summary of the up-to-date knowledge is presented as a guidance.

##### 2．Evaluation of evidence

###### <Literature search>

By systematic literature search, 758 papers in European languages and 298 papers in Japanese were retrieved. As a result of their primary screening, 25 papers in European languages and 13 papers in Japanese were subjected to secondary screening, and, eventually, 24 papers in European languages and 12 papers in Japanese were adopted as references for this CQ. They consisted entirely of retrospective case reports or case series reports, and none was an interventional study or RCT to verify the effectiveness of a particular treatment. Moreover, 84 papers in European languages were retrieved by hand search, and 2 papers on prospective interventional studies and 1 protocol paper of prospective interventional studies were added to the references. The results and discussions in these papers were integrated in the evaluation of the recommendation statement for this CQ. The 39 references judged to be useful for the preparation of the recommendation statement are presented as review data.

###### <Evaluation>

No reference defined refractory chylous ascites according to the duration of illness or treatment response. As for causes of chylous ascites, congenital chylous ascites^613-633)^, idiopathic chylous ascites^614)^, chylous ascites after laparotomy^634-637)^, chylous ascites related to heart disease^638,639)^, protein-losing enteropathy^636)^, LM^640,641)^, lymphangiectasis^642,643)^, lymphangiomatosis^638,644-646)^ and lymphatic dysplasia^647)^ were reported, but none of the papers evaluated treatments according to the cause. Conservative treatments (fasting, high-calorie infusion, medium-chain triglyceride), pharmacologic treatments, sclerotherapy, lymphangiography, lymphatic embolization, and surgical treatment were performed^648)^. Each treatment was evaluated.

####### ■Conservative treatments

Fasting is considered the first-line treatment, because it may reduce ascites.

Parenteral nutrition (high-calorie infusion) is often used with fasting, and because there was no report that ascites increased under the effect of high-calorie infusion, it is recommended to be performed concomitantly for nutritional support during fasting. In a multicenter case series study of 15 cases, no adverse reaction to high-calorie infusion/total parenteral nutrition was observed^613)^.

Medium-chain triglyceride is used in all phases of treatment^613,614,616,618-621,623,625,626,628,634,636,637,641-643,645,646)^. In a multicenter case series study of 14 cases, no adverse reaction to medium-chain triglyceride was noted^613)^.

####### ■Pharmacologic treatments

Concerning drug therapy, multiple reports concerning octreotide (long-acting somatostatin analogue preparation) were found. In a multicenter case series, octreotide was administrated to 6 of the 16 patients with chylous ascites for 8–38 days, and a decrease in ascites was reported in all of them^613)^. In the single-center case series, 2 of the 4 patients with chylous ascites treated by high-calorie infusion and octreotide administration were reported to have shown a decrease in ascites within 10 days^635)^. However, there has been a report that no effect was observed despite the administration of octreotide for 3 weeks^616)^. Concerning the dose of octreotide, it was administrated at 1–7 μg/kg/h^613)^, at 3 μg/kg/h^618)^, started at 0.5 μg/kg/h and increased to 10 μg/kg/h by 1 μg/kg/h^615)^, administrated by continuous i.v. infusion at 0.5–2.0 μg/kg/h^619)^, and began to be administrated by s.c. injection at 2.5 μg/kg 2 times a day and increased every 2 days to 8 μg/kg 2 times a day^616)^. Zaki *et al*.^632)^ started the administration of octreotide at 1 μg/kg/h to 11 neonates with chylous ascites (administration started 30–47 weeks gestation) and increased the dose to 10 μg/kg/h, with 4 patients responding to the treatment. Although no serious adverse event was noted^632)^, as there have been sporadic reports of necrotizing enteritis primarily in premature infants with chylous ascites, careful observation is necessary in the use of octreotide in neonates. Regarding the time of the beginning of octreotide administration, there were reports that the administration was started because chylous ascites showed no improvement for 2 weeks by conservative treatments^616,620)^, and because it was exacerbated again after temporary alleviation by conservative treatments^619)^. There was no report of adverse reaction to octreotide administration.

Validation of the effectiveness of mTOR inhibitors as a drug therapy for vascular malformations is underway. A prospective clinical study of the effectiveness of sirolimus against various vascular malformations reported that the treatment was effective in 47 of the 57 cases 6 months after the beginning of the administration^649)^, but the treatment effects in 13 cases with effusion (sites unknown) and 2 with ascites were unknown. In a prospective clinical study in 13 cases of generalized lymphatic anomaly and 5 cases of Gorham-Stout disease, Ricci *et al.* reported that clinical improvements were observed in 15 and that ascites was reduced in 5 of the 7 cases with ascites^650)^. A case in which intraperitoneal lesion showed marked regression was presented, but there was no mention about ascites as a complication. Ozeki *et al.* performed a prospective clinical study of sirolimus in patients with vascular malformations, but they did not aim to examine its therapeutic effect on chylous ascites^651)^. Although there have also been a few reports that sirolimus was effective for ascites complicating lymphangioleiomyomatosis, this disorder was not included in the targets of the present evaluation.

####### ■Lymphangiography, lymphatic embolization, lymphaticovenous anastomosis

Techniques of visualization and treatment of lymphorrhea are known to have improved. Imaging techniques, such as lymphoscintigraphy, SPECT, and MR lymphangiography, are used for the delineation of intraperitoneal lymphatic channels, and methods, such as infusion of a contrast agent into the inguinal lymph node, are used for radiographic examination of lymphatic channels.

Regarding lymphatic embolization, lymphangiography primarily using lipiodol as a contrast agent and selective treatment of lymphatic vessels using a microcatheter under fluoroscopic guidance (interventional radiology) have been reported. In chylous ascites due to leakage from the thoracic duct and its collaterals, lymphorrhea can be controlled by embolizing the upstream side of the site of leakage, and there have been reports of this procedure applied to children^639)^. However, in chylous ascites, lymph may leak at multiple sites including the mesentery and retroperitoneum, and the sites of leakage are often difficult to determine, which markedly restricts the usefulness of embolization. Since lipiodol is highly viscous, imaging using lipiodol itself may have an embolizing effect, and it may exacerbate clinical symptoms due to occlusion of unintended lymphatic channels despite its possible benefit as a treatment. Therefore, imaging using lipiodol must be performed with sufficient prior evaluation and experience.

Visualization of lymphatic channels is widening the potential of lymphatic microsurgery. Lymphaticovenular anastomosis, which bypasses the lymph flow into the site of lymphatic leakage to a vein, has been reported. Chen *et al.* performed chylovenous bypass by anastomosing the lesion of retroperitoneal LM to the end of a vein in 6 adults with retroperitoneal LM accompanied by ascites and lower limb edema and reported satisfactory results^644)^. Kato *et al.* reported the usefulness of lymphangiography and lymphovenous anastomosis with the therapeutic strategy in 12 children with chylous ascites^638)^.

####### ■Sclerotherapy

According to the present review of the literature, sclerotherapy was performed in 6 patients in 5 case reports^625,640,643,645,646)^. The sclerosing agent was OK-432 in 5 of the 6 patients and povidone-iodine in only 1^643)^. OK-432 was injected locally into the lesion in 4^640,645,646)^, administered intraperitoneally in 1^646)^, and administered via the drain in 2^640,646)^.

####### ■Abdominal drainage, abdominal puncture and surgical treatment

Abdominal drainage and abdominal puncture are performed when organ compression symptoms (compartment syndrome and respiratory insufficiency) due to abdominal distention are present or possible, or when the drain is inserted postoperatively, but these procedures per se are not treatments for chylous ascites. They do not relieve ascites, and treatments, such as infusion, blood preparations and blood transfusion, are necessary to supplement the ascites lost due to drainage^613,616-619,623-626,634,636,637,640,643,645,646)^.

Surgical treatment is reported to be frequently performed after conservative or pharmacologic treatments. According to the single-center case series by Zeidan *et al*.^634)^, surgical treatment was performed in patients who responded poorly to conservative treatments sustained for over a mean of 25.3 days. In other reports, surgical treatment was performed after conservative treatments for 1–3 months^614,615)^ and in patients with congenital chylous ascites 1–4 months after birth^616,620,642)^. Because it is often impossible to identify the leakage site of chylous ascites^616)^, there have been attempts to identify the leakage site by p.o. administration of a lipophilic dye (Sudan black, Sudan III) before operation^614,615,622,634)^. When the leakage site can be identified, ligation, suturing, clipping and cauterization have been performed^614,620,622,634,642)^. An attempt to identify the leakage site by subserous injection of a suspension of carbon microparticles (India ink) has been reported^630)^. Techniques, such as applying or sprinkling fibrin glue to the leakage site of chylous ascites or over the surrounding retroperitoneum^615,617,634,642)^, applying a patch of oxidized cellulose/resorbable local hemostatic agent^617,634)^, and applying VICRYL mesh, have been reported to be useful for stopping leakage^631,643)^. There have also been reports of treatment of fetal cases by peritoneovenous shunting^643,647)^ and peritoneoamniotic shunting^624)^.

###### <Conclusion>

No case-control study existed concerning any of these treatments, but many case reports and case series reports were evaluated although their evidence level of the references is low.

Conservative treatments, such as fasting, parenteral nutrition, and medium-chain triglyceride, are considered to worth attempting first due to less adverse reactions, although the evaluation of the effect of each treatment is scarce. Also, if conservative treatments are ineffective, octreotide may be worth considering as pharmacologic treatments. Recently, sirolimus has been reported to be effective as a treatment of refractory lymphatic disorders, but there is no evidence that supports its effectiveness as a treatment for associated chylous ascites, and further validation is needed. If no response is obtained by conservative/pharmacologic treatments for about 1 month, it may be reasonable to consider surgical treatment, but there are refractory cases, and the effectiveness of surgery has not been established. Concerning sclerotherapy, further accumulation of cases is considered necessary to demonstrate its usefulness. Among surgical procedures, it must be noted that abdominal cavity drainage and abdominal puncture are performed to control symptoms and not as direct treatments for ascites.

##### 3．Evaluation of balance of benefits and risks

While benefits of conservative treatments and octreotide are uncertain, their risks are small, and there is no reason for hesitation of their implementation. Benefits of sclerotherapy and surgical treatments are also uncertain, but as their risks are considerable, careful evaluation is considered necessary in their implementation.

##### 4．Patients’ values/wishes

There is no reliably effective treatment for refractory chylous ascites, and its treatment is prolonged. It is inevitable to first attempt symptomatic control by conservative and pharmacologic treatments. If they are ineffective, the selection of more invasive sclerotherapy or surgical procedures is unavoidable although there is a risk of complications, and they are considered to conform with the patients’ values/wishes.

##### 5．Cost assessment, assessment of external validity of intervention

Each of the treatments must be selected without reliable evidence. Octreotide is a relatively expensive drug, but the selection of treatments in the order shown above is considered reasonable in view of the invasiveness of intervention.

#### Lay summary

Chylous ascites is often unexplained and difficult to treat. If abdominal distension is severe, drainage of ascites by abdominal puncture/drainage is necessary, but weaning from these procedures is often difficult. For its treatment, priority is given to conservative approaches with less stress to the body such as fasting, parenteral nutrition, and medium chain fatty acids. If responses to these treatments are insufficient, pharmacologic treatment using drugs including octreotide is attempted. If the condition still fails to respond, treatments, such as sclerotherapy to induce occlusion of fistula by adhesion and surgery to directly close the leakage site, may be considered, but they involve a large burden to the body and may cause complications. Recently, there have been reports of lymphatic embolization and lymphovenous anastomosis, but the evidence concerning their effectiveness remains insufficient. The effectiveness of each treatment is uncertain, and each treatment is combined with other treatments as needed.

### CQ31: What treatments are effective for LM causing airway obstruction in the mediastinum?

Recommendation: Both surgical resection and sclerotherapy are effective for reducing the lesion size and relieving symptoms, but they may cause complications. Their relative superiority is unclear, and they are often performed in combination, but the treatment should be selected according the condition of each case.

Strength of recommendation: 2 (weak)

Evidence: D (very weak)

#### Process of preparation of recommendation

##### 1．Circumstances that make CQ an important clinical issue

Among LM, some of those that develop around the airway cause airway stenosis, which may be life-threatening. Particularly, mediastinal LM occasionally causes airway stenosis and respiratory disorders by compressing the trachea and bronchi. Such cases need treatment, but the selection of effective and safe treatment appropriate for each case is often difficult.

##### 2．Evaluation of evidence

###### <Literature search>

As a result of a literature search, 113 papers in Japanese and 235 papers in European languages were retrieved and subjected to primary screening. Consequently, 20 papers in Japanese and 93 papers in European languages were subjected to secondary screening. They included 1 RCT but many of the rest were case series or case reports.

However, mediastinal LM that causes airway stenosis was not analyzed as a direct target of RCT or case series studies and accounted mostly for part of the cases in case series and case reports. Eventually, the results and discussions in the case reports judged to be useful for the preparation of recommendation statement (3 in Japanese, 20 in European languages) are presented as review data although they are inadequate as evidence.

###### <Evaluation>

From the literature, information concerning the effectiveness of various treatments for mediastinal LM was summarized. Many of the reports were about cases treated by combinations of several treatments, and papers that analyzed the outcomes of a single treatment, particularly sclerotherapy, were few. Also, papers that analyzed mediastinal lesions alone were scarce, and many analyzed lesions including those in other areas. In some reports, mediastinal lesions were evaluated without distinguishing the affected areas such as the anterior and superior mediastinum. Disease types such as macrocystic, microcystic, and mixed types, their definitions, treatment criteria (indications for surgery and sclerotherapy, types and use of sclerotherapy drugs, number of administrations, etc.), and time of treatment varied among papers, and few papers classified lesions or treatments in evaluation.

As a result of evaluation on the basis of the above points, mediastinal LM was treated by surgery, drainage by puncture, sclerotherapy (OK-432, bleomycin, doxycycline, Ethibloc, anhydrous ethanol), and pharmacologic treatments (Kampo formulas, steroids), and it was occasionally observed without treatment. Relatively many cases were treated by surgery and sclerotherapy, and there were only a few reports on pharmacologic treatments such as Kampo formulas and steroids.

####### ■Surgical treatment

The effectiveness of surgical resection for mediastinal LM was evaluated from the viewpoints of therapeutic effects (regression rate of the lesion, symptoms) and complications. However, there were few papers that evaluated the differences in the therapeutic effects or complications according to the patient background or surgical procedure.

###### I．Treatment responses

There were reports of 58 cases concerning surgical resection of mediastinal LM^573,652-670)^, of which surgical resection of lesions that caused airway stenosis could be confirmed in 14 cases^652,654-660)^. The disease types of these cases were macrocystic and mixed types. Also, many of such patients underwent repeated resections or resection in combination with sclerotherapy^652,654-657,660)^. Regarding the extent of resection, the lesion was eventually resected totally (including subtotal resection), and no recurrence was observed after resection, in 10 of the 14 cases. However, the postoperative follow-up period varied widely from a few months to about 10 years, and attention to this point is necessary.

Symptoms of airway stenosis were reported to have been relieved or disappeared after surgical resection in 12 of the 14 cases^652,654-659)^. However, some patients developed airway stenosis due to re-enlargement of the lesion after partial resection or bleeding or infection from the residual lesion^654,660)^.

Park *et al.* reported that they surgically resected mediastinal LM in 12 patients^668)^. Seven of them had dyspnea and 3 were asymptomatic, but they were all judged to have indications for surgery due to symptoms or the tendency of the lesions to enlarge. A total of 5 recurrences were observed in 4 patients (33%) during a mean period of 3.6 years after the initial surgery, and all underwent by re-resection. No perioperative death was observed, and, in a total of 25 cases including past cases, the overall survival was not different compared with that in healthy individuals over a follow-up period of 11.5 years.

###### II．Complications

Complications of surgical resection of LM that caused airway stenosis in the mediastinum included 2 cases of phrenic nerve injury, 4 cases of lymphorrhea (chylothorax), and 1 case of mediastinitis^652,657,659)^.

Boardman *et al*. did not mention airway stenosis but reported that surgical treatments were necessary in 6 of the 12 patients including those with mediastinal lesions, that 4 developed complications due to surgery, and that 3 of them developed chronic neural disorders^670)^. Also, 15% of all patients reportedly required management by tracheostomy. They observed complete or nearly complete remission in 92% of the cases but maintained that surgical resection of mediastinal lesions should be considered only when they are causing or are likely to cause airway stenosis, because it frequently leads to complications.

####### ■Sclerotherapy

Drug used for sclerotherapy ranged widely and included OK-432, bleomycin, doxycycline, Ethibloc, anhydrous ethanol, and 1% polidocanol. No paper evaluated differences in the effectiveness of drugs used or the administration method or number of administrations of each drug concerning sclerotherapy for mediastinal LM. Therefore, in evaluating this CQ, no distinction was made by the disease type of LM or the drug used for sclerotherapy.

###### I．Treatment responses

There were 6 reports concerning 15 cases of LM that caused airway stenosis in the mediastinum^573,652,654,656,660,671)^. Most of them were case reports, and OK-432 was the drug used most frequently.

Sclerotherapy was performed using OK-432 in 11 cases. Since it was performed in combination with surgical resection in most of them, it was difficult to discuss the therapeutic effect of sclerotherapy alone, but regression was noted in 5, and alleviation of symptoms was reported in 5^573,652,654,656,660)^. However, the degree of regression was not clearly described.

There were reports of 2 cases of sclerotherapy using bleomycin, but there was no mention about regression of the lesion or alleviation of symptoms^660,661)^.

Only 2 cases of sclerotherapy using doxycycline were reported^652,654)^. In both cases, sclerotherapy was performed before and after surgery, and the effect of sclerotherapy alone was difficult to evaluate.

Although airway stenosis was not mentioned, Smith *et al.* topically injected OK-432 in 16 cases of mediastinal LM and reported 60% or greater regression in 13 cases (81%)^567)^. They also referred to treatment responses of different disease types, reported that the response rate (complete or nearly complete remission) was 94% in macrocystic LM, 63% in mixed LM, and 0% in microcystic LM, and suggested that macrocystic LM is a good indication for sclerotherapy using OK-432. They considered that OK-432 sclerotherapy is more effective than surgical resection and is less prone to severe complications, based also on a review of the previous literature although not from the viewpoint of airway stenosis.

Also, Usui *et al.* reported endoscopic local injection of bleomycin in 11 cases of LM observed around the airway while not mentioning mediastinal lesions^672)^. The number of injections varied from 1–4 among the cases, and the regression rate was unknown, but regression was observed in all cases, and all 10 symptomatic patients were weaned from respiratory support or tracheostomy without serious treatment-related complications.

###### II．Complications

There were several reports that caution is needed in performing sclerotherapy for lesions around the airway using OK-432 or bleomycin, because airway stenosis occurred frequently due to swelling around the lesion after treatment^573,654,671)^.

####### ■Securing the airway

Of the 19 patients that underwent surgery or sclerotherapy for LM that caused airway stenosis in the mediastinum, all patients needed tracheostomy or endotracheal intubation before treatment or during repeated treatments^652,654-660,669)^.

Ueno *et al.* discussed the treatments and their results in mediastinal LM by a national survey^653)^. Of the 84 patients, the lesions were in contact with the airway in all the 20 patients who needed tracheostomy. However, concerning the 64 patients who did not need tracheostomy, the lesions were in contact with the trachea in 34 (about 55%). They observed that the necessity of tracheostomy tends to increase with widening of the area of contact of the mediastinal lesion with the airway and suggested that early securing of the airway by tracheostomy or by EXIT (ex utero intrapartum treatment) under fetal circulation during delivery by Caesarean section should be considered if there is the risk of airway stenosis. However, Ghaffarpour *et al.* reported that caution is necessary, because tracheostomy may not be effective in patients with a lesion compressing the airway distal to the tracheal orifice^654)^.

###### <Conclusion>

Although we evaluated the CQ “What treatments are effective for LM causing airway stenosis in the mediastinum?”, there was no paper with a high evidence level. The literature was limited to reports of surgical resection or sclerotherapy in small numbers of cases, and many of the cases were treated by their combination. Also, no paper compared surgery and sclerotherapy or other treatments individually, and their relative advantages or disadvantages are unclear.

Concerning surgical resection, there were relatively many case reports on total or subtotal resection, which resulted in reduction of the lesion size or symptomatic relief, but there were some reports of severe complications. However, concerning sclerotherapy, various drugs, among which OK-432 was notable, were used, but the literature was limited to case reports. Sclerotherapy was effective for reducing the lesion size in some reports but occasionally promoted airway stenosis after treatment if there was a mediastinal lesion around the airway.

In addition, it is necessary to pay attention to the appearance of respiratory disturbances before and after these treatments and to constantly evaluate indications for securing the airway by intratracheal intubation or tracheostomy. However, caution is needed, compression of the distal airway occurs in some patients with mediastinal lesions.

As observed above, either surgical resection or sclerotherapy or their combination was selected for the treatment of mediastinal LM, but the advantages and disadvantages of these and other treatments are unclear, and treatments were selected based on the condition of each case. The effectiveness or safety of each treatment is difficult to show on the basis of specific figures, and it is considered necessary for the future to evaluate this CQ by research designs such as RCT.

##### 3．Evaluation of balance of benefits and risks

While the relative superiority of surgery and sclerotherapy is unclear, each is effective for relieving symptoms. The effectiveness of sclerotherapy is considered to be limited for microcystic LM. Regarding complications, surgery is likely to have more risks than sclerotherapy.

##### 4．Patients’ values/wishes

Symptomatic relief and reduction of the lesion size by surgery or sclerotherapy, which makes resection possible, conform with the patients’ wishes. Unless complications of surgical treatments or sclerotherapy jeopardize the patients’ lives or markedly decline their QOL, surgical treatment and sclerotherapy for mediastinal LM are considered to conform with the patients’ values/wishes.

##### 5．Cost assessment, assessment of external validity of intervention

Surgical resection is already established as a treatment approved to be covered by National Health Insurance in Japan for LM. Although sclerotherapy is also approved as a treatment covered by National Health Insurance in Japan, it is coupled with the use of OK-432, which is a sclerosing agent that have gained pharmaceutical approval. While sirolimus was approved as a treatment for refractory lymphatic diseases in September 2021, there are no data directly related to the evaluation of this CQ, and further reports are awaited.

#### Lay summary

Some lesions of mediastinal LMs are small and do not require proactive treatment. However, some lesions directly or indirectly compress the trachea or bronchi and cause respiratory disorders by narrowing the airway, in which case prompt treatment is necessary. Typical treatments are surgery and sclerotherapy. Surgery is effective and often yields a favorable outcome but causes complications relatively frequently. Sclerotherapy is effective in many cases and is considered to be relatively free of complications, but is known to be ineffective for microcystic (cavernous) lesions. There are no universal criteria for the selection of treatment, and it is necessary to select treatments according to the size and site of the lesion in each patient.

### CQ32: Which treatment is recommended for sclerotherapy be performed in infancy for a patient with head and neck LM affecting the airway?

Recommendation: If there is a lesion that causes symptoms of airway stenosis or has a high risk of causing symptoms, therapeutic intervention is necessary even in infancy. Both surgery and sclerotherapy are likely to be effective but have the risk of complications, and the selection of treatment for each case is necessary. Also, precautions against exacerbation of symptoms of airway stenosis are necessary in the treatment.

Strength of recommendation: 2 (weak)

Evidence: D (very weak)

#### Process of preparation of recommendation

##### 1．Circumstances that make CQ an important clinical issue

In LM distributed around the airway in the neck, airway stenosis is an important problem.

Although sclerotherapy is effective for macrocystic lesions, there is concern over the appearance or exacerbation of symptoms of airway stenosis, because the treatment causes temporary local swelling of the airway. In surgical resection, cosmesis after surgery is extremely important along with attention to intraoperative nerve injury, and there is concern over lymphorrhea from the residual lesion, re-accumulation of lymph, and recurrence.

In addition, since the upper airway expands and becomes less vulnerable with the growth, it tends to become less prone to symptoms of stenosis. Therefore, the judgment of whether LM around the cervical airway should be proactively treated in infancy is occasionally difficult.

##### 2．Evaluation of evidence

###### <Literature search>

As a result of a systematic literature search, 113 papers in Japanese and 235 papers in European languages were retrieved and subjected to primary screening. As a result, 20 papers in Japanese and 93 papers in European languages were subjected to secondary screening. Among them, 8 papers in Japanese and 54 papers in European languages referred to this CQ. They consisted of 1 systematic review, 1 RCT, 3 prospective studies, 4 retrospective cohort studies, and 2 cross-sectional studies, but many of the rest were case series or case reports.

###### <Evaluation>

Since the systematic review by Adams *et al.* evaluated LM of the entire head and neck region and was not restricted to LM located around the airway, it is not completely relevant as the answer to this CQ^673)^. In this systematic review 13 of the 277 patients died (mortality rate, 4.7%). In addition, all 13 patients died at the age of less than 1 year, 8 were judged to have died due to airway malfunction such as airway obstruction and aspiration due to paralysis of the vocal cord, and at least 1 died due to complications of invasive treatment. Therefore, this systematic review could be data that recommend some therapeutic intervention in infancy.

Through literature screening, LM around the cervical airway was shown to be treated by sclerotherapy, surgery, or pharmacologic treatments.

####### ■Sclerotherapy

Sclerosing agents used as key words for the present published work search varied widely and included OK-432, bleomycin, ethanol, doxycycline, sodium tetradecyl sulfate and fibrin glue, but OK-432, or bleomycin, or their combination were employed in many reports.

There were several reports that evaluated the regression rate by sclerotherapy in patients that developed symptoms of airway stenosis due to lesions located around the airway. While a relatively high regression rate was obtained in patients that underwent OK-432 sclerotherapy^573,674)^, the results of scherotherapy using doxycycline were reported to be unsatisfactory^577)^.

There were sporadic reports of alleviation of symptoms among those on changes in symptoms of airway stenosis or dysphagia after treatment. All of them reported relief from symptoms after sclerotherapy using doxycycline^577,675)^.

As for complications associated with treatments of lesions around the airway, many papers reported temporary complications associated with sclerotherapy such as fever, local swelling and pain, intracystic hemorrhage, and infection, and some referred to complications considered to have been related to treatment of head and neck lesions such as respiratory disorders due to airway stenosis/obstruction and nerve paralysis. There was also a report that caution is needed in OK-432 sclerotherapy near the airway, particularly, in small children^674)^.

Regarding serious complications related to other sclerosing agents, there were reports of permanent paralysis of the vocal cord due to topical ethanol injection^559)^, death due to pulmonary embolism due to OK-432^561)^, death due to pulmonary complications after treatment with bleomycin^676,677)^, and leukocytopenia^678)^.

####### ■Surgical treatments

In the systematic review by Adams *et al.*, surgical resection (plus other treatments) was selected in a majority of cases^673)^. However, surgery was performed after sclerotherapy or pharmacologic treatments in some reports.

The results were satisfactory^536,679)^. Particularly, the outcomes were better in macrocystic LM than in microcystic LM, and the treatment response was better with fewer complications in the former.

The incidence of complications was reportedly 17.5%^679)^ and 13.8%^536)^, and neuropathy, infection, hematoma, lymphorrhea, transient facial nerve paralysis, transient recurrent nerve paralysis, eating difficulties, and 1 case of death were reported as complications.

######## ■Pharmacologic treatments

According to the present review of the literature, sildenafil^680)^, *Eppikajuttou*^681)^, and sirolimus^589,682,683)^ were used.

Relatively favorable outcomes were reported with each drug, but it must be noted that drugs were rarely used alone and were mostly combined with surgical treatment, sclerotherapy, and other drugs.

As complications, eczema, stomatitis, dyslipidemia, nausea, arthralgia, cellulitis, pneumonia, and bleeding in the mass were reported in association with sirolimus, and diarrhea, hemorrhage, and infection were reported in association with sildenafil.

######## ■Comparison between sclerotherapy and surgical treatment

In a systematic review by Adams *et al.*, both nerve damage due to sclerotherapy for head and neck LM and post-therapeutic infection were reported in 1 (0.8%) out of 123 patients. On the other hand, because nerve damage and infection after surgery were observed in 12 (10.2%) and 7 (5.9%) out of 118 patients, respectively, the complication rate would be lower by sclerotherapy than by surgery^673)^.

In a retrospective cohort study of 174 cases of head and neck LM^551)^, the outcomes were compared between those treated surgically and those who underwent sclerotherapy as the initial treatment. It was concluded that there was no significant difference in the necessity of additional treatment after the initial treatment between the 2 groups.

###### <Conclusion>

To summarize the above, there have been some reports on the risk of respiratory distress due to LM around the airway in infants, and therapeutic intervention is considered to be necessary even in infants when they have a high risk or have already developed symptoms. The treatments are primarily sclerotherapy, surgery, and pharmacologic treatments, but management with pharmacologic treatments alone is difficult, and pharmacologic treatments are recommended to be combined with sclerotherapy or surgery. There is no literature with a high evidence level that compared the results between sclerotherapy and surgery. However, in the systematic review by Adams *et al.*, the complication rate is judged to be lower by sclerotherapy than by surgery, initiation of intervention by less invasive sclerotherapy is recommended^673)^. However, sclerotherapy for lesions around the airway is associated with the risk of exacerbation of airway stenosis due to reactive swelling of the lesion. Particularly, since the risk of complication of respiratory disorders is high in children aged less than 2 years, sclerotherapy for lesions around the airway must be initiated with sufficient preparations for securing of the airway and respiratory management in infants.

##### 3．Evaluation of balance of benefits and risks

Alleviation of symptoms and regression of the lesion were observed by both sclerotherapy (particularly OK-432 sclerotherapy) and surgery. Whether sclerotherapy or surgery is more effective is difficult to judge, it is possible to consider that the complication rate is lower by sclerotherapy than by surgery. For the management of life-threatening symptoms, both treatments may have more benefits than risks.

##### 4．Patients’ values/wishes

Alleviation of symptoms and regression of the lesion, which makes resection of the lesion possible, by sclerotherapy conform to the patients’ wishes. If complications of sclerotherapy do not threaten the patients’ lives or markedly impair their QOL, and if they are milder than those of surgical treatment, sclerotherapy for LM around the airway in infants is considered to conform to the patients’ values/wishes.

##### 5．Cost assessment, assessment of external validity of intervention

Presently, only OK-432 is approved by National Health Insurance in Japan as a sclerosing agent for LM. Surgery is approved as a treatment for LM. While sirolimus was approved as a treatment for refractory lymphatic disorders in September 2021, no data that directly affect the evaluation of this CQ are as yet available, and their reports in the future are awaited. The appropriateness of each intervention needs to be evaluated based on the condition of each patient.

#### Lay summary

LM distributed around the cervical airway may cause airway stenosis. If a patient already has or is at a high risk of developing symptoms of respiratory distress, prompt treatment is necessary even in the neonatal period or infancy. Both surgery and sclerotherapy are expected to be effective, but both have a risk of complications. Surgery and sclerotherapy are effective as primary treatments, and their relative superiority is unclear. While both are known to have a risk of complications, their incidence has been reported to be lower by sclerotherapy, and less invasive sclerotherapy is more often selected first. Sclerotherapy must also be performed with sufficient preparations for respiratory management, particularly in infants, because of the possibility of exacerbation of airway stenosis due to swelling. There are also pharmacologic treatments using drugs, but they are recommended to be performed in combination with sclerotherapy or surgery.

### CQ33: What treatments are effective for LM of the tongue?

Recommendation: Surgical resection is effective for reducing the size of the lesion and alleviating symptoms and functional impairment. It is often performed with other treatments including sclerotherapy. However, total resection is often difficult depending on the distribution of the lesion. Careful evaluation of the surgical indications is required in consideration also of possible complications and recurrence. Cauterization and laser may also be considered for localized lesions such as lymphatic vesicles.

Strength of recommendation: 2 (weak)

Evidence: D (very weak)

#### Process of preparation of recommendation

##### 1．Circumstances that make CQ an important clinical issue

While the tongue is one of the frequent sites of LM, the lesion is often distributed widely over the neck rather than localized in the tongue. LM of the tongue not only cause cosmetic problems, such as protrusion from the mouth and bleeding, but also readily occupy the oropharyngeal cavity and cause functional problems such as disorder of mouth closing, difficulty in speaking, respiratory disturbances and impairment of oral food intake. These conditions are treated at departments including plastic surgery, oral surgery, otolaryngology and pediatric surgery. LM of the tongue are treated by surgical resection, sclerotherapy, cauterization, and laser therapy. In addition to general information, comprehensive evaluation of the condition of individual cases including the distribution of the lesion in the tongue, involvement of other lesions, cyst components, and vascular distribution such as the risk of complications and recurrence in each treatment, is necessary.

##### 2．Evaluation of evidence

###### <Literature search>

As a result of systematic literature search, 38 papers in Japanese and 124 papers in European languages were subjected to primary screening. Of these papers, 5 in Japanese and 29 in European languages were subjected to secondary screening. Since they were all case series or case reports, the results and discussions of all reports were integrated.

###### <Evaluation>

The effectiveness of surgical resection for LM of the tongue was evaluated from the viewpoints of reduction rate, symptoms, functions and cosmetics as well as complications and recurrence.

###### I．Treatment responses

####### A．Reduction rate

Tongue lesions were treated by a wide variety of methods including surgical resection, sclerotherapy, laser (CO_2_, Nd:YAG), and cauterization (radiofrequency, bipolar) alone or in combinations. In many reports, combination therapies in which other treatments mentioned above were added to surgery as the primary constituent.

Catalfamo *et al*.^684)^ performed surgical resection of localized masses with normal structures within areas of 1 cm horizontally from the masses and reported that the lesion size could be reduced in 8 (88.9%) of the 9 cases.

Concerning large lesions involving the areas around the airway, Azizkhan *et al.* reported 21 cases of surgical resection, but several operations were often necessary to reduce lesion size^685)^. A total of 2 cases have been reported, and the lesion size was reduced in both^686,687)^. Although differences were observed in re-enlargement after surgery, they are discussed in detail in “(II) Complications”.

In a case report, sclerotherapy was performed 15 times, but the lesion size could not be reduced, and surgical resection was performed, eventually resulting in a favorable outcome without recurrence^688)^.

According to a report of 89 cases of head and neck LM by Lei *et al.*, the outcome was excellent in 73 (82%) and good in 16 (18%) although it was not a report of cases of tongue lesions alone^542)^. They included 43 cases of tongue lesions.

In addition, a few papers that suggested the effectiveness of combinations of surgical resection with sclerotherapy and laser therapy were observed^685,689-691)^. Wiegand *et al.* classified the disease into 4 stages based on the area of involvement and reported that the stage can be a prognostic factor^691)^. Surgery was effective, and complications were rare, when the lesion was localized in the superficial layer and part of the muscle layer. Surgical resection can also be effective, but complete resection is difficult, when the lesion extends over the entire muscle layer or to the tongue base and neck. Therefore, partial resection is often repeated and combined with laser therapy and sclerotherapy, but the recurrence is observed very frequently, and the results did not contradict the reports^542)^ mentioned later in the section of the recurrence rate.

As treatments performed alone, reports of sclerotherapy using bleomycin are sporadically observed. There are reports that OK-432 is effective for macrocystic LM but is poorly effective for microcystic LM^680,692)^, and it was performed concomitantly with surgery in many cases. However, Bonet *et al.* reported that 2 of the 8 cases of macrocystic LM of the tongue obtained remission without treatment^693)^, and there was also a report that suggested a relationship between the state of oral hygiene and regression of the lesion^694)^.

The systematically searched literature did not include reports of oral sirolimus treatment, which was approved as a treatment for refractory lymphatic disorders in September 2021.

####### B．Symptoms

A wide variety of symptoms have been reported depending on the site of the mass, and they include tongue discomfort, bleeding, pain and difficulty in oral feeding. Improvements in dysarthria by surgical resection^685)^ and its combination with sclerotherapy^695)^ have been reported. Also, sporadic reports of alleviation of bleeding from the tongue surface, pain and eating difficulty by cauterization^696-698)^ and effectiveness of CO_2_ laser^699)^ and Nd:YAG laser^700,701)^ for the treatment of lymphatic vesicles and bleeding are observed.

####### C．Functions

In most patients who exhibited functional impairment, the lesions were so extended that they were no longer indications for one-time surgical resection. Large masses located at sites such as the tongue base cause respiratory disturbances, swallowing disorders and difficulty in speech. In the report by Azizkhan *et al.*, oral intake of normally cooked food became possible in 14, and normal vocalization became possible in 8, of the 21 patients with tongue base lesions after some surgical treatment^685)^.

####### D．Cosmetics

Objective evaluation of cosmetic effects is also difficult. Azizkhan *et al.* reported that, of the 20 long-term survivors who underwent some surgical treatment, excluding1 with severe deformity who died, deformity of structures around the tongue, such as the mandible and maxilla, was mild in 6, moderate in 5 and severe in 9^685)^. There have been a few reports that cosmetic improvements were also observed in patients who showed a reduction of the tongue size by surgical resection, but objective evaluation is insufficient.

###### II．Complications

Although the properties of the lesions are unclear in some papers, facial nerve paralysis, vagus nerve paralysis, infection, hematoma, seroma, salivary leakage, ruptured suture and skin flap necrosis have been reported as complications associated with surgical resection in the facial region^542,685)^. There have also been reports of temporary complications such as pain, hemorrhage, and ulceration of the mucosal surface associated with sclerotherapy^702)^. If the lesion extends to the areas around the airway, there have been reports of airway stenosis due to swelling after sclerotherapy and death due to airway obstruction, and caution is needed^685,691)^.

###### III．Recurrence

There have been a few reports that recurrence requiring clinical treatment was not observed. On the other hand, Lei *et al.* reported that recurrence was observed in 21 (23.6%) of 89 patients and was more frequent in those aged less than 1 year, those with lesions in the oral cavity/face, those with lesions at 3 or more sites and those with microcystic lesions^542)^. Of the 2 patients treated by surgical resection alone, one who underwent resection of the middle part of the tongue showed no re-enlargement for 1 year or longer after surgery^686)^, but surgery was repeated 3 times in the one who underwent marginal resection^687)^. Although details are unclear, mild re-enlargement was reported to have occurred in 3 of the 4 patients who underwent debulking surgery, but it was manageable by follow-up without treatment^703)^.

Concerning sclerotherapy for microcystic LM, there have been reports of rapid enlargement of the lesion and exacerbation of obstruction of the upper airway in 8 of the 15 patients administered bleomycin^704)^ and moderate recurrence in 2 of the 7 patients after cauterization^698)^.

Also, Boardman *et al.* studied 97 patients with LM of the head and neck and reported that, by cauterization or laser therapy, treatment was needed several times in 6 of the 7 patients with tongue lesions and that patients with tongue lesions accounted for a markedly higher percentage of those who required treatment 3 or more times compared with patients with lesions in other parts of the head and neck^670)^.

####### ■Limitations

In some papers, surgical resection was combined with other treatments^685,688-691,696)^, lesions in other areas such as the neck were included^542)^ and the lesion types were unknown. The lack of standardization of subjects and uniformity of the definition or time of recurrence must be considered in the evaluation of the effectiveness of each treatment.

###### <Conclusion>

Many papers suggest that surgical resection is effective for reducing the size of tongue LM. However, in patients with large lesions, lesions extending to structures other than the tongue, and microcystic lesions, several resections or combination of resection with other treatments such as sclerotherapy and laser therapy were necessary, and the recurrence rate tended to be higher. While a few papers referred to symptoms, functional outcome and cosmetic improvements, none showed a high level of evidence, and the evidence was insufficient for general discussion of the effective treatment.

Therefore, the recommendation statement concerning effective treatments for lingual LM was made, “Surgical resection may be effective for reducing the lesion size and alleviation of symptoms and functional impairment, it is often performed in combination with other treatments such as sclerotherapy. However, total resection is often difficult depending on the distribution of the lesion, and careful decision-making is required in consideration of the possibility of complications and recurrence. Cauterization and laser therapy may also be considered for localized lesions such as lymphatic vesicles.”

##### 3．Evaluation of balance of benefits and risks

Some reduction of the lesion size, associated alleviation of symptoms, and improvements in QOL have been reported to be achieved primarily by surgical resection but also by sclerotherapy, laser, or their combinations. The benefit is often reduction of lesion size. While the evidence is insufficient, these treatments are considered beneficial. On the other hand, risks including invasiveness of treatment, occurrence of complications, and recurrence are not small. Although the benefits are generally considered to surpass the risks, the selection of treatment need to be made carefully.

##### 4．Patients’ values/wishes

All treatments aimed to improve cosmetic and functional problems of tongue lesions conform to the patients’ and their families’ values. The selection of treatments based on thorough knowledge about the possibility of complications and recurrence is considered to meet the patients’ values, but the values of patients may vary depending on the severity of the lesion and expected degree of improvement.

##### 5．Cost assessment, assessment of external validity of intervention

Surgical resection and sclerotherapy are generally considered effective for LM, they are covered by National Health Insurance in Japan, and their cost is reasonable. Regarding laser and cauterization, it is necessary to sufficiently evaluate the validity of intervention in each case, because the extent of the lesion and symptoms vary widely, and the possibility of complications and recurrence differ, among individual cases.

#### Lay summary

LMs of the tongue are reported to be treated primarily by surgery, frequently by sclerotherapy or their combinations. Although these treatments are considered to be effective for reducing the size of the lesion and alleviating symptoms, complete removal/eradication of the lesion is often difficult to achieve depending on the size of the lesion and the degree of its extension around the tongue, and complications and recurrence are not rare. It is necessary to carefully evaluate the indications based on the extent of the lesion and symptoms. Treatments such as cauterization and laser therapy may be considered for localized lesions such as superficial lymphatic vesicles.

### CQ34: Is aggressive surgical intervention effective for chylous pleural effusion in the neonatal period?

Recommendation: Although treatments such as pleurodesis, thoracic duct ligation, and pleuroperitoneal shunt have been reported, the evidence concerning their effectiveness is not sufficient, and they should be considered only when all conservative treatments have proved to be ineffective.

Strength of recommendation: 2 (weak)

Evidence: D (very weak)

#### Process of preparation of recommendation

##### 1．Circumstances that make CQ an important clinical issue

Chylous pleural effusion during the neonatal period is often refractory and can be fatal. Thoracic drainage is performed for respiratory failure due to accumulation of pleural effusion simultaneously with conservative treatments including nutritional therapy, steroids, and octreotide therapy until resolution of chylous thoracic effusion. In refractory cases that do not respond to these conservative therapies, surgical intervention, such as ligation of the thoracic duct and pleurodesis, may be performed. However, no sufficient consensus has been obtained concerning their effects. Currently available information about issues such as at what point surgical intervention should be made and for what conditions aggressive surgical intervention is effective was evaluated comprehensively.

##### 2．Evaluation of evidence

###### <Literature search>

As a result of a systematic literature search, 127 papers in Japanese and 351 papers in European languages were subjected to primary screening. Of these papers, 8 in Japanese and 27 in European languages were subjected to secondary screening. They included no paper with a high evidence level such as a systematic review or RCT aimed to evaluate surgical treatments, and all were case series or case reports. Therefore, the results and discussion in each of the case series judged to be useful for the preparation of the recommendation statement of this CQ were integrated, although they are deficient as evidence.

###### <Evaluation>

The published work concerning the effectiveness of surgical treatment for chylous pleural effusion in the neonatal period was reviewed from the viewpoints of responses and complications.

###### I．Treatment responses

Surgical treatments for chylous thoracic effusion in the neonatal period are performed in refractory cases that do not respond to conservative treatments such as total parenteral nutrition, nutritional therapy using medium-chain triglyceride milk, and octreotide administration in addition to pleural drainage.

The methods for surgical intervention found by the present published work review included ligation of the thoracic duct and pleuroperitoneal shunting as well as pleurodesis with OK-432 administration, intrathoracic infusion of fibrin and povidone-iodine administration. Some patients diagnosed *in utero* underwent pleuro-amniotic shunting. Also, as less invasive treatments, thoracoscopic ligation of the thoracic duct, intrathoracic fibrin application, and pleural clipping have been reported^705)^ in addition to thoracic duct ligation by thoracotomy. Recently, lymphangiography by inguinal lymph node puncture and lymphatic embolization have been performed even during the neonatal period with some reports of success, but many of the successful cases were those of acquired chylous thoracic effusion, and the treatments are considered to be scarcely effective for congenital lymphatic anomalies^639,706)^.

Treatments performed before surgical treatments or their durations varied. Also, there were cases of chylous thoracic effusion that appeared secondarily after surgical treatments and cases of congenital chylothorax, and the diversity of the patient background must be taken into consideration in efficacy evaluation.

Among the surgically treated cases, those in whom chylous plural effusion disappeared, respiratory symptoms were alleviated and weaning from the respirator became possible have been reported^707,708)^. In addition, the absence of recurrence or reactivation is considered to be a point^707-710)^. Cleveland *et al.* considered conservative treatments, such as total parenteral nutrition, octreotide and diuretic administration, to be the best. However among them with poor responders, the mortality was 80% in 5 who continued to be managed by conservative treatments but 0% in 4 who underwent additional surgery, reporting that surgical treatment contributed to the reduction of the mortality^711)^. Church *et al.* reported that a drain volume during the first 24 hours after surgery of >20 cm^3^/kg/day and a lymphocyte count of 1000 cells/μL as prognostic factors for the future requirement of surgical treatment for chylous thoracic effusion cases in which surgical intervention was necessary^712)^. In the guidelines for the treatment of chylous thoracic effusion by Büttiker *et al.*, conservative treatment should be continuing for approximately 3 weeks but should be abandoned thereafter because of the risk of nutritional disturbance, and increased susceptibility to infection and liver disorders^713)^. However, Kaji *et al.* reported that it is difficult to set a clear period of conservative therapy, because the effectiveness and success rate of surgical treatment are unclear^714)^.

In cases of congenital chylothorax diagnosed *in utero*, improvements in the Apgar score, shortening of the period of respiratory management, and reductions of sepsis and thrombotic complications by fetal treatments such as pleural puncture and pleuro-amniotic shunting have been reported^715)^.

###### II．Complications

As complications due to sclerosing agents, fever and increased inflammatory reaction due to the administration of OK-432 as well as pulmonary abscess and temporary flaccidity and protrusion of the upper abdominal region considered to have been due to intercostal nerve damage have been reported. While chyle leakage in the abdominal cavity was noted in a patient who underwent pleuroperitoneal shunting, there were no reports of fatal complications. Among the patients who underwent lymphatic embolization, the embolic agent embolized the pulmonary artery in 1^706)^.

####### ■Limitations

Surgical treatment was performed in most reported cases when responses to conservative therapy were not obtained. Therefore, it must be assumed that the results of evaluation of this CQ are based on data concerning the effectiveness of surgery performed with conservative therapy.

###### <Conclusion>

The published work was reviewed concerning the effectiveness of aggressive surgical intervention for neonatal chylous thoracic effusion from the viewpoints of responses and complications, but no objective study with a high level of evidence was found. In most cases, surgical treatments including sclerotherapy (pleurodesis) were performed when responses to conservative treatments were poor. Therefore, comparison of surgical treatments with other treatments have not been made. The evaluation of the period of conservative treatment before surgery remains insufficient, but there was a paper that proposed surgical intervention after attempting conservative treatments for 3 weeks.

Thus, surgical intervention for neonatal chylous pleural effusion is characterized at present as an approach that should be evaluated when the condition is not improved by other treatments.

##### 3．Evaluation of balance of benefits and risks

The degree of benefits of surgical treatments such as pleurodesis, thoracic duct ligation, and pleuroperitoneal shunting for neonatal chylous thoracic effusion is difficult to evaluate and is not considered to be uniform. On the other hand, elements of risks are relatively large if they occur, and sufficient evaluation is needed in implementing surgical intervention.

##### 4．Patients’ values/wishes

There is no treatment that is consistently effective for neonatal chylous thoracic effusion, and its treatment is often prolonged. It is inevitable to perform conservative/pharmacologic treatments first and, if they are ineffective, to opt for surgical treatments while they are invasive and have the risk of complications. This approach is considered to conform to the patients’ values/wishes.

##### 5．Cost assessment, assessment of external validity of intervention

It is inevitable to select surgical treatments if other treatments are ineffective although there is no sound evidence for the choice. It is considered reasonable to try treatments in the order of increasing invasiveness and to choose surgical treatments if necessary.

#### Lay summary

There is no treatment that is consistently effective for chylous thoracic effusion in the neonatal period, and the treatment is often prolonged. If necessary to improve respiration, pleural drainage is performed first to drain thoracic effusion. Then, to stop accumulation of pleural effusion, conservative/drug treatments that cause less burden on the body, i.e., nutritional therapies such as total parenteral nutrition and medium-chain triglyceride and octreotide administration are performed. If they are not sufficiently effective, surgical treatments such as pleurodesis, thoracic duct ligation, and pleuroperitoneal shunting are considered, but they cause a large burden on the body and involve the risk of complications. Since the effectiveness of surgery for chylous thoracic effusion is uncertain, surgery should be evaluated only when improvements by other treatments are poor.

### CQ35: What treatments are effective for generalized lymphatic anomaly (GLA) and Gorham-Stout disease (GSD) that cause refractory chylous pleural effusion, pericardial effusion, and respiratory disturbance?

Recommendation: Treatments including nutritional therapy, drug therapy, surgery, sclerotherapy, and radiation therapy are performed in various combinations as necessary. Among them, sirolimus is a treatment that should be considered preferentially.

Strength of recommendation: 2 (weak)

Evidence: D (very weak)

#### Process of preparation of recommendation

##### 1．Circumstances that make CQ an important clinical issue

GLA/GSD, which is a refractory disease that causes a variety of symptoms in the entire body is also difficult to diagnose, is particularly lethal when lesions are present in the thoracic region.

Among the thoracic symptoms, chylous pleural effusion/pericardial effusion are often refractory and occasionally fatal. While information about the disease is extremely limited because of its rareness, case reports are being globally accumulated as chronic cases are managed on an outpatient basis, and as severe cases are treated intensively. In addition, research on drugs including sirolimus has recently been actively underway.

Presently, no radical treatment for these refractory diseases is known, but the CQ was formulated to compile the knowledge about what treatments are effective as it is a problem of clinical importance.

##### 2．Evaluation of evidence

###### <Literature search>

As a result of a systematic literature search, 1,104 papers (311 in Japanese, 793 in European languages) were subjected to primary screening. As a result of primary screening, 72 papers (17 in Japanese, 55 in European languages) were subjected to secondary screening, through which 51 papers (6 in Japanese, 45 in European languages) were adopted. Also, as a result of hand search, 1 observational study, 1 cohort study, and 2 case series studies were added to the references from 210 papers in European languages.

###### <Evaluation>

The effectiveness of various treatments for refractory GLA and GSD was evaluated based on the prognosis and the presence or absence of improvement in imaging findings, improvement in symptoms, improvement in airway obstruction, enlargement of the lesion, regression, treatment-related complications, recurrence and reactivation. The cause of chylous pleural and pericardial effusion is lymphorrhea from lymphatic vessel tissue lesions that have primarily invaded the mediastinum and pleura, and lymphorrhea from osteolytic lesions of the ribs and vertebrae was also observed. Respiratory disturbances were caused by pleural effusion, chylous pleural effusion, pericardial effusion, and direct invasion of the mediastinum and lungs.

In the reviewed references, the following treatments were performed for GLA and GSD causing refractory chylous thoracic effusion, epicardial effusion, and respiratory distress.

・Surgical treatments: Tumor resection, thoracentesis, pleural decortication, thoracic duct ligation, pericardiocentesis, pericardial fenestration, lung transplantation, lymphaticovenous anastomosis

・Pleurodesis (sclerotherapy)(OK-432, minocycline, talc, mistletoe extracts, monoethanolamine oleate)

・Radiation therapy

・Nutritional therapy: Fasting, high-calorie infusion, medium-chain triglyceride, low-fat diet

・Drug therapies: Sirolimus, interferon-α, steroids, octreotide, molecularly targeted drugs (e.g., imatinib, sunitinib), propranolol, anticancer agents (e.g., vincristine), bisphosphonate, low-molecular-weight heparin, diuretics, bronchodilators, immunoglobulin preparations, albumin preparations

・Others: Lymphatic embolization, lymphaticovenous shunting, pleurovenous shunting, pleuroperitoneal shunting

In most reports, these treatments were performed in combinations rather than alone. Each treatment is evaluated below.

####### ■Surgical treatments

As surgical treatments for chylous pleural effusion, procedures, such as thoracentesis, thoracic drainage, ligation of the thoracic duct, pleural decortication, and pleurodesis have been performed, and local lesions were surgically resected. In most cases, thoracentesis and thoracic drainage were performed, but chylous thoracic effusion was not resolved by these procedures alone. As for complications, there was a patient who developed hypovolemic shock and required blood transfusion and catecholamine administration^716)^.

In the literature concerning thoracic duct ligation^717-733)^, chylous thoracic effusion was markedly relieved in 4 cases^720,722,728,730)^, but all 3 were treated by combinations of thoracic duct ligation with other surgical procedures, radiation therapy, or bisphosphonate. Also, while no improvement was observed in chylous thoracic effusion, there was1 case that showed improvement in respiratory disturbance^726)^. As complications of ligation of the thoracic duct, splenomegaly and lymphorrhea^725)^ and left-sided pleural effusion^717,725)^ have been reported.

In the literature concerning pleural decortication^716,721,723-725,728,734-737)^, marked improvement in chylous thoracic effusion was observed in 3^716,725,728)^, but they were all treated by combinations of pleural decortication with surgery or sclerotherapy, and there was no mention about complications.

In the literature concerning surgical resection of local lesions including splenectomy^717,720,725,728,735,737-741)^, marked improvement of chylous thoracic effusion was observed in 4^720,725,728,737)^, but 3 were treated by combinations with other surgical procedures^720,725,728)^. Massive hemorrhage was reported as a complication^738)^.

Among other treatments, pleuroperitoneal shunting^723)^ and lung transplantation^742)^ were performed, and alleviation of respiratory disturbance was noted in the patient who underwent lung transplantation. There was also a report on pulmonary artery embolization and pneumonectomy for poorly controlled hemothorax and hemoptysis, and their combination with pharmacologic treatment was effective^735)^.

There was a report that lymphaticovenous anastomosis was performed in 14 cases of central conducting lymphatic anomaly (CCLA) accompanied by continuous thoracic effusion, resulting in complete remission in 5 and partial remission in 2^743)^. Information about whether the procedure is equally effective for chylous thoracic effusion associated with GLA and GSD, which are the topic of this CQ is awaited.

As a surgical treatment for pericardial effusion, pericardiocentesis was performed^737,744-747)^, and pericardial fenestration was performed when pericardial effusion could not be controlled by pericardiocentesis^737,746,747)^. According to the paper by Du *et al*.^747)^, not only pericardial effusion but simultaneously also chylous thoracic effusion was resolved. There was no mention of complications.

Thus, while various surgical procedures have been performed, the number of reported cases is small, and the evidence level of the literature concerning their effectiveness is considered low.

####### ■Sclerotherapy

As sclerotherapy for chylous thoracic effusion, pleurodesis was performed using a wide variety of drugs^716-719,724,728,731-733,735,739,746,748-754)^. OK-432^716,718,719,731,733,740,752)^, talc^728,735,748)^, mistletoe extracts^732)^, minocycline^749)^, and monoethanolamine oleate^752)^ were used as sclerosing agents. Marked improvement in chylous thoracic effusion was observed in 5 patients^716,718,728,731,739)^, and 3 of them were treated by combinations with surgical procedures including pleural decortication^716,718,728)^. In 1 case that showed improvement of respiratory disturbance but not of chylous thoracic effusion, sclerotherapy was performed in combination with local radiation therapy (30.6 Gy)^750)^. There was no mention of complications of sclerotherapy. At any rate, all reports were about a single case, so the number of reported cases is small, and the evidence level of the literature concerning the therapeutic effect is considered low.

####### ■Radiation therapy

There were reports on local (e.g., lesion area, thoracic duct region) and thoracic radiotherapy for chylous pleural effusion and local lesions^719,720,722-724,729,733,738-740,745,746,748,750,755-757)^. The radiation dose varied considerably, being 9–40 Gy. Of the 3 cases that showed marked improvement in chylous thoracic effusion^720,722,744)^, the radiation dose was 40 Gy in 2^722,744)^. Also, it was 30 Gy in the 2 cases that showed relief from respiratory disturbance but no marked improvement in chylous thoracic effusion^736,740)^. Three of the 5 cases that showed responses were treated by combinations of radiation with other treatment^720,722,740)^. Radiation pneumonitis has been reported as a complication^736)^. In 1 case, irradiation at 40 Gy was planned, but the treatment was discontinued at 25 Gy because of progression of respiratory insufficiency^757)^. At any rate, all reports were about a single case, so the number of reported cases is small, and the evidence level of the literature concerning the therapeutic effect was considered low.

####### ■Nutritional therapy

Fasting, high-calorie infusion^716,718-720,723,725,728,737,748,758)^, or medium-chain triglyceride^716,718,725,733,737,747,748,754,758,759)^ were used. They were often used in combination^716,718,725,737,745,748,758)^. There was a report of only 1 case that showed a decrease in chylous thoracic effusion by a combination of high-calorie infusion and medium-chain triglyceride^745)^. Low-fat/fat-restricted diet was also attempted^736,747,760-762)^. At any rate, the reports were about a single case, and the number of reported cases was small, so the evidence level of the literature concerning the therapeutic effect is considered low.

####### ■Drug therapies

For drug therapy against chylous pleural effusion, drugs including sirolimus, interferon-α, steroids, propranolol, molecularly targeted drugs, anticancer agents (e.g., vincristine), bisphosphonate, octreotide, and low-molecular-weight heparin were used.

Regarding sirolimus, 1 observational study and 2 case series have been reported. Ozeki *et al.* administered sirolimus (target blood concentration: 5–15 ng/mL) to a total of 20 cases of lymphatic diseases including 3 cases of kaposiform lymphangiomatosis (KLA), 3 cases of GLA, 6 cases of GSD, and 3 cases of CCLA and reported significant improvements in the symptoms score and QOL score^682)^. Adverse events (gastritis, infection, hyperlipidemia) were noted in 16 of the 20 cases, and 3 of them had grade 3 infection (upper airway infection, cellulitis, pneumonia). None of them required discontinuation of oral sirolimus administration. Also, Triana *et al.* and Ricci *et al.* administered sirolimus to 41 cases (including 6 cases of GLA, 7 cases of GSD, and 1 case of KLA) and 18 cases (all of GSD), respectively, and reported partial responses in 33/41 cases and 15/18 cases, respectively^485,650)^. Regarding adverse events, Triana *et al.* reported hyperlipidemia and high liver enzyme levels (treated by statin administration) in 1 case and lymphocytopenia and infection in another^485)^. Ricci *et al.* reported a high incidence of bone marrow suppression and the occurrence also of stomatitis/gastrointestinal symptoms and hyperlipidemia^650)^.

Sirolimus was also used many case reports^736,741,744,752,760-765)^. Improvements in respiratory disturbance were reported in 4 cases^485,736,744,764)^, and there were also reports of confirmation of a decrease in thoracic effusion^736)^ and regression of the lesion in the chest wall^752)^. Hypertension^744)^, hyperlipidemia^736,752)^, stomatitis^736)^, and diarrhea^752)^ have been reported as complications of drug therapy using sirolimus.

There are also sporadic papers on the use of interferon-α^716-718,720,721,723,736,737,745,752,757,758,761,763)^, reporting improvement of chylous thoracic effusion in 7^716,717,720,723,758,762,763)^ but subsequent reactivation in some^762)^. Among them, interferon-α was used in combination with propranolol in 1^716)^, with low-molecular-weight heparin or local radiotherapy (15 Gy) in 1^720)^, and with bisphosphonate in 2^682,761)^. As complications of drug therapy using interferon-α, fever^758)^, nausea and headache^757,758)^, thrombocytopenia^717)^, liver disorder^717,761)^, and hemolytic anemia^761)^ were reported.

There were also many papers on the use of steroids^485,716,719,723,736,745,752,761,763)^ or octreotide^716-718,720,723,725,733,736,748,760-763)^, but they were invariably used in combination with other therapies. There was a report that chylous thoracic effusion was improved in a case treated with a combination of octreotide, propranolol, and radiation^748)^.

There were also sporadic reports of the use of molecularly targeted drugs, which included sunitinib^754)^, sorafenib^735)^, and imatinib^735)^, and relief from chest pain, respiratory insufficiency, and hemoptysis^735)^ by imatinib administration was reported. By sunitinib administration, respiratory insufficiency was improved but was reactivated later^754)^.

Sirolimus was the only drug with considerable number of case series or prospective studies and is considered at present to be the treatment with the largest volume of information. However, the number of cases was small in these observational studies and case series, and their evidence level was not considered high. Concerning other drugs, there were only reports of a single case, and their evidence level concerning the therapeutic effect was considered low.

####### ■Others

There was a report of lymphatic embolization performed in 3 neonates with chylous thoracic effusion^706)^. Although the treatment was effective for postoperative chylothorax, no improvement was observed in 1 case of congenital chylous thoracic effusion. At any rate, the reports were all about a single case, and the number of reported cases was small, so the evidence level of the literature about the therapeutic effect was considered low.

Ozeki, *et al*. reviewed a total of 85 cases by a questionnaire survey about GLA/GSD in Japan. While there was no detailed report about the effectiveness of the treatment, the data are summarized here, because they are valuable data in Japan. The cases consisted of 9 of KLA, 41 of GSD, and 35 of GLA, and they had bone lesions, thoracic lesions (pleural effusion, mediastinal mass, pericardial effusion), abdominal lesions (spleen, ascites), and skin lesions. As treatments, drug therapy, surgery, irradiation, and nutritional therapy were performed. Pleurocentesis, pleurodesis, and thoracic duct ligation were performed as surgical intervention for pleural effusion. Drug therapies included corticosteroid, propranolol, interferon-α, octreotide, and sirolimus. Radiotherapy was performed in 16 cases (consisting of 6 with bone lesions associated with GSD and 10 cases with thoracic lesions). The mortality rate during a mean observation period of 7 years was 20% (17/85), and all deaths were caused by thoracic lesions. None of the patients without thoracic lesions died. Also, of the 69 children, 50 had thoracic lesions, with death of 13. In GLA/GSD, the importance of the control of thoracic lesions including chylous thoracic effusion was suggested^766)^.

###### <Conclusion>

GLA or GSD causing refractory chylous thoracic effusion, pericardial effusion, and respiratory disturbance was treated by surgery (e.g., pleural decortication, thoracic duct ligation, lymphaticovenous anastomosis), pleurodesis (e.g., OK-432), drug therapy (e.g., sirolimus, interferon-α, steroids), radiotherapy, nutritional therapy, or others (e.g., lymphatic embolization).

Of these treatments, there were observational studies and large case series about sirolimus alone. Although a high response rate of about 80% was reported by sirolimus administration, the total number of cases was small (18–41), and the evidence level of these reports was not high. Adverse reactions, which included hyperlipidemia, infection, and cytopenia, were generally mild. Regarding other treatments, those used in individual case reports were collected. Various treatments were performed in combination in many case reports, and the evidence level concerning the therapeutic effect of each treatment is considered low.

###### <Summary>

Analysis was made to evaluate the CQ “What treatments are effective for GLA and GSD that causes refractory chylous thoracic effusion, pericardial effusion, and respiratory disturbance?”, but most of the references were case series or case reports as in the preparation of the previous edition, The Japanese clinical practice guidelines for vascular anomalies 2017. Various treatments, such as surgery, sclerotherapy, radiotherapy, nutritional therapy and drug therapy, have been performed, but there was no study with a sufficient number of cases and a high level of evidence because of the rareness of the disease and diversity of symptoms. Among them, many observational studies and clinical studies have been conducted about sirolimus therapy, and a high response rate of about 80% was reported, but there was no report that complete remission was attained. In addition, the total number of cases was small (18–41), and the evidence level was not high. Adverse reactions, which included hyperlipidemia, infection, and cytopenia, were generally mild. Concerning other treatments, those performed in individual cases were collected. Various treatments were performed in combination in many case reports, and the evidence level concerning the therapeutic effect of each treatment was considered low.

Since this disease is extremely difficult to treat and lethal, even nonradical treatments that reduce the amount of thoracic effusion or alleviate clinical symptoms are beneficial to patients. While it is difficult to cure the disease by a single therapy, selecting effective treatments and performing them in combination is beneficial as it improves the response rate. The selection of less invasive treatments with fewer adverse responses and complications is beneficial to patients. Adverse reactions to sirolimus are generally reported to be mild, and, although the evidence level is not high, sirolimus shows a high response rate, is the only drug approved as a treatment for this disease, and is considered promising as first-line treatment. However, there remain questions to be clarified such as severe adverse reactions and long-term risk of complications, and further accumulation of evidence is needed.

##### 3．Evaluation of balance of benefits and risks

This disease is extremely refractory and lethal. If not radical, treatments that reduce the amount of thoracic effusion or alleviate clinical symptoms are beneficial to patients. Its treatments include nutritional therapy, drug therapy, and surgery, and they are bound to be performed in combinations in refractory cases. Selecting less invasive treatments with fewer risks such as adverse reactions or complications is beneficial to patients. Careful evaluation is needed, particularly, in choosing surgical treatments because of the possible risk of complications. There have been many reports that sirolimus therapy has a high response rate generally with mild adverse reactions, and its benefits are often considered to surpass the risks. However, there remain questions to be clarified such as severe adverse reactions and long-term risk of complications, and further accumulation of evidence is needed.

##### 4．Patients’ values/wishes

GLA/GSD is a refractory disease, and the selection of highly effective treatments is considered to conform to the patients’ values/wishes. While recovery from a lethal condition and improvement in survival rate can be obtained by choosing more effective treatments, selecting highly invasive treatments or treatments with unknown risk of long-term adverse reactions may not conform to the patients’ values/wishes.

##### 5．Cost assessment, assessment of external validity of intervention

Surgical treatments, sclerotherapy, and steroids are covered by National Health Insurance in Japan and do not cause a heavy burden also to medical economy. Among drugs, sirolimus, which has been confirmed to be effective, is approved as a treatment for refractory lymphatic diseases (lymphatic anomalies, GLA, GSD, CCLA), drugs such as octreotide, interferon-α, and propranolol are not considered effective, and the validity of their use must be evaluated carefully.

#### Lay summary

GLA and GSD, which cause refractory chylous thoracic effusion, pericardial effusion, and respiratory disturbances, is extremely difficult to treat and can be lethal. They are treated by combinations of surgery, sclerotherapy, and radiotherapy as well as nutritional therapy and drug therapy, but none of them has been proved to be consistently effective. Among these treatments, oral sirolimus therapy has been highly effective with generally mild adverse reactions and is considered preferentially. It is the only drug approved as a treatment for this disease and is considered potentially promising as its first-line treatment. However, there has not been a report that complete remission was obtained by this therapy, and further clarification of unsettled questions such as severe adverse reactions and long-term risk of complications is needed.

### CQ36: Are Kampo formulas effective for LM?

Recommendation: Although there are many reports that Kampo formulas were effective, no research papers that can reliably evaluate their effectiveness. However, as complications such as adverse reactions are rare, their use is worth considering.

Strength of recommendation: 2 (weak)

Evidence: C (weak)

#### Process of preparation of recommendation

##### 1．Circumstances that make CQ an important clinical issue

Recently, there have been sporadic reports that Kampo formulas were effective for LM. Many of them were about *Eppikajutsuto*, but its effectiveness is uncertain. Evaluation of the efficacy and safety of Kampo formulas as a drug therapy is considered to lead to further increases in treatment options for LM.

##### 2．Evaluation of evidence

###### <Literature search>

Nineteen papers in Japanese and 9 papers in European languages were reviewed. As a result of primary screening, 5 papers in Japanese and 5 papers in European languages were subjected to secondary screening. Of these references, those that had contents relevant to this CQ were all case series or case reports and did not include RCT. Therefore, the results and discussions in the reports judged to be useful for the preparation of the recommendation statement were integrated and used as review data, although their evidence level is low.

###### <Evaluation>

The effectiveness of Kampo formulas for LM was evaluated from the following viewpoints.

I．Treatment responses

Size

II．Complications

The contents of the references concerning the efficacy of Kampo formulas as a treatment for LM were summarized from these viewpoints.

However, the above references included those reporting treatments using Kampo formulas performed before or after surgical resection or sclerotherapy as well as those reporting the results of treatment using Kampo formulas alone. No report directly compared treatment with Kampo formulas with observation without treatment, sclerotherapy, or surgical resection.

Many papers analyzed only superficial lesions, but there were also reports about patients with non-superficial lesions such as tongue, mediastinal, and retroperitoneal lesions.

Typing of lesions varied among reports with some classifying lesions into macrocystic and microcystic LM and others adopting the ISSVA classification. The methods for efficacy evaluation also varied and included ultrasound, CT, and MRI.

*Eppikajutsuto* was the only drug whose therapeutic effect was evaluated in the papers subjected to secondary screening. There were several reports that touched on the use of *Ogikenchuto*, but they were no sufficient for the evaluation of its therapeutic effect in this CQ. Also, there was no paper that evaluated the dose, administration period, and number of administrations of each drug in the literature reviewed this time.

Such differences in the patient background and contents of treatment must be taken into consideration in evaluating the efficacy of Kampo formulas.

####### I．Treatment responses

######## Size

There were 3 case series that mentioned the reduction of lesion size of LM by Kampo formulas^681,767,768)^. In the report by Hashizume *et al*.^767)^, the regression rate was measured by MRI in 8 cases of LM treated with *Eppikajutsuto* (0.3 g/kg/day), and a significant size-reducing effect with a mean regression rate of 54.5 ± 38.3% (median: 53.1%, P = 0.012) compared with the values before treatment was observed. According to the ISSVA classification, the mean regression rate (after treatment/before treatment) was 73.6 ± 27.0% (median: 77.1%, P = 0.145) in 4 cases of macrocystic LM and 35.4 ± 41.5% (median: 15.8%, P = 0.053) in 4 cases of mixed LM, showing differences in the size-reducing effect according to properties of the lesion. Goto *et al*.^681)^ measured the regression rate (1-after treatment/before treatment) by MRI in 10 cases of LM (4 cases of macrocystic LM, 2 cases of microcystic LM, 4 cases of mixed LM) treated with *Eppikajutsuto* (at 0.3 g/kg/day for the first 3 months, continued in some cases by increasing the dose to 0.5 mg/kg/day for 3 additional months) and observed a mean regression rate of 83%. According to the ISSVA classification, they reported that the regression rate was 100% (disappearance of all lesions) for macrocystic LM, 33.5% for microcystic LM, and 63% for mixed LM. Sato *et al*.^768)^ evaluated 10 cases of macrocystic LM treated with *Eppikajutsuto* (0.2 g/kg/day) by ultrasound or CT and reported that the lesions disappeared in 5, and the lesion size decreased to ≤1/3 in 5, but increased after treatment in only 1.

There were case reports in which reductions of lesion size were observed by additional treatment using Kampo formulas after OK-432 sclerotherapy^769,770)^ and for retroperitoneal LM after infection^771,772)^.

There were also case series and case reports that the treatment was ineffective^681,767,773)^.

####### II．Complications

There was no literature reporting complications of treatment for LM using Kampo formulas.

###### <Conclusion>

In evaluating the CQ “Are Kampo formulas effective for LM?”, analysis was made from the viewpoints of the therapeutic effect and complications of oral administration of Kampo formulas, but there was no paper with a high evidence level. Regression of the lesion and symptomatic relief were achieved by oral administration of Kampo formulas in some cases, but the response rate varied among reports. No clear complication of treatment was reported.

Based on the above, Kampo formulas are not considered at present to be effective in all cases of LM, and there was no reference that verifies their effectiveness. However, as no adverse effects or complications of Kampo formulas have been reported, their use is considered to deserve consideration if there is no other treatment. The evaluation of this CQ for the future is considered to need validation by a research design with a high evidence level such as RCT.

##### 3．Evaluation of balance of benefits and risks

Presently, Kampo formulas are not considered effective in all cases of LM, and the evidence to validate their effectiveness is insufficient, but they cannot be dismissed as ineffective. At the same time, there have been few reports of their adverse effects or complications, and their risks are negligible. Therefore, their benefits may surpass their minimal risks.

##### 4．Patients’ values/wishes

If sclerotherapy or radical surgical treatment is difficult to perform, symptomatic relief and reduction of lesion size, which makes surgical resection possible, by oral administration of Kampo formulas conform to the patients’ wishes. Currently, drug therapies covered by National Health Insurance in Japan are limited, and increasing the options of drug therapies for LM is considered to conform to the patients’ values/wishes unless their adverse effects are life-threatening or markedly deteriorates QOL.

##### 5．Cost assessment, assessment of external validity of intervention

Presently, in Japan, *Eppikajutsuto* is used for LM patients only for the symptom called “edema”, but details of its indications or protocol have not been established, and future reports are awaited. In addition, compared with sirolimus, which is presently covered by National Health Insurance in Japan, the cost is very low, and adverse effects are few. It is hoped for the future that Kampo formulas are approved in Japan through validation of this CQ by research designs with a high evidence level such as RCT.

#### Lay summary

Kampo formulas have been used for the treatment of LM for about 10 years and are drugs with a short history of use for LM. Although there are several reports of patients in whom they were effective for reducing the lesion size, they are used nearly exclusively in Japan, and the number of reported cases is small. At present, there is no research paper reliable for the evaluation of their effectiveness. However, reports of adverse effects or complications associated with treatment are few, and disadvantages of oral therapy are expected to be small. Therefore, the use of Kampo formulas is considered worth evaluating as a treatment option. For the future, the magnitude of their effects is expected to be clarified through more precise clinical studies.

### CQ37: What treatments are effective for LM of the skin/mucosa (lymphangioma circumscriptum) ?

Recommendation: Treatments including surgical resection, sclerotherapy, CO_2_ laser, radiofrequency ablation, electrocoagulation, and topical sirolimus have been reported in the past. Each of these treatments is effective but may cause complications and recurrence, and the treatment needs to be selected carefully according to symptoms.

Strength of recommendation: 2 (weak)

Evidence: D (very weak)

#### Process of preparation of recommendation

##### 1．Circumstances that make CQ an important clinical issue

Of LMs, lesions with the cyst exposed on the body surface such as the skin/mucosa (lymphangioma circumscriptum, LC) may rupture and cause lymphorrhea or show a reddish black color due to blood mixed in the lymph inside. They may be only superficial but may often be continuous with a large lesion in a deeper area. They are superficial lesions but are often difficult to treat. Since they have different aspects compared with deep lesions, understanding of their nature and selection of treatment are necessary. Knowledge available at present was compiled.

##### 2．Evaluation of evidence

###### <Literature search>

As a result of a systematic literature search, 161 papers in Japanese and 295 papers in European languages were retrieved. By primary screening of these papers, 4 papers in Japanese and 30 papers in European languages were subjected to secondary screening for this CQ.

Of these references, 11, which were all in European languages, mentioned treatment of LC. They included 2 systematic reviews, but all the rest were case series or case reports. Moreover, hand search for the literature concerning topical sirolimus was made, the 3 papers in European languages extracted were screened, and all were included in the references as case series. The results and discussions in these 14 papers were integrated as review data.

###### <Evaluation>

[Cases described in the reviewed literature]

LMs are classified into macrocystic LM, microcystic LM, and LC. In this CQ, the literature that contained cases clinically diagnosed as LC was analyzed.

###### [Evaluation of case series]

As a result of screening of the literature, surgical resection, CO_2_ laser, radiofrequency ablation, electrocoagulation, sclerotherapy, and topical sirolimus were mentioned as treatments for LC, and they were evaluated individually as below.

####### ■Surgical resection

In the systematic review by Vlastos *et al.*, congenital LC (11 cases) and acquired LC (20 cases) were extracted. All cases were females, and the site of the lesion was the vulva. Surgical resection was performed in 15 (48.4%) of the 31 cases, and recurrence was observed in 3 (20.0%). No complication of treatment was reported^774)^.

In the case series at a single center reported by Browse *et al.*, 29 patients (13 males and 16 females) were reported, their median age was 13 years (1–31 years), and the lesions involved the upper part of the body (chest, neck, upper arm, axilla) in 11, lower part of the body (hip, inguinal region, thigh, buttocks) in 16, tongue in 1, and big toe in 1. Small lesions <10 cm in diameter were completely resected in 6, but lesions ≥10 cm were not completely resected in 19. The lesions in the tongue and the big toe could not be resected for anatomical reasons. Of the 21 patients in whom the lesions could not be completely resected, 5 showed subcutaneous hypertrophy, and 6 underwent surgery 2 or more times, and no recurrence was observed in 1^775)^.

In the case report at a single center by Bauer *et al.*, 3 cases (1 male and 2 females) aged 14–31 years were reported, and lesions were observed in the upper limb, axilla, and abdominal region. After surgical resection, recurrence was observed in all cases, but no further recurrence was noted after re-resection^776)^.

####### ■CO_2_ laser

In the case report by Shumaker *et al.*, a 7-year-old boy with LC in the left neck region was reported. After CO_2_ laser treatment, leakage of lymph and secretion of effusion were alleviated, and the lesion nearly disappeared. After 6 months, recurrence of a lesion 1–2 mm in diameter was observed, but the patient was followed up without treatment^777)^.

In the systematic review by Savas *et al.*, 28 cases (10 males and 18 females) were extracted from16 papers (5 reports of case series and 11 case reports). The lesions involved the perineal region (11 case), scrotum (5 cases), and areas including the thigh, buttocks, axilla, hips, chest wall, and thoracic region. After CO_2_ laser treatment (follow-up period: 4 months–3 years), 8 were free of recurrence, but 10 showed partial recurrence, and 2 showed complete recurrence. Regarding treatment-related complications, depigmentation, scarring, and keloid were observed^778)^.

In the report of a case series at a single center by Swanson *et al.*, 6 cases of LC of the penis were reported (aged 16–73 years). Four of them underwent CO_2_ laser treatment, but all had recurrence. There was no mention about treatment-related complictions^779)^.

In the systematic review by Vlastos *et al.*, 11 cases of congenital LC and 20 cases of acquired LC with lesions in the external genitalia, all of whom were females, were extracted. Those with congenital LC were aged 14–76 years, and those with acquired LC were aged 9–75 years. CO_2_ laser treatment was performed in 1 case of congenital LC and 3 cases of acquired LC, with recurrence observed in 3. There was no mention about treatment-related complications^774)^.

####### ■Radiofrequency ablation

In the systematic review by Ekelem *et al.*, 54 papers were analyzed, and 72 LC cases reported in 10 papers were compiled. All of these patients underwent radiofrequency ablation, and a ≥50% (maximum 88%) lesion size reduction was observed in all cases. As treatment-related complications, swelling (16 cases), depigmentation (17 cases), scarring (5 cases), ulcer (7 cases), intermittent bleeding (3 cases), and infection (1 case) were observed. Also, recurrence was observed in 66% of the cases^780)^.

In the multicenter case series reported by Subhadarshani *et al.*, 9 patients (5 males and 4 females) were reported. Their age was unknown, and the lesions involved the abdominal region (2 cases), tongue (1 case), arm (2 cases), thigh (1 case), lower leg (1 case), and vulva (1 case). After radiofrequency ablation, lymph leakage and bleeding were alleviated in all cases. Regarding treatment-related complications, transient edema around the area of treatment (all case) and thermal injury (2 cases) were observed. During a follow-up period of 2–49 months, all patients were free of recurrence^781)^.

####### ■Electrocoagulation

In the single-center case series reported by Yang *et al.*, 12 cases were reported [males:females = 1:2, mean age: 13.6 years (4–31 years)]. The lesions were located in the oral mucosa (2 cases), tongue (2 cases), trunk (4 cases), upper limb (2 cases), and lower limb (2 cases). After electrocoagulation, lymph leakage, rupture, scarring, and bleeding were alleviated in all cases. The lesion size was reduced by ≥90% in all patients, and the lesions disappeared in 5 patients. As treatment-related complications, pain (9 cases) and ulcer (1 case) were observed. While no recurrence was observed, the posttreatment follow-up period was short, being a mean of 8 months, and the assessment was insufficient^782)^.

####### ■Sclerotherapy

The drug used for sclerotherapy was 3% polidocanol. No paper that evaluated the administration method or number of administrations of 3% polidocanol in sclerotherapy for LC was included in the literature reviewed this time. One paper referred to regression of the lesion and complications by sclerotherapy^780)^. In the case series reported by Niti *et al.*, the regression rate was ≥70% (1 case), ≥60% (1 case), and ≥50% (2 cases) in 4 cases of LC treated with 3% polidocanol alone. Leakage of effusion was alleviated in 2 cases. As for complications, pain (all cases), swelling (1 case), and ulcer formation (1 case) were observed. Recurrence was observed at least in 1 case 48 months after treatment. In 10 cases of LC treated by sclerotherapy in combination with radiofrequency ablation, the regression rate was 100% (complete disappearance)(2 cases), ≥95% (1 case), ≥90% (6 cases), and ≥50% (1 case). Effusion and scarring disappeared in all cases. As for complications, swelling (9 cases), pain (3 cases), and ulcer formation (2 cases) were observed. Recurrence was observed in at least 6 of the 9 cases after treatment (6–60 months). There was no report in which OK-432 was used as a sclerosing agent^783)^.

####### ■Topical sirolimus

There were 4 case series studies of topical sirolimus therapy for LC or LM of the skin/mucosa (a total of 37 cases). Badia *et al.*, applied a 1% topical sirolimus preparation to the lesion 2 times a day and observed improvements in symptoms such as lymphorrhea, bleeding, itching, and pain in 10 of 11 cases without clear adverse effects^486)^. Le Sage *et al.*, applied a 0.1% sirolimus preparation to the lesion in 6 cases of superficial vascular lesion and observed decreases in the size and number of LC lesions and effusion from the lesions in 3 of 4 cases^487)^. Dodds *et al.*, applied a 1–3% preparation of sirolimus to superficial vascular lesions in 18 cases. Of these cases, improvements in lymphorrhea, bleeding, infection, induration, pain, and cosmesis were observed in 1 case of those with LM of the skim/mucosa showing lymphorrhea in the trunk (5 cases), limbs (11 cases), and tongue (1 case) and all 11 cases of capillary/lymphatic/venous malformations^784)^. García-Montero *et al.*, applied 0.4–1% sirolimus to LC lesions in 11 cases for a median of 16.1 months and reported clinical changes in morbid appearance (reduction of lesion area in 36%, color fading in 72%, reduction of lymph follicle volume in 73%) in all cases and alleviation of the symptoms observed in 9 cases (lymphorrhea in 7, resolution of pain in 4, reduction of exacerbation of inflammation in 2, resolution of infection in 2)^785)^. In all reports, improvements in the lesion and associated symptoms were observed in a high percentage of cases with local irritation being observed in 2 of the above 37 cases as adverse effects^487,785)^.

###### <Conclusion>

For the evaluation of the CQ “What treatments are effective for LM of the skin/mucosa (lymphangioma circumscriptum)?”, papers extracted by systematic literature search were analyzed, but no paper with a high evidence level was found. From these papers, 60 cases of surgical resection, 37 cases of CO_2_ laser treatment, 19 cases of radiofrequency ablation, 12 cases of electrocoagulation, 14 cases of sclerotherapy, and 37 cases of topical sirolimus therapy were extracted. All these treatments have been reported to be effective in some cases. However, it was difficult to discuss their efficacy or safety based on objective figures. For the future, research with a high evidence level is necessary.

##### 3．Evaluation of balance of benefits and risks

Concerning each treatment for LM of the skin/mucosa, no research with a high evidence level that permitted objective assessment/comparison of the effect (benefit) and complication/recurrence (risk) was observed. However, while some therapeutic effects including the disappearance of the lesion, alleviation of associated symptoms, and improvement in QOL were often observed, it has been shown that the invasiveness of each treatment is limited due to the superficial nature of the lesion and that the possibility of serious complications is practically nil. The certainty of benefit is unclear, but the risk of small.

##### 4．Patients’ values/wishes

Symptoms of LM of the skin/mucosa are often exacerbated gradually. However, as there is no effective and reliable treatment, symptomatic management is selected. Since all treatments extracted in the present review are all mildly invasive and are expected to be more or less effective, they may be selected according to the patients’ values/wishes, particularly, if the symptoms are strong or are aggravating. However, it must be remembered that not all patients share the same values.

##### 5．Cost assessment, assessment of external validity of intervention

In Japan, surgical resection is covered by National Health Insurance and is widely performed. On the other hand, none of the treatments extracted in the present review other than surgical resection is not established and is not approved as an insured treatment. (While sclerotherapy using OK-432 is covered by National Health Insurance in Japan, there was no report of its application to this disorder.) The balance between the magnitude of invasion and effect is also uncertain, and the evaluation of the cost and validity of intervention may vary with the severity of symptoms.

#### Lay summary

LC is LM with cysts exposed on the body surface such as the skin and mucosa. Because of the thinness of the skin on the surface, lymph inside the cyst can be seen through it, and the cysts may appear dark red if lymph is mixed with blood or rupture causing leakage of lymph. The lesions may not only be exposed on the surface but also be continuous with a large lesion in a deep area and are relatively difficult to treat. Treatments including surgical resection, CO_2_ laser treatment, radiofrequency ablation, electrocoagulation, sclerotherapy, and topical sirolimus therapy have been attempted, and while they have effects such as regression of the lesion and alleviation of lymph leakage, there is concern over the frequent occurrence of skin complications and recurrence. It is necessary to carefully evaluate the necessity of treatment and appropriate treatments depending on the severity of symptoms.

### CQ38: Is compression therapy effective for primary lymphedema?

Recommendation: There are many reports that compression therapy was effective for symptomatic relief, but data concerning the therapeutic effect of compression alone are insufficient. Since compression may cause mild complications and restrictions of daily living, it is recommended to be performed with appropriate guidance on its implementation.

Strength of recommendation: 2 (weak)

Evidence: C (weak)

#### Process of preparation of recommendation

##### 1．Circumstances that make CQ an important clinical issue

Primary lymphedema is an intractable disease in which edema of varying severity appears at a wide range of time points due to lymphatic vessel dysplasia and functional insufficiency. It is distinguished from secondary lymphedema, which occurs after surgery or radiotherapy for cancer. Possibly responsible gene variations have been disclosed in succession, but the cause of the disease is unclear in many cases. Therefore, radical treatment of the disease is difficult at present, and symptomatic approaches depending on the condition is the mainstay of treatment. Among them, whether compression therapy, which is widely practiced for secondary lymphedema, is equally effective for primary lymphedema was evaluated as a CQ by systematically reviewing the literature.

##### 2．Evaluation of evidence

###### <Literature search>

One hundred and twenty-three papers in Japanese and 366 papers in European languages collected by systematic literature search were subjected to primary screening, and 4 papers in Japanese and 37 papers in European languages were subjected to secondary screening. The literature reviewed included many case series or case reports with contents corresponding to this CQ but no RCT or systematic review exclusively targeting primary lymphedema. Therefore, while the evidence level was low, all references judged to be useful for the preparation of the recommendation statement were evaluated.

###### <Evaluation>

The effectiveness of compression therapy for primary lymphedema was evaluated from the following viewpoints, and the contents of the references were compiled.

I．Treatment responses

A．Symptomatic relief (infection, bleeding, pain)

B．Regression of lesion

C．Improvements in cosmetics and QOL

II．Complications (swelling, pain, bleeding, infection, paralysis, others)

Attention to the following points is needed. Although primary lymphedema, which is the subject disease of this CQ, is differentiated from secondary lymphedema, which occurs after surgery or radiotherapy for cancer, many papers treated the 2 as a single disease group without distinguishing them. Moreover, primary lymphedema is classified into congenital, early-onset, and late-onset types, but few references mentioned their distinction. In addition, papers that mentioned the primary disease were small in number, the contents of references, if any, were restricted to epidemiology, and analysis concerning the therapeutic effect was not made.

The conventional methods employed as compression therapy were long or short-stretch banding, multilayer lymphoedema bandaging, and compression garments^786)^, there were also a few reports of the use of compression using a pneumatic compression pump^787-791)^. In this CQ, analysis was performed by regarding treatments using these methods inclusively as compression therapy, and all papers reported one or more of these methods performed alone or in combination. Also, there is few reports of compression therapy, which is the subject of this CQ, was performed alone, and it should be remembered that compression therapy is part of complex decongestive physical therapy (CDPT), in which techniques including exercise therapy, skin care, manual lymph drainage are performed in combination.

####### I．Treatment responses

######## A．Symptomatic relief (infection, bleeding, pain)

Four papers referred to symptomatic relief^790,792-794)^. Based on a questionnaire survey of 803 patients with primary lymphedema of the lower limb, Deng *et al.* reported improvements in pain, range of motion, and numbness of the affected limb by the use of compression garments^793)^. In addition, Blumberg *et al.* treated 100 patients with lymphedema (of whom 22% had primary lymphedema) by CDPT using a pneumatic compression pump and reported that CDPT not only significantly reduced infection and ulcer formation, but also resulted in symptomatic relief could be obtained in all patients^790)^. Ko *et al.* similarly reported that the incidence of infection was reduced in 299 patients with upper/lower limb lymphedema [including 3 cases of primary lymphedema of the upper limb (2%) and 93 cases of primary lymphedema of the lower limb (61.3%)] by CDPT^794)^.

######## B．Regression of lesion

Thirteen papers referred to regression of lesions. Regression was evaluated according to the volume of the affected limb in the largest number of papers^789,794-800)^ but according to the girth of the affected limb^790,791)^, pressure of compression of the affected limb during CDPT^801)^, internal pressure of lymphatic vessel^802)^, and patient’s body weight^803)^ in others. Of these papers, as a reference that included many cases of primary lymphedema, Vignes *et al.* reported that CDPT was performed in 222 patients with primary lymphedema of the lower limb (median duration of treatment: 11 days), resulting in a 34% reduction of the volume of the affected limb^795)^. According to Ko *et al.*, who treated 299 patients with upper/lower limb lymphedema [including 3 cases of primary lymphedema of the upper limb (2%) and 93 cases of primary lymphedema of the lower limb (61.3%)] by CDPT, reported that the volume reduction rate of the affected limb was 59.1% in the upper limb group and 67.7% in the lower limb group and that the volume of the affected limb after compression therapy could be maintained even after a 9-month follow-up period with satisfactory patient compliance^794)^.

######## C．Improvements in cosmetics and QOL

Improvement in cosmetic appearance is correlated with decrease in lesion size. However, regarding QOL, according to a questionnaire survey of 100 patients with lymphedema (22% of whom had primary lymphedema) treated by CDPT, marked, moderate, and mild improvements in QOL were observed in 54%, 35%, and 11%, respectively, and 90% of all patients answered that they would recommend similar treatment^790)^. There was also a report that significant improvements in QOL could be achieved in both physical activities and social life by CDPT in 34 patients with lymphedema (including 18 with primary lymphedema)^791)^.

####### II．Complications (swelling, pain, bleeding, infection, paralysis, others)

Three references touched on complications related to compression therapy^789,798,804)^. Quéré *et al.* treated 306 patients with lymphedema (primary lymphedema in 21.6%) by the CDPT protocol and reported that the protocol could be completed without complications in 269^789)^. However, reddening of the skin was observed in 18.4%, that skin imprint mark was observed in 15.7% of the patients. They also reported that 6.3%, 4.0%, and 3.6% of the patients exhibited infection, blister formation, and intolerance of the skin, which requires discontinuation of intervention respectively. In addition, according to the report by Moffatt *et al.*, who treated 82 patients with upper/lower limb lymphedema [including 19 (45%) with primary lymphedema of the lower limb] using short-stretch bandages, 11 (27.5%) of the cases of lower limb lymphedema developed unexpected complications^798)^. They included discomfort associated with compression therapy, skin troubles such as itching, skin damages, reddening, and blister formation, and swelling of joints. No complication with a significantly high incidence has been reported. On the other hand, Boris *et al.* compared those treated with and without a pneumatic compression pump among 128 patients with lower limb lymphedema and reported genital lymphedema occurred significantly more frequently in those treated with the pump^804)^. Also, careful attention is needed in compression therapy using compression garments bandages as their inadequate application may cause peripheral disorders such as venostasis.

###### <Conclusion>

(I) Treatment responses and (II) complications of compression therapy for primary lymphedema were analyzed. Papers that reported therapeutic intervention by compression therapy alone or evaluated the effect by distinguishing primary and secondary lymphedema were extremely rare, and there was no paper with a high evidence level. However, there were many reports about regression of the lesion or improvements in symptoms or QOL.

According to the systematic review concerning non-surgical management/treatment for lymphedema by Rodrick *et al.*, compression therapy was not mentioned as a recommendable treatment^805)^. However, concerning surgical treatments, a systematic review published in 1985 recommended surgery for patients in a serious condition^806)^. Although there have been reports of debulking surgery and lymphatic or lymphaticovenous anastomosis, surgical treatment is not considered to have priority over conservative treatments^807)^. Moreover, Schook *et al.*, who treated 142 non-adult cases of lymphedema (including 138 cases of primary lymphedema), reported that surgical treatments for limb lesions were performed in 6% of the cases and that the disease could be controlled by conservative treatments alone in many cases^788)^.

In addition, compression therapy is considered to be performed relatively safely while there are complications such as skin troubles.

However, as Okajima *et al.* reported that QOL of patients with primary lymphedema in daily living is low even with selfcare such as compression therapy and exercise therapy^808)^, it is considered that CDPT including compression therapy should be performed by those who have received specialist training^802)^, and patient guidance was given by experts such as physical therapists in many papers concerning compression therapy reviewed at this time. Also, in children, adjustment of compression garments with growth, burden of hospital visits at each time of garment adjustment, burden of garment consumption for toilet training, their urge to remove the garments, and risk of infection due to injury have been suggested as problems^809)^, so the support system for patients’ families also needs be evaluated.

###### <Summary>

Regarding primary lymphedema, the effects of compression therapy have not been evaluated by research with a high evidence level at present. Although it is difficult to determine the indications of compression therapy by setting criteria, none of the references strongly objects to compression therapy in consideration of the degree of invasiveness. Validation by a research design with a high evidence level such as RCT specifically targeting primary lymphedema is considered necessary for the evaluation of this CQ.

##### 3．Evaluation of balance of benefits and risks

For primary lymphedema, compression therapy has been reported to have some therapeutic effects such as regression of lesion, alleviation of associated symptoms, and improvements in QOL. While the evidence is not sufficient, the treatment is judged to have benefits. In contrast, the invasiveness of compression therapy is relatively low with very few serious complications, and its risk are extremely low. The benefits may outweigh the very low risks.

##### 4．Patients’ values/wishes

Primary lymphedema is often unexplained, and the symptoms progress in many cases, but no treatment is consistently effective, and symptomatic care is the only choice. Among symptomatic treatments, compression therapy, which is less invasive, relatively free of complications, and expected to be effective to an extent, conforms to the patients’ values/wishes, particularly, when symptoms are severe or are aggravating. However, prolonged compression therapy may not be tolerated, particularly, in children, and all patients are not considered to share the same values.

##### 5．Cost assessment, assessment of external validity of intervention

A major cost of compression therapy is the expenses on materials for elastic garments. In Japan, elastic garments became an item of medical treatment cost to be supplied in 2008, but braces that are adjusted to changes with growth and special morphology of the affected area are necessary, and they may need to be customized. Evaluation of the validity of intervention and its therapeutic effect remains insufficient.

#### Lay summary

There are a number of reports that compression therapy for primary lymphedema has therapeutic effects and causes regression of lesion, alleviation of associated symptoms, and improvements in QOL. Also, this treatment has been shown to be relatively non-invasive and unlikely to cause severe complications, and there is no major reason for refraining from its implementation if it is performed properly with other physical therapies. However, it still lacks validation of effectiveness through sufficient scientific evaluation. Also, there is the possibility of complications, although they are usually mild, and the therapy restricts daily activities. Since the needs and values vary among patients, it is recommended to decide on whether to actually try compression therapy by sufficient consultation with a physician and to perform it under appropriate guidance.

## Conclusion

The practice guidelines for vascular anomalies have been prepared as the evidence-based guidelines for the management of vascular anomalies.

**Footnote:** *This is the secondary publication of the guidelines that were published in Japanese. We have obtained consent from all authors for secondary publication. These guidelines are an excerpt from the full version of the Japanese guidelines. Consequently, figures, tables, and supplementary information included in the original Japanese version are not included in this English version. For the same reason, the number of references is fewer than in the full Japanese-language version. This article has been co-published in Journal of Plastic and Reconstructive Surgery, Japanese Journal of Radiology, Pediatrics International, Surgery Today, and The Journal of Dermatology based on the agreement of Editor-in-Chief of the five journals.*

**Conflicts of Interest:** The conflict of interest of the guidelines preparation organization was managed by the Guidelines Executive Committee. Masatoshi Jinnin and Tamihiro Kawakami are editorial board members of the Journal of Dermatology.
